# Positional Tuning of Photophysics and Catalysis in Methoxy-Substituted Heteroleptic Copper(I) Complexes

**DOI:** 10.1021/acs.inorgchem.5c04739

**Published:** 2025-12-02

**Authors:** Kurt J. Haseloff, Katharina Rediger, Mohammad D. Mandourah, Max Wolf, Christian Kleeberg, Stefanie Tschierlei, Maria Wächtler, Michael Karnahl

**Affiliations:** † Department of Energy Conversion, Institute of Physical and Theoretical Chemistry, 26527 Technische Universität Braunschweig, Rebenring 31, Braunschweig 38106, Germany; ‡ Department of Chemistry, Rheinland-Pfälzische Technische Universität Kaiserslautern- Landau, Erwin-Schrödinger-Straße 52, Kaiserslautern 67663, Germany; § Institute of Inorganic and Analytical Chemistry, Technische Universität Braunschweig, Hagenring 30, Braunschweig 38106, Germany; ∥ Institute of Physical Chemistry, Christian-Albrechts-Universität zu Kiel, May-Eyth-Straße 1, Kiel 24118, Germany

## Abstract

A series of heteroleptic copper­(I) photosensitizers based on methoxy-substituted 2,9-dimethyl-4,7-diphenyl-1,10-phenanthroline ligands was synthesized to investigate the influence of substitution patterns on structure and function. Methoxy groups were introduced in *ortho*-, *meta*-, and *para*-positions of the phenyl rings. Single-crystal X-ray diffraction and DFT calculations confirmed the expected tetrahedral geometry with position-dependent aryl torsion. Photophysical studies reveal that *ortho*/*para*-substitution enhances absorptivity, emission quantum yields, and excited-state lifetimes compared to the *meta*/unsubstituted complexes. The *ortho*-substituted complex shows the strongest electron-donating effect, reflected in the most cathodic ligand reduction $\left(E_{1/2}^{\mathrm{red}}=-2.11\;V\right)$ and the least oxidizing excited state potential (E* = 0.46 V). Temperature-dependent luminescence and emission lifetimes are consistent with thermally activated delayed fluorescence (TADF) across the series and reveal substitution-controlled singlet–triplet energy gaps Δ*E*_ST_. Complemented by step-scan FTIR studies, the predominant excited state was identified and analyzed, highlighting the impact of spin density location on both energy- and electron-transfer reactivity. The photocatalytic relevance was demonstrated in three benchmark reactions: singlet oxygen generation (energy transfer, demonstrated by the photooxidation of diphenylfuran to *cis*-dibenzoylethylene), hydrogen evolution from water, and reductive dehalogenation of aryl halides (electron transfer). In hydrogen evolution, the *para* isomer gave the highest initial rate and a TON of 590 at 20 h, while the *ortho* isomer remained active up to 36 h with a TON of 530. Stern-Volmer quenching in THF with TEA confirms a reductive pathway under these conditions. In the photocatalytic dehalogenation, activity trends were substrate-dependent, reflecting a balance between excited-state driving force (E*) and ground-state reducing power $\left(E_{1/2}^{\mathrm{red}}\right)$ . Together, these results establish clear position-property-performance relationships to guide Cu­(I) photosensitizer design.

## Graphical abstract

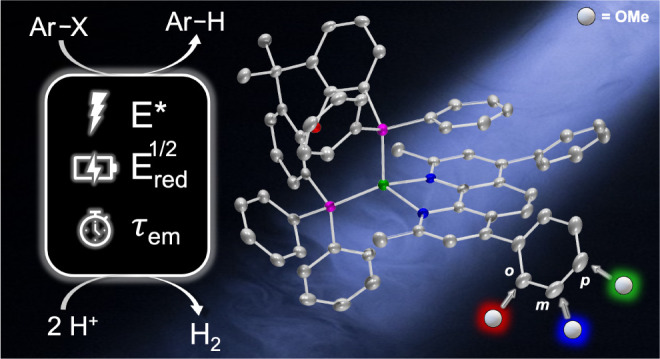

## Introduction

The continuous rise in atmospheric CO_2_ levels and the depletion of fossil fuels highlight the urgent need for sustainable technologies to generate solar fuels.^1–3^ Among various approaches, light-driven processes that convert sunlight into chemical energy have gained significant attention.^4–6^ Key to these processes are molecular photosensitizers (PS), which absorb visible light and initiate electron or energy transfer reactions required for catalytic transformations such as water splitting, CO_2_ reduction, or organic synthesis.^7–10^

Heteroleptic Cu­(I) complexes of the type [Cu­(N^N)­(P^P)]^+^ have emerged as promising alternatives to noble-metal systems based on Ru­(II) or Ir­(III) due to their Earth abundance and tunable photophysical properties.^11–16^ In particular, 2,9-dimethyl-1,10-phenanthroline derivatives allow for systematic ligand design to optimize absorption, redox behavior, and excited-state lifetimes.^12,17–20^

In recent years, the effects of substituents at various positions on the phenanthroline scaffold or on appended aryl groups have been intensively studied with respect to the structure-property relationships of these complexes.^21–24^ For instance, Takeda and coworkers demonstrated that electron-withdrawing groups such as nitro (−NO_2_), cyano (−CN), or trifluoromethyl (−CF_3_) at the 4,7-positions of phenanthroline can decrease emission lifetimes and quantum yields, yet enhance photocatalytic activity, attributed to increased oxidative driving forces.^25,26^ In contrast, electron-donating amine substituents have been shown to improve both the photophysical properties and catalytic performance.^21^

In our recent work, the effect of methoxyphenyl substituents introduced at different positions of the phenanthroline backbone was systematically explored.^18,23,24,27^ The substitution at the 4,7-positions leads to a pronounced increase in absorptivity, emission lifetime, and quantum yield, while similar groups in the 5,6-positions resulted in unfavorable properties and the formation of dark states.^18,23,24^ Our group recently extended these studies by systematically investigating the effect of *para*-substituted phenyl groups (-OMe, -F, −CF_3_) introduced at the 4,7- and 5,6-positions of the phenanthroline-based ligand.^18,23^ This structural motif, based on bathocuproine-type ligands (**C4-ref**, Figure 1), is known to enhance light absorption due to the extended π-system.^23,24,28^ The donor groups at the 4,7-positions significantly increased the absorptivity, emission lifetimes, and quantum yields (*e.g*. ε_MLCT_ (≈380 nm) from 5.2 × 10^3^ M^– 1^ cm^– 1^to 6.4 × 10^3^ M^– 1^ cm^– 1^, τ_em_ from 305 ns to 630 ns, Φ_em_ from 1.4% to 2.1% for OMe substituent compared to unsubstituted complex). In contrast, analogous donor substitution at the 5,6-positions resulted in short-lived emissive states or dark states with strongly diminished emission properties.^18,23,24^ In addition, complexes with CF_3_ substituents at the 4,7-positions exhibit the strongest excited-state oxidizing power (*E*_1/2_* = 0.58 V) and unique two-electron reducibility, highlighting the potential of such structural variations for photocatalytic applications, including singlet oxygen generation and reductive dehalogenation reactions.^23^ Building on these insights, the present study investigates a complementary structural motif: the positional influence of methoxy substituents on the phenyl rings attached to the phenanthroline backbone (Figure 1).

**Figure 1:**
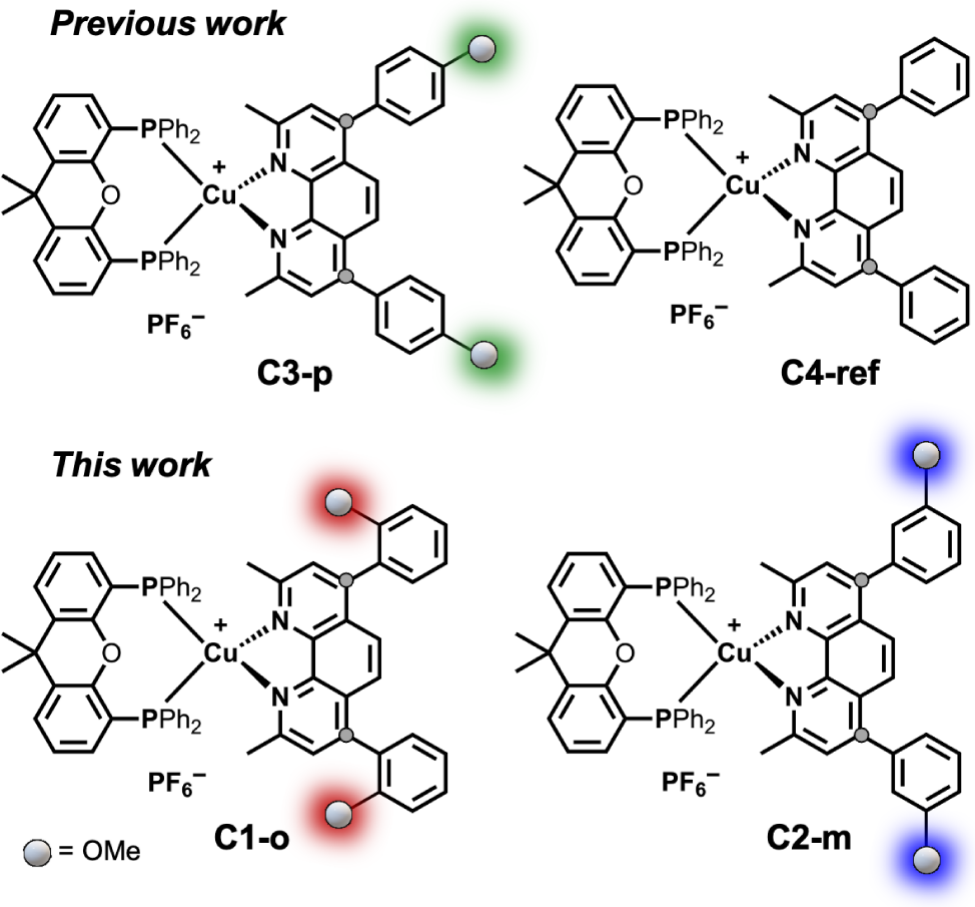
Molecular structures of the investigated heteroleptic Cu­(I) complexes. The newly synthesized compounds **C1-o** and **C2-m** feature methoxy substituents in *ortho*- and *meta*-position of the phenyl rings, respectively. The *para*-substituted complex **C3-p** and the unsubstituted reference **C4-ref** have been reported previously.^17,23^ The color code used here (red = **C1-o**, blue = **C2-m**, green = **C3-p**, black = **C4-ref**) is applied throughout this study.

Methoxy groups are of particular interest in this context, as they combine electron-donating strength with synthetic accessibility and structural versatility.^29–31^ In previous studies, such substituents have proven highly beneficial for tuning the photophysical properties of Cu­(I) photosensitizers, often leading to enhanced absorptivity, prolonged excited-state lifetimes, and improved catalytic performance. Therefore, a more detailed understanding of how the precise substitution pattern of methoxy groups translates into structure-property relationships is essential to further exploit their potential in photosensitizer design. Using a series of heteroleptic Cu­(I) complexes bearing methoxy groups in *ortho*- (**C1-o**), *meta*- (**C2-m**), and *para*-position (**C3-p**) of the phenyl rings (Figure 1), the electronic structure, photophysical properties, and catalytic behavior of the complexes are systematically investigated to elucidate the effects of these subtle structural modifications. A combination of single-crystal X-ray diffraction, steady-state and time-resolved spectroscopy, electrochemical analysis, and DFT calculations provide detailed insights into the structure-property relationships. Moreover, solid-state emission studies and time-resolved step-scan FTIR spectroscopy^32^ at cryogenic temperatures complement the solution-phase investigations, offering a comprehensive understanding of how fine-tuning the substitution pattern translates into modified properties relevant for photocatalysis. To demonstrate the practical relevance of these findings, the new complexes were further applied as photosensitizers in three representative light-driven model reactions:^23,33,34^ singlet oxygen generation (energy transfer, exemplified by the photooxidation of diphenylfuran to *cis*-dibenzoylethylene), photocatalytic hydrogen evolution from water, and the reductive dehalogenation of organic substrates (electron transfer). In addition, Stern-Volmer quenching was performed under the hydrogen-evolution conditions (THF/TEA) to establish the operative quenching pathway.

## Results and Discussion

### Synthesis

The methoxy-substituted 2,9-dimethyl-4,7-diphenyl-1,10-phenanthroline ligands were synthesized via Suzuki-Miyaura cross-coupling reactions.^18,35^ 4,7-dichloro-2,9-dimethyl-1,10-phenanthroline, prepared according to literature procedures,^36^ was reacted with substituted phenylboronic acids bearing methoxy groups in *ortho*- (B­(OH)_2_PhOMe_*o*_) or *meta*-position (B­(OH)_2_PhOMe_*m*_). The reactions were performed under inert atmosphere in a biphasic mixture of THF/water (1:1) using XPhos Pd G2 as the catalyst and K_3_PO_4_ as the base (Figure 2). After refluxing for 16 h at 75 °C and subsequent workup, the ligands **L1-o** and **L2-m** were obtained in isolated yields of 71% and 68%, respectively. The corresponding heteroleptic Cu­(I) complexes were synthesized following a well-established two-step, one-pot procedure.^18,34,37^ In the first step, the Cu­(I) precursor [Cu­(MeCN)_4_]­PF_6_ (1 eq., MeCN = acetonitrile) and xantphos (1 eq., xantphos = (9,9-Dimethyl-9*H*-xanthene-4,5-diyl)­bis­(diphenylphosphane)) were combined in dichloromethane (DCM) under argon and stirred at 45 °C overnight. After cooling to 0 °C, the respective diimine ligand was slowly added dropwise using a syringe pump (ca. 10 mL h^–1^) to suppress the formation of homoleptic byproducts.^18,20,38^ The reaction mixture was stirred for an additional 30 min at 0 °C and subsequently heated to 45 °C for 3 h. The resulting yellow solutions were treated with *n*-hexane to precipitate the products. **C1-o** and **C2-m** were isolated as yellow crystalline solids in excellent yields of 78% each. Full synthetic details and characterization data are provided in the Supporting Information (Chapter 2).

**Figure 2:**
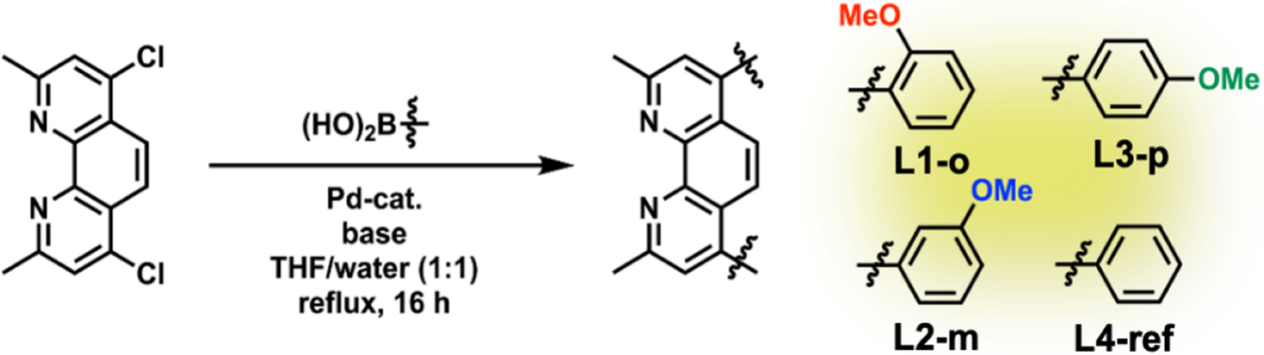
Synthesis procedure of the methoxy-substituted phenanthroline derivatives.

### Structural Characterization

The synthesized ligands and Cu­(I) complexes were analyzed by NMR spectroscopy (^1^H, ^13^C­{^1^H}, and ^31^P­{^1^H}), electrospray ionization high-resolution mass spectrometry (ESI-HRMS), and elemental analysis (EA), confirming their identity and high purity. In addition, single crystals suitable for X-ray diffraction analysis were obtained for complex **C2-m** by slow diffusion of *n*-hexane into a concentrated dichloromethane solution. Unfortunately, despite extensive attempts, no suitable crystals of **C1-o** could be obtained. To elucidate structural trends and the impact of the methoxy substitution pattern, DFT geometry optimizations were performed for all complexes (B3LYP-D3­(BJ)/def2-TZVP, COSMO solvent model for KBr).^39–44^ The optimized structures of **C1-o**, **C2-m**, **C3-p**, and **C4-ref** were compared to each other, and for **C2-m** also to the experimentally obtained X-ray structure (Figure 3 and Table 1).

**Table 1:** Selected Bond Lengths (Pm), Bite Angles (°), Interplane Angles between the N–Cu–N/P–Cu–P Planes (PP-Cu-NN, °) and Torsion Angles of Backbone Phenyl Rings τ_Sub_ (°) of the Structure of **C2-m** Obtained from X-ray Diffraction Studies and of the Structures of **C1-o**, **C3-p**, and **C4-ref** Predicted by DFT Calculations

	**C1-o**	**C2-m**		**C3-p**	**C4-ref**
	Calc.	Calc.	Exp.	Calc.	Calc.
Cu–N	211.4	212.0	208.8(16)	211.8	211.9
	211.8	212.2	211.7(17)	211.9	212.0
Cu–P	226.4	226.6	225.1(5)	226.7	226.5
	231.1	231.2	230.9(5)	231.4	231.2
C–O	137.0	137.7	137.0(3)	137.6	137.6
	137.6	137.7	137.1(2)	137.7	137.7
N–Cu–N	79.3	79.1	79.9(6)	79.1	79.2
P–Cu–P	118.7	118.7	119.7(2)	118.3	118.6
interplane	88.3	87.6	88.75	86.6	87.3
τ_Sub,1_	–59.9	–53.9	–75.3(3)	–126.5	–53.2
τ_Sub,2_	62.4	55.5	61.4(3)	53.1	54.7

**Figure 3:**
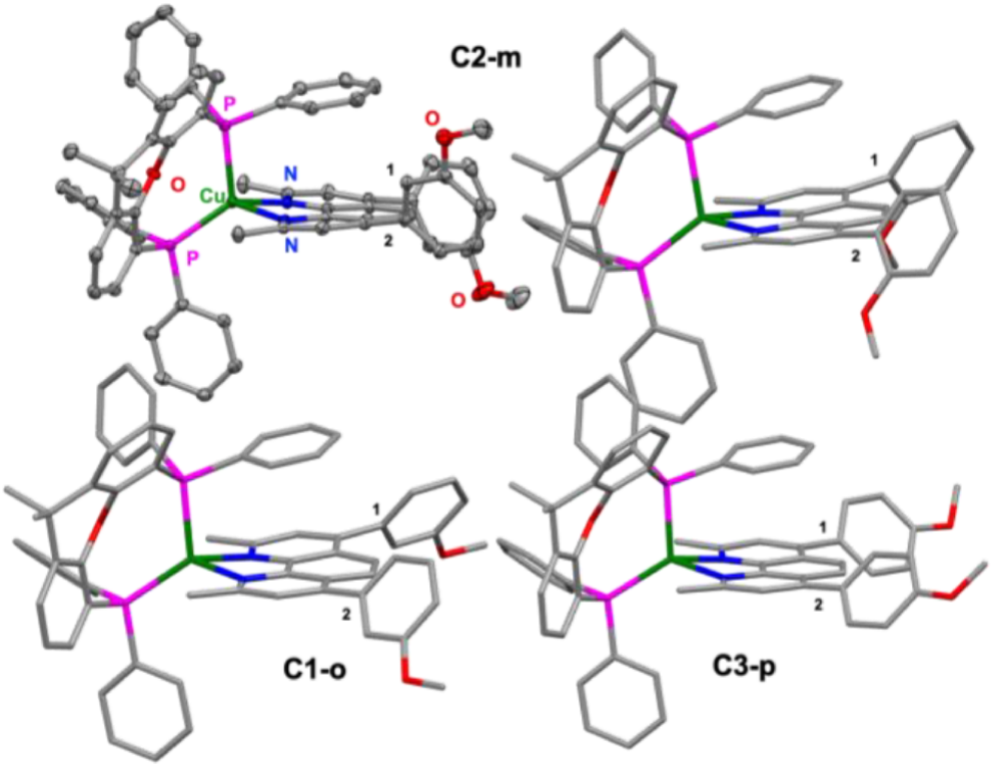
Solid-state structure of **C2-m** obtained from single crystal X-ray diffraction analysis (top left) and its optimized geometry predicted by DFT calculations (B3LYP-D3­(BJ)/def2-TZVP) (top right)). Optimized geometries of **C1-o** (bottom left) and **C3-p** (bottom right) are shown for comparison. The labels 1 and 2 refer to the torsion angle τ_sub_ of the two phenyl substituents (Table 1).

The solid-state structure of **C2-m** reveals the expected distorted tetrahedral coordination geometry around the Cu­(I) center, typical for this class of heteroleptic diimine-diphosphine complexes.^12,14,20,45^ The Cu–N bond lengths of 208.8 (16) pm and 211.7(17) pm are in excellent agreement with the DFT-predicted values (Table 1) and comparable to previously reported bathocuproine-based Cu­(I) photosensitizers.^15,23,24^ The Cu–P bond lengths of 225.1(5) pm and 230.9(5) pm also match well with the calculated values. Within the complex series **C1-o** shows the most contracted Cu–N bonds indicating a slightly stronger Cu–N interaction to the phenanthroline ligand. However, the coordination geometry including bond lengths and angles of the xantphos ligand remains essentially unaffected by the substitution pattern, in line with the DFT results. The bite angles N–Cu–N (79.9(6)°) and P–Cu–P (119.7(2)°) of **C2-m** obtained by X-ray diffraction analysis are consistent with literature values and indicate a slightly compressed diimine coordination compared to the ideal tetrahedron.^18,34,37,45^ Furthermore, the interplane angle between the N–Cu–N and P–Cu–P planes amounts to 88.8°, which is within the expected range and reflects the characteristic distortion imposed by the steric bulk of the xantphos ligand.^18,23,46^

Another important structural parameter is the torsion angle between the phenyl substituent and the phenanthroline backbone (τ_sub_), as it influences the degree of π-conjugation. For **C2-m**, two distinct torsion angles of −75.3(3)° and +61.4(3)° were determined, indicating significant twisting of the phenyl groups out of the diimine plane, which is similar to related systems.^24,47,48^ This structural parameter revealed the largest deviation from the prediction by DFT because it is sensitive to packing effects and the local environment.

As a result, DFT geometries predict a somewhat more planar arrangement. Further, the phenyl groups in **C3-p** are calculated to be oriented parallel to each other (τ_sub,1_ = −126.5 °), which is consistent with the previous reported X-ray structure for this complex.^24^

### Electrochemical Characterization

The electrochemical properties of the newly synthesized Cu­(I) complexes **C1-o** and **C2-m**, as well as the reference compounds **C3-p** and **C4-ref**, were investigated by cyclic voltammetry in deaerated acetonitrile (*cf*. Figures S53 and S54). Understanding the redox behavior of such complexes is crucial for evaluating their potential as photosensitizers in light-driven catalytic processes. All complexes exhibit at least one reversible reduction event, assigned to the reduction of the phenanthroline-based diimine ligand, as typically observed for this class of heteroleptic Cu­(I) photosensitizers.^14,23,25^ An irreversible oxidation is observed between 0.95 and 1.00 V (vs. Fc/Fc^+^) and is attributed to oxidative degradation initiated by Cu–P bond cleavage, as commonly reported for this class of complexes.^34,49,50^ The oxidation potentials (*E*_ox_) are in the range of 0.89 to 1.00 V (*cf*. Figure S53), showing only minor variations between the different substitution patterns.

In contrast, more pronounced differences are observed for the reduction potentials $(E_{1/2}^{\mathrm{red}}$ ). The *ortho*-methoxy substituted complex **C1-o** exhibits the most negative reduction potential (−2.11 V), followed by the *para*-substituted **C3-p** (−2.08 V) and the unsubstituted **C4-ref** (−2.04 V). The *meta*-substituted derivative **C2-m** has the least negative value (−2.03 V), comparable to the unsubstituted reference (Table 2). These results indicate that the methoxy group in *ortho*-position has the strongest electron-donating effect on the phenanthroline π-system, leading to a cathodic shift of the reduction potential. However, the *meta*-position has only a marginal influence on the electronic structure of the complex.

**Table 2:** Redox Potentials, Excited State Reduction Potentials $E_{1/2}^{* red}$ and the Zero–Zero Excitation Energy E^0,0^ (Both Calculations in SI, Chapter 12) of the Investigated Cu­(I) Complexes in Deaerated Acetonitrile

complex	E_ox_ ^c^	$E_{1/2}^{\mathrm{red}}$ ^c^,^d^	$E_{1/2}^{* red}$ ^e^	*E* ^0,0^
**C1-o**	+0.97 V	–2.11 V	0.46 V	2.57 eV
**C2-m**	+0.97 V	–2.03 V	0.50 V	2.53 eV
**C3-p**	+0.96 V	–2.08 V	0.48 V	2.56 eV
**C4-ref**	+0.89 V^a^	–2.04 V^b^	0.49 V	2.53 eV

a Taken from ref. 51.

b Taken from ref. 23.

c Referenced against ferrocene/ferricenium couple (Fc/Fc^+^).

d Only reversible events are listed.

e Estimation based on refs. 28,52 .

The observed trends are consistent with the structural and photophysical data (see below) and highlight the importance of the substitution pattern for fine-tuning the electronic properties. In addition to the first reduction process, all complexes exhibit further irreversible reduction events at more negative potentials (around −2.6 V and below), which are tentatively assigned to additional ligand-centered reductions involving the extended aromatic π-system. Due to their irreversibility and the fact that these processes occur outside the potential range of the photocatalytic reactions investigated, they are not considered further in the context of this study.

Furthermore, the excited-state redox potentials $\left(E_{1/2}^{*red}\right)$ were estimated using the Rehm–Weller approach.^28,52^ The required zero–zero excitation energy E^0,0^ (Table 2) has been calculated using the tangent-method as described in the SI (Chapter 12). **C1-o** exhibits the least oxidizing excited-state potential ( $E_{1/2}^{*red}$ = 0.46 V), followed by **C3-p** (0.48 V), **C4-ref** (0.49 V), and **C2-m** (0.50 V). This trend correlates with the electronic influence of the methoxy substituents and suggests that subtle structural modifications can affect the excited-state redox properties. Given the known high performance of **C3-p** in photocatalytic reductive dehalogenation reactions,^23^ the observed changes in excited-state redox potentials for **C1-o** and **C2-m** may provide valuable design principles for future optimization of copper-based photosensitizers.

### Photophysical Properties in Solution

The photophysical properties of all four Cu­(I) complexes, **C1-o**, **C2-m**, **C3-p**, and **C4-ref**, were investigated in deaerated acetonitrile to evaluate their potential as photosensitizers. Hence, absorption, emission, emission quantum yields, lifetimes, and photostability were systematically studied and compared to the established trends of analogous methoxy-substituted Cu­(I) photosensitizers.^18,23,24^

All complexes exhibit the characteristic absorption pattern of heteroleptic Cu­(I) photosensitizers (Figure 4), comprising intense π-π* transitions below 300 nm and broad metal-to-ligand charge transfer (MLCT) bands extending into the visible region. The methoxy position on the phenyl rings modulates the diimine-centered electronic structure, as seen in the MLCT maxima (**C3-p** 382 nm < **C1-o** 384 nm < **C2-m** 385 nm < **C4-ref** 387 nm, Table 3).^12,14^ Para-substitution increases the MLCT transition energy (larger E_00_), yielding a hypsochromic shift (387 → 382 nm) and a rise in molar absorptivity (ε ≈ 5.2 × 10^3^ → 6.4 × 10^3^ M^– 1^ cm^– 1^, Table 3 and Figure S29). This trend is consistent with the emission properties in solution (Figure 4).

**Table 3:** Summary of the Photophysical Properties of the Cu­(I) Complexes in Solution in Deaerated Acetonitrile (MeCN) at Room Temperature and in Solid Matrix (KBr)^a^

	λ_abs_ /nm (ε [10^3^ M^–1^cm^–1^])		λ_em_ ^c^ /ns				Φ_em_ ^c^,^d^/%		τ_em_ ^c^/ns		$\phi_{1_{O_{2}}}$ ^c^,^e^/%	
Complex	MeCN	KBr	MeCN	KBr (290 K)	KBr (170 K)	KBr (5 K)	MeCN	KBr	MeCN	KBr	DCM	KBr
**C1-o**	384 (4.7)	400	575	565	583	576	2.1	-	576	8700	45	-
**C2-m**	385 (4.9)	405	582	576	592	585	1.5	-	326	7890	40	-
**C3-p**	382^b^ (6.4)^b^	405	573^b^	557	574	568	2.1^b^	-	628	12050	45	-
**C4-ref**	387^b^ (5.2)^b^	404	578^b^	572	598	593	1.4^b^	-	305	20910	49	-

a Singlet oxygen quantum yields $\left(\phi_{1_{O_{2}}}\right)$ were determined in dichloromethane.

b Taken from ref. 23.

c Excited at λ = 390 nm.

d Ref. against **C4-ref** (1.4%).

e Ref. against phenalenone (98%).^71^

**Figure 4:**
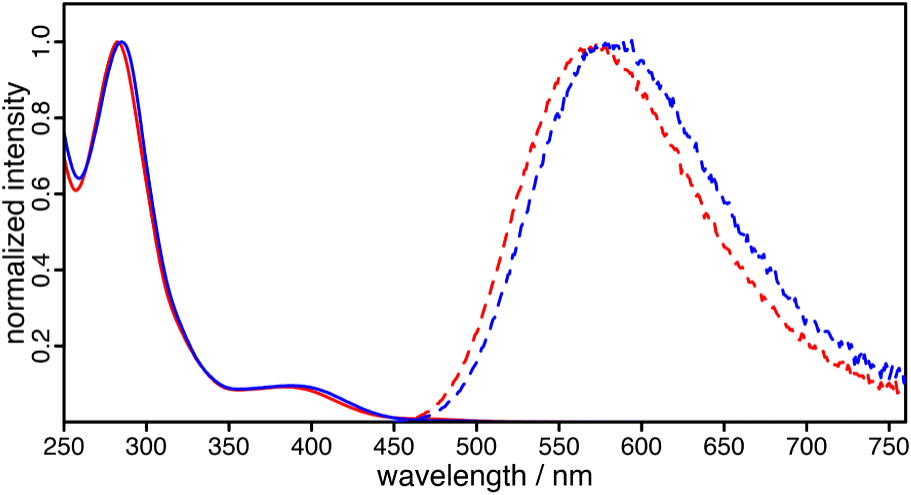
Normalized absorption spectra of **C1-o** (red solid line) and **C2-m** (blue solid line) showing the characteristic MLCT bands and respective normalized emission spectra of **C1-o** (red dashed line) and **C2-m** (blue dashed line) in deaerated acetonitrile measured at room temperature and excited at 390 nm.

**C3-p** shows the bluest emission maximum at 573 nm, followed by **C1-o** (575 nm), **C4-ref** (578 nm), and **C2-m** (582 nm, Table 3). This demonstrates that the methoxy substitution, particularly in *ortho*- and *para*-position, significantly influences the electronic structure, enabling fine-tuning of the optical properties through positional control on the phenyl ring due to their electron-donating effect ((+)-mesomeric effect).^30,31,53^ The methoxy group in the *ortho*- and *para*-position could shift electron density toward the phenanthroline ligand. In contrast, the *meta*-methoxy moieties only influence the phenanthroline by their negative inductive properties. This suggests that substituents with a dominating positive mesomeric effect in the *ortho*-position lead to an increased electron density, shifting the emission maximum toward higher energies because of increased excited state energy level.

The photostability of the newly synthesized complexes **C1-o** and **C2-m** was evaluated by monitoring UV/vis changes in deaerated acetonitrile under continuous visible-light irradiation (λ > 380 nm, 100 mW cm^–2^) over 1 h. Both complexes show only minor spectral changes (*cf*. Figure S56), confirming their stability under the applied conditions. These results are consistent with the high photostability previously reported for the related complexes **C3-p** and **C4-ref**.^23^

The emission quantum yields (Φ_em_) follow the established trend observed for related systems.^23,27^ The *ortho*- and *para*-substituted complexes **C1-o** and **C3-p** exhibit the highest quantum yields of 2.1%, while **C2-**m (*meta*) and **C4-ref** (unsubstituted) show slightly lower values of 1.5% and 1.4%, respectively. The same trend is reflected in the emission lifetimes (τ_em_), with **C3-p** (628 ns) and **C1-o** (576 ns) showing significantly longer lifetimes compared to **C2-m** (326 ns) and **C4-ref** (305 ns, Table 3).

### Temperature-Dependent Luminescence Spectra and Lifetimes

To further elucidate the nature of the emitting states and potential thermally activated processes determining the quantum yield and lifetimes, the temperature dependence of the luminescence between 290 K and 5 K was investigated. To cover 290–5 K without artifacts from solvent phase transitions or viscosity changes, the measurements were performed in a rigid KBr matrix. This also keeps conditions consistent with the step-scan FTIR experiments discussed below. At 290 K, the complexes revealed a broad emission band with maxima between 557 nm and 576 nm (Figure 5), depending on the methoxy substitution pattern. Compared to the unsubstituted reference **C4-ref**, the *ortho*- and *para*-substituted compounds **C1-o** and **C3-p** feature slightly blue-shifted emission (7 nm and 15 nm, respectively). In contrast, the *meta*-substituted complex **C2-m** is slightly red-shifted by 4 nm. These trends follow the observations in acetonitrile (see above). This blue shift in the KBr matrix likely stems from the limited structural flexibility of the complexes in the solid-state complexes and reduced excited-state geometric relaxation (rigidochromism).^54,55^

**Figure 5:**
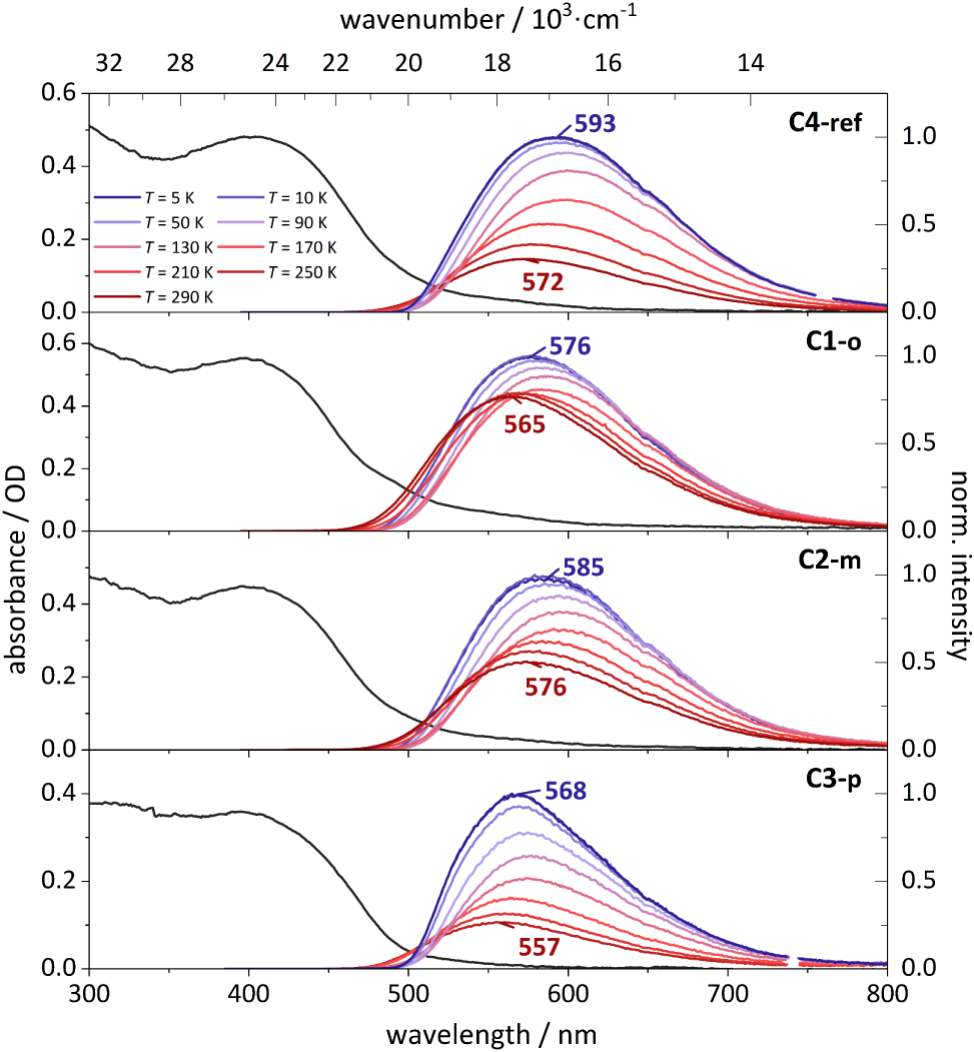
Room-temperature absorption spectra (black solid line) obtained from diffuse reflectance measurements of the samples as powder as well as diluted in KBr and temperature-dependent photoluminescence spectra of **C4-ref** (top), **C1-o** (second), **C2-m** (third), and **C3-p** (bottom) measured in a KBr matrix. The colors of the emission spectra correspond to the different temperatures, which are given in the inset at the top left. The excitation wavelengths are depicted in the Supporting Information (Figure S33).

This could induce a distortion of the excited state geometry with a concomitant increase in energy, shifting the emission to shorter wavelengths.^56,57^

Upon decreasing the temperature to 170 K, a red shift of the emission band is observed for all complexes (Figure 5). This is accompanied by an increase in emission intensity, except for **C1-o**, showing a nearly constant emission intensity. Between 130 K and 5 K, the emission maxima blue-shift again and the emission intensity increases for all complexes. Nevertheless, the emission maxima at 5 K are red-shifted compared to 290 K.

For instance, **C4-ref** has an emission feature at 593 nm at 5 K and at 572 nm at 290 K, corresponding to a shift by 21 nm (76.8 meV). Complex **C2-m** revealed the smallest red-shift of 9 nm (33.1 meV). Across the series, all methoxy-substituted complexes are blue-shifted at 5 K relative to the unsubstituted species **C4-ref**. The observed relative spectral positions originate from a complex interplay of electronic and steric changes upon substitution of the ligand backbone as discussed above. The temperature-dependent changes can be related to a superposition of thermally activated delayed fluorescence (TADF) and reduced geometry relaxation with decreasing temperature (rigidochromism).^54,55^

The red shift upon initial cooling is consistent with an increased contribution of a lower-energy triplet manifold. At the lowest temperatures, suppressed geometric relaxation dominates, causing the subsequent blue shift. At higher temperatures, thermally activated reverse intersystem crossing (RISC) repopulates S_1_, in line with TADF commonly observed for heteroleptic Cu­(I) complexes.^56,58–61^

To support the understanding of the luminescence properties with decreasing temperatures and to further investigate TADF properties, emission lifetimes were measured by time-correlated single photon counting and fitted using the sum of three exponential functions and the amplitude-averaged lifetimes were calculated (SI, Chapter 1 and Chapter 9 for details).

The emission lifetimes of all complexes at 290 K are found to be in the microsecond regime and decrease significantly upon the addition of methoxy groups. Compared to **C4-ref,** with an excited state lifetime of 20.91 μs at 290 K, the lifetime of **C1-o** (8.70 μs) and **C2-m** (7.89 μs) is reduced (Figure 6). With decreasing temperature, the emission lifetimes are significantly prolonged for all complexes, ranging from 303 μs for **C4-ref** to 105 μs for **C1-o**, and the trend upon changing the substitution pattern is similar to higher temperatures. Overall, a nonlinear correlation of lifetimes and temperature is observed, in particular for **C2-m**, which is a strong indication for TADF.^62^ Thus, the lifetimes were fitted assuming thermal equilibrium of the triplet state T_1_ and the singlet state S_1_ (SI, Chapter 1 for details) and revealed energy gaps $\Delta E_{\mathrm{ST}}$ between both states ranging from 702 cm^–1^ for **C1-o** up to 737 cm^1^ for **C3-p** being in the typical range for related Cu­(I)-based TADF complexes.^59,63,64^

**Figure 6:**
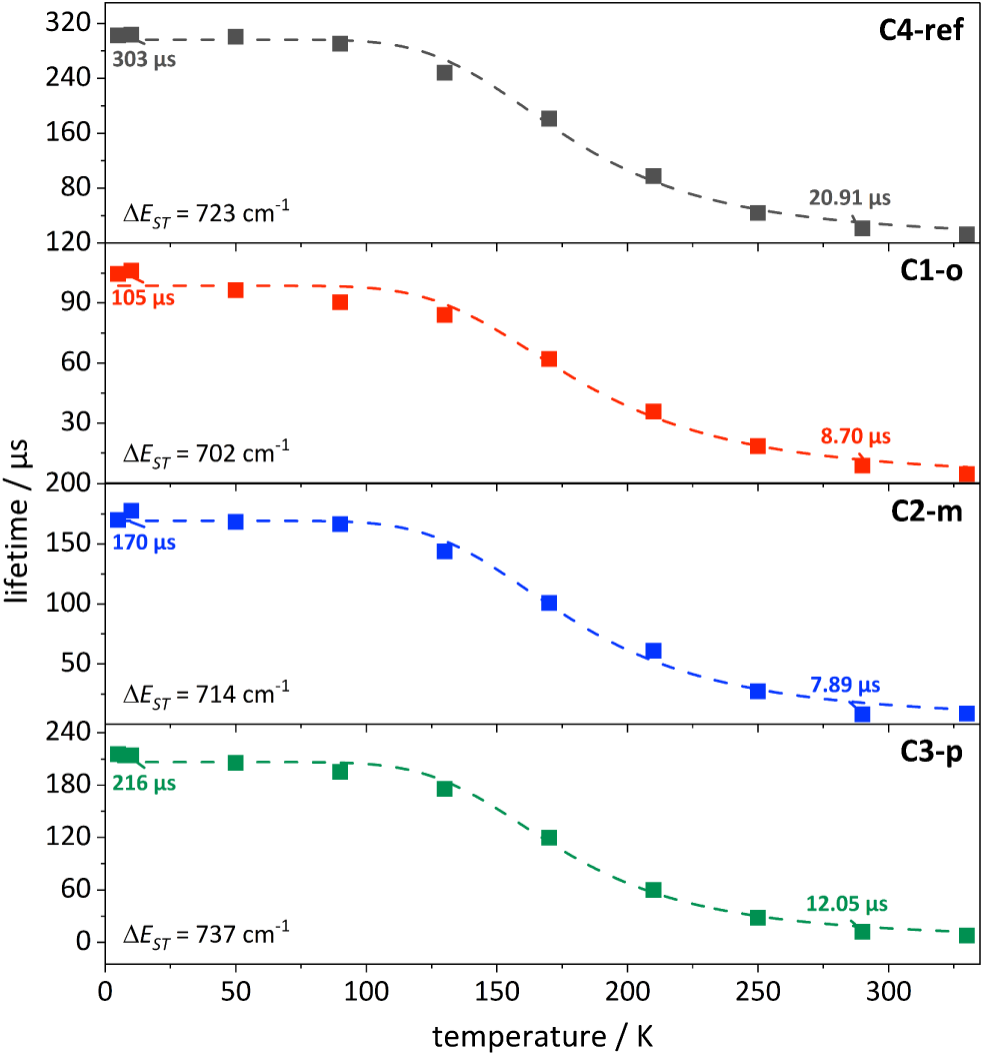
Temperature-dependent emission lifetimes of **C4-ref** (black, top), **C1-o** (red, second), **C2-m** (blue, third) and **C3-p** (green, bottom) measured in KBr matrix with an excitation at 390 nm. The dashed line represents the fit of the experimental data according to eq. (1.5, SI Chapter 1).

Taken together, the temperature-dependent spectral shifts and lifetime trends indicate that the substitution pattern modulates both singlet–triplet energy gaps $\left({\Delta E}_{\mathrm{ST}}\right)$ and nonradiative deactivation pathways. To assess the practical implications for applications, we next compare the room-temperature quantum yields (Φ_em_) and the S_1_ → S_0_ decay rates. At 290 K, although **C1-o** exhibits slightly longer excited state lifetimes than **C2-m** (8.70 μs vs. 7.89 μs), the smaller ${\Delta E}_{\mathrm{ST}}$ and higher Φ_em_ render it the more promising candidate for future applications. In contrast, the *para*-substituted complex **C3-p** reveals similar quantum yields to **C1-o**, but an increased $\Delta E_{\mathrm{ST}}$ and slower S_1_ → S_0_ decay (τ = 12.05 μs). The reference **C4-ref** facilitates the least beneficial TADF properties considering the low quantum yields and long emission lifetime. To further elucidate the interplay of steric and electronic effects on the excited states geometry, we next employed time-resolved, structure-sensitive step-scan FTIR spectroscopy at cryogenic temperatures.

### Time-Resolved Step-Scan FTIR Spectroscopy at Cryogenic Temperatures

Common solvents (*e.g*., MeCN or DCM) exhibit intense mid-IR bands that would mask the excited-state features, but using a KBr matrix avoids solvent interferences and keeps conditions consistent across the applied techniques. Therefore, the steady-state FTIR spectra of **C4-ref**, **C1-o**, **C2-m** and **C3-p** were recorded in KBr at 18–28 K from 1180 cm^–1^ to 1750 cm^1^, revealing several intense bands (Figure 7). To support the assignment of the spectral features the ground state (S_0_) geometry of each complex was optimized (computational details in SI), and frequency calculations were carried out. The FTIR spectrum of **C4-ref** reveals intense vibrations at 1227 cm^–1^, 1410 cm^–1^, 1436 cm^–1^ and 1482 cm^–1^ that are mainly localized on the xantphos ligand with only minor contributions from the bathocuproine (detailed assignment, Table S16), consistent with previously reported FTIR results.^32^ The bands above 1500 cm^1^ are predominantly associated with the bathocuproine ligand. Thus, the chosen spectral window is suitable to monitor both changes within the bathocuproine framework, including potential influence of the methoxy substituents as well as possible changes in the xantphos ligand. Only subtle spectral differences are observed upon variation of the bathocuproine backbone, indicating that the S_0_ geometry and electronic structure are nearly independent of the substitution pattern.

**Figure 7:**
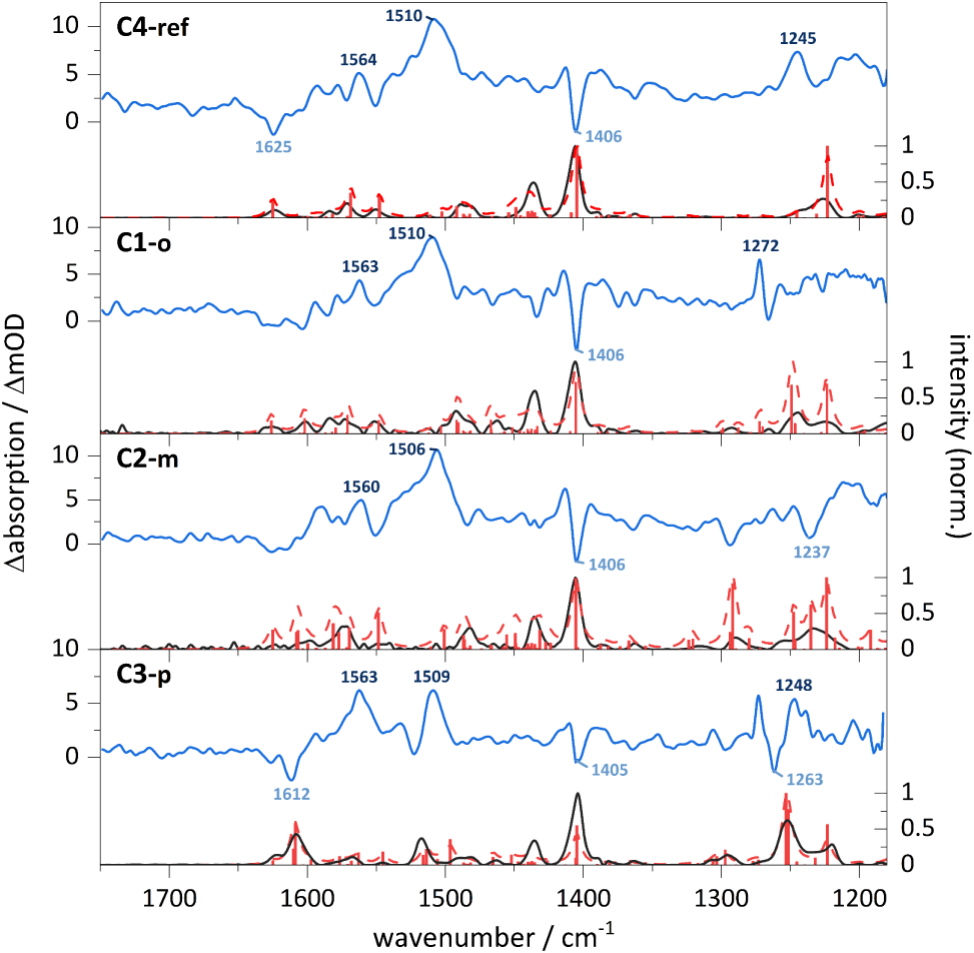
Cryogenic step-scan FTIR spectra at 0–1.0 μs after laser excitation (blue line) of **C4-ref** (top), **C1-o** (second), **C2-m** (third), and **C3-p** (bottom) measured in KBr matrix with excitation at 355 nm. Ground state bleach bands are labeled in light blue and excited-state vibrations in dark blue. The ground-state FTIR spectra at cryogenic temperatures are depicted as solid black line and the calculated S_0_ frequencies as red sticks and dashed folded spectra. Calculations: DFT/B3LYP-D3­(BJ)/def2-TZVP/COSMO, scaled by 0.975 with Gaussian profile, fwhm = 8 cm^–1^.

The complexes are sufficiently stable for the cryogenic, time-resolved step-scan measurements (Figure S39), and thus, differential step-scan FTIR spectra (Figure 7, blue line) were recorded with 355 nm excitation (1.3–1.6 mJ per pulse), and the signals from 0 to 1000 ns after excitation were averaged. The positive features correspond to excited-state vibrations, whereas the negative bands are caused by ground-state bleach. For all complexes, intense excited-state features are observed, while the ground state bleach is comparatively weak. Upon addition of 0.8 – 1.4% of the ground state spectrum to the differential step-scan FTIR spectra, the solely excited-state vibrational spectra were extracted. The excited-state spectrum of **C4-ref** (Figure 8, top) shows intense features at 1564 cm^–1^, 1510 cm^–1^, 1411 cm^–1^ and 1245 cm^–1^, in agreement with previous reports.^32^ These excited-state vibrational features match the calculated T_1_ spectrum with spin density mainly located on the phenanthroline unit (Table 5). Upon introduction of methoxy substituents only subtle changes arise, mainly below 1300 cm^–1^, caused by changes in the methoxy *v*_*CO*_ vibrational modes. Additional features between 1550 and 1600 cm^– 1^ are observed, which are represented in the calculated T_1_ spectrum with reduced oscillator strength for all complexes. The underlying weak vibrational modes between 1564 cm^–1^ to 1574 cm^–1^ can be assigned to rocking motions solely localized at the backbone phenyl rings. The underestimation of the oscillator strength in the calculated T_1_ spectra may indicate contributions from additional excited-states with higher spin density located at the backbone phenyls. A geometry in which the phenyl group is rotated into coplanarity with the phenanthroline unit would increase the orbital overlap, and thus, delocalize the spin further.

**Figure 8:**
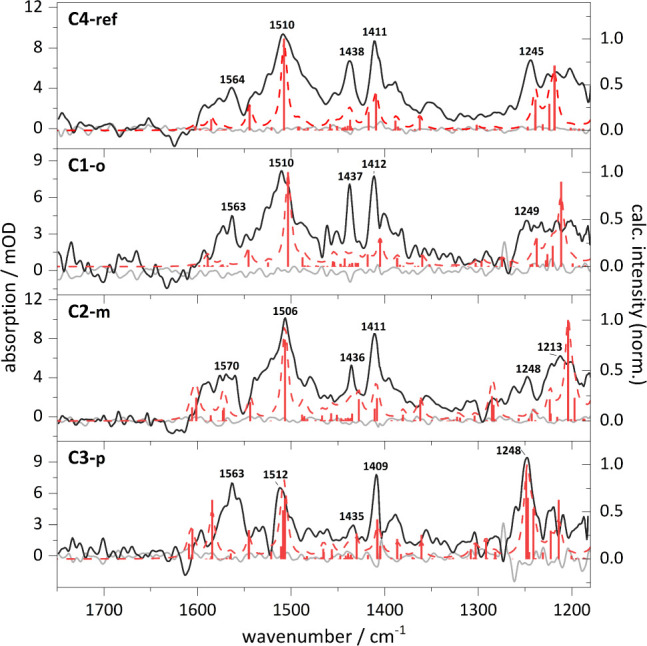
Excited-state FTIR spectra of **C4-ref** (top), **C1-o** (second from top), **C2-m** (third from top) and **C3-p** (bottom) obtained from the step-scan FTIR spectra at 0–1.0 μs after excitation and with addition of 0.8–1.4% ground-state intensity. The calculated T_1_ frequencies are shown as red sticks and dashed folded spectra. Calculations: DFT/B3LYP-D3­(BJ)/def2-TZVP/COSMO, scaled by 0.975 with Gaussian profile, fwhm = 8 cm^–1^.

To also extract the excited-state lifetimes, the decays of the most prominent positive and negative features were chosen and fitted using a convolution of a Gaussian pulse with a three-exponential decay as fit model (SI Chapter 1, Figures S49–S52 and Table S24). All complexes exhibit microsecond lifetimes, consistent with the luminescence lifetimes. Notably, the *meta*-substituted **C2-m** displays the longest excited-state lifetime in the step-scan FTIR data (*τ*_*av*_ = 96.6 μs), in contrast to the emission lifetime measurements. This discrepancy suggests a larger contribution of a possible nonemissive (“dark”) excited-state in **C2-m**, which may be relevant for its application in photocatalysis. Taken together, the substitution-dependent changes in Δ*E*_ST_, the population of triplet states, and indications of a nonemissive (“dark”) manifold for **C2-m** suggest distinct consequences for photochemical reactivity. To test these implications, all Cu­(I) complexes were benchmarked in three representative light-driven transformations, covering both energy transfer (singlet oxygen generation) and electron-transfer photocatalysis (hydrogen evolution and reductive dehalogenation).

### Singlet Oxygen Generation

Singlet oxygen (^1^O_2_) is a reactive, high-energy form of molecular oxygen that plays a key role in diverse applications ranging from photodynamic therapy (PDT) to selective oxidation reactions in synthetic chemistry.^65–69^ Its generation typically occurs via energy transfer from the triplet excited-state of a photosensitizer to ground-state molecular oxygen (^3^O_2_).^70^ The resulting ^1^O_2_ species exhibits a characteristic near-infrared (NIR) phosphorescence with an emission maximum at approximately ≈ 1270 nm, corresponding to an energy gap of 94.3 kJ mol^– 1^.^66,69^ To evaluate the photosensitizing capabilities of the present Cu­(I) complexes and to analyze how the ΔE_ST_, the lifetime and yield of the triplet states influence the energy transfer efficiency, singlet oxygen quantum yields $\left(\phi_{1_{O_{2}}}\right)$ in dichloromethane were determined by NIR emission spectroscopy using phenalenone as an established reference.^71,72^ Measured under identical conditions, all four complexes exhibit similar, moderate singlet-oxygen quantum yields $\phi_{1_{O_{2}}}$ in the range from 40% for **C2-m** to 49% for **C4-ref** (Table 3). In this case, it is difficult to discuss the small differences clearly, as they lie within the experimental deviation of the NIR method. **C1-o** and **C3-p** now show comparable $\phi_{1_{O_{2}}}$ of 45%, consistent with their similar Φ_em_ (2.1%) and τ_em_ (≈0.6 μs) in solution. However, across the full series variations in $\phi_{1_{O_{2}}}$ from ≈ 40 to 49% are not fully captured by Φ_em_ and τ_em_ alone, motivating the analysis of spin-density localization presented below.

As a practical benchmark, the photooxidation of diphenylfuran (DPF) to *cis*-dibenzoylethylene (DBE) was followed over 90 min with a photosensitizer-to-substrate ratio of 1:20. Blank experiments (dark or without photosensitizer) showed no/only negligible conversion, confirming the light-driven nature. Time courses were fitted with pseudo-first-order kinetics, yielding similar rate constants within the same order of magnitude (*k*_c_ = 2.04·10^–4^ s^–1^ for **C1-o**, *k*_c_ = 2.10·10^–4^ s^–1^ for **C2-m**; *cf*. SI Table S26). The magnitudes and trends are consistent with our earlier study on related Cu­(I) photosensitizers.^23^

### Hydrogen Evolution Reaction

The photocatalytic hydrogen evolution performance of the two new Cu­(I) complexes **C1-o** and **C2-m** was evaluated in a well-established three-component system comprising Fe_3_(CO)_12_ as the water reduction catalyst (WRC), triethylamine (TEA) as sacrificial electron donor (SD), and the Cu­(I) complex as photosensitizer.^17,37,73^ Reactions were conducted in a THF/TEA/H_2_O mixture (4:3:1, v/v/v) under illumination with a 150 W xenon arc lamp. To promote reductive quenching of the excited photosensitizer, a large excess of TEA (≈ 7600 equiv.) was used, minimizing competitive oxidative quenching by the WRC.^37,49,73^ The complexes **C1-o** and **C2-m** as well as the references **C3-p** ^24^ and **C4-ref** ^37^ were catalytically active and enabled visible-light-driven H_2_ evolution under the applied conditions (Figure 9). Among them, the *para*-substituted complex **C3-p** showed both the highest initial hydrogen evolution rate and the highest overall turnover number (TON ≈ 590 after 20 h).

**Figure 9:**
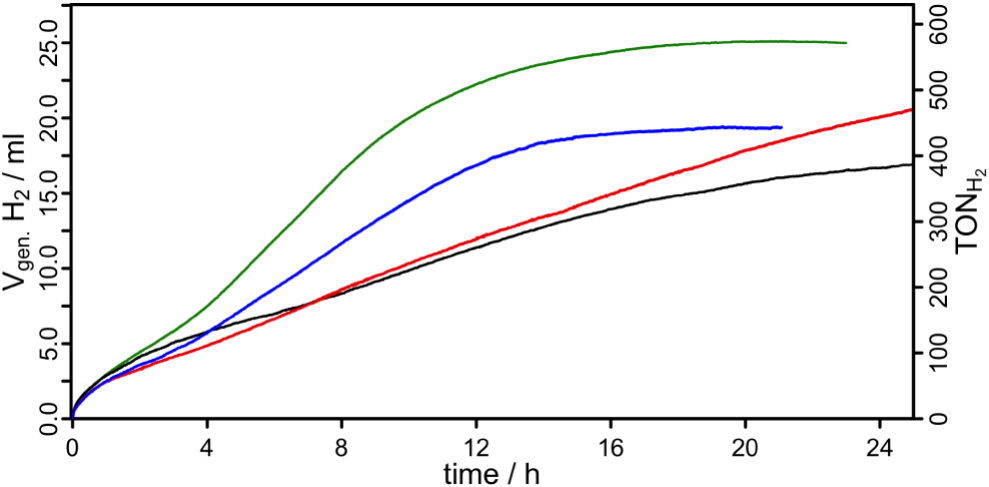
Photocatalytic hydrogen evolution curves for the complexes **C1-o** (red), **C2-m** (blue), **C3-p** (green), and **C4-ref** (black) in the presence of Fe_3_(CO)_12_ as WRC and TEA as SD. Detailed reaction conditions are placed in the SI (Chapter 1).

In contrast, the *ortho*-substituted derivative **C1-o** maintained catalytic activity over an extended period and reached a TON ≈ 480 after 24 h and ≈ 530 after 36 h, outperforming the *meta*-substituted **C2-m** (TON ≈ 450) and the unsubstituted reference **C4-ref** (TON ≈ 397). It is noteworthy that **C1-o** requires a substantially longer time (36 h, Figure S62) to reach its plateau, whereas **C2-m**, **C3-p** and **C4-ref** reach theirs within 20–24 h.

In general, these observations correlate well with the photophysical and electrochemical properties of the complexes. **C3-p** features the longest excited-state lifetime (τ_em_ = 628 ns) and highest emission quantum yield (Φ_em_ = 2.1%), facilitating efficient excited-state electron transfer and high catalytic activity.^24^ **C1-o** also shows a relatively long-lived excited state (τ_em_ = 576 ns), which likely contributes to its sustained activity. In contrast, **C2-m** (τ_em_ = 326 ns) and **C4-ref** (τ_em_ = 305 ns) exhibit significantly shorter excited-state lifetimes and lower hydrogen evolution. Additionally, the excited-state reduction potentials $\left(E_{1/2}^{* red}\right)$ influence the thermodynamic driving force for electron transfer to the WRC. **C3-p** ( $E_{1/2}^{* red}$ = 0.48 V) and **C1-o** (0.46 V) exhibit the least oxidizing excited states within the series, consistent with their superior performance. **C2-m**, with the most anodically shifted excited-state potential (0.50 V), shows reduced catalytic activity, further supporting the critical role of both photophysical and electrochemical parameters in photocatalytic H_2_ evolution. To identify the operative quenching pathway under the H_2_ evolution conditions, Stern–Volmer analyses were performed in THF with TEA for two representative complexes (**C2-m** and **C3-p**). Both systems show a concentration-dependent decrease of the excited-state lifetime with linear Stern-Volmer behavior. The extracted quenching constants are of similar magnitude (*k*_q_ = 2.80 × 10^6^ M^–1^·s^–1^ for **C2-m**, *k*_q_ = 1.83 × 10^6^ M^–1^·s^–1^ for **C3-p**; for fits and further information see SI Chapter 14).^24^ These data support a reductive quenching entry step for H_2_ evolution in THF/TEA. This result is also consistent with earlier observations on related Cu­(I) complexes, where 1,3-dimethyl-2-phenylbenzimidazoline (BIH) acted as reductive quencher for **C3-p** in MeCN.^23^

### Photocatalytic Reductive Dehalogenation Reactions

To complement the hydrogen-evolution study and assess the broader catalytic scope, we investigated the visible-light-driven reductive dehalogenation of two aryl bromides, 4-bromo-benzophenone (E_1_) and 2-bromo-acetanilide (E_2_), using **C1-o, C2-m, C3-p, and C4-ref**. Reactions were carried out in deaerated acetonitrile with a sacrificial donor mixture (1 equiv. BIH and 10 equiv. TEA) under blue LED irradiation (λ = 460 nm, *cf*. SI Chapter 1). Control experiments confirmed that light, photocatalyst, and sacrificial donors are all required. UV/vis monitoring indicated good photostability of the Cu­(I) complexes under the applied conditions (*cf*. Figure S66). All four complexes are catalytically active (Table 4).

**Table 4:**
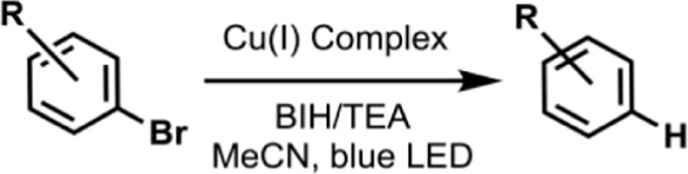

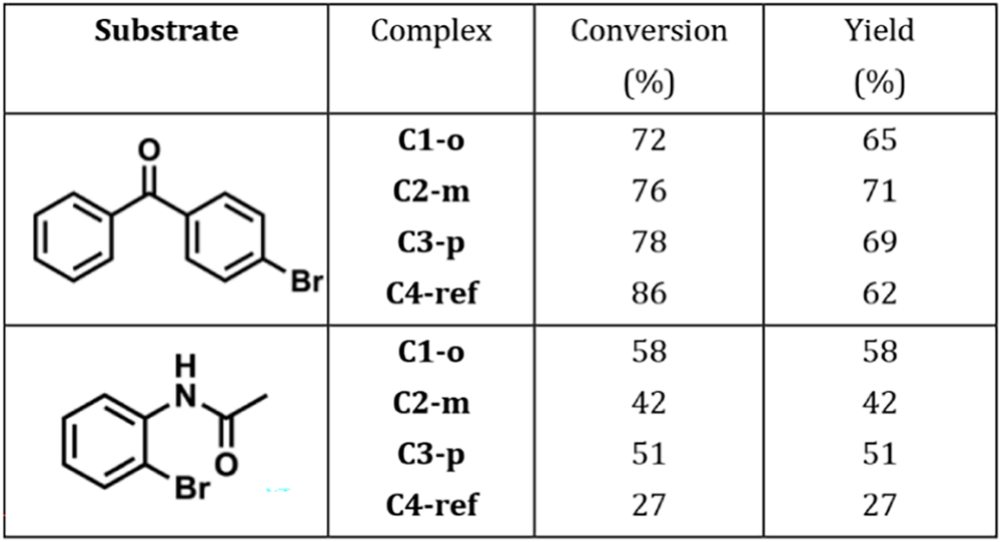
Summary of the Photocatalytic Reductive Dehalogenation of 4-Bromobenzophenone (E_1_) and 2-Bromo-Acetanilide (E_2_) Using the Complexes **C1-o**, **C2-m**, C3-p and **C4-ref** ^a^

a Conditions: Substrate = 0.05 mmol, Catalyst = 1 mol% in deaerated acetonitrile, Blue LED (460 nm, SI Chapter 14).

For the faster substrate E_1_ (15 min), the trend in yields is **C2-m** (71%) > **C3-p** (69%) > **C1-o** (65%) > **C4-ref** (62%), while the conversions follow **C4-ref** (86%) > **C3-p** (78%) > **C2-m** (76%) > **C1-o** (72%). Thus, despite the highest conversion, **C4-ref** affords the lowest yield, whereas the methoxy-substituted systems deliver higher yields under identical conditions. The yields of the dehalogenation of E_1_ correlates with the excited-state reduction potentials $E_{1/2}^{* red}$ (**C2-m** 0.50 V > **C3-p** 0.48 V > **C1-o** 0.46 V; **C4-ref** 0.49 V) and is further supported by the emission lifetimes τ_em_ (**C3-p** 628 ns, **C1-o** 576 ns, **C2-m** 326 ns, **C4-ref** 305 ns). This implies that the more reducing and/or longer-lived excited states favor rapid productive quenching within the reaction time.

For E_2_ (within 5 h), conversions and yields agree within the experimental error and follow **C1-o** (58%) > **C3-p** (51%) > **C2-m** (42%) > **C4-ref** (27%). Here, the ranking matches the ground-state reducing power of the reduced photosensitizer, reflected by the first reduction potentials $E_{1/2}^{\mathrm{red}}$ (more negative = stronger): **C1-o** −2.11 V < **C3-p** −2.08 V < **C4-ref** −2.04 V < **C2-m** −2.03 V. For this more demanding substrate E_2_, electron transfer from the reduced PS^–^ to the aryl halide appears more decisive than excited-state access, favoring **C1-o**. These correlations provide practical guidance without implying a definitive mechanism.

### Influence of Excited-State Geometry and Location on the Sensitizing Abilities and Catalytic Performance

Next, the influence of excited-state geometry and spin-density distribution on the sensitizing abilities and catalytic performance was analyzed across the series (Table 5). For all complexes, the lowest excited triplet state T_1_ spin density is located mainly at the phenanthroline moiety of the bathocuproine ligand with contribution of the Cu (I) center (Figure 10), which is commonly known for heteroleptic Cu­(I) complexes.^12,48^ The electronic contribution of the Cu­(I) center to the excited-state can promote flattening of the distorted-tetrahedral ground-state geometry of the complexes.^74,75^ Indeed, distortion of the T_1_ state due to flattening of 10.4° has been reported for **C4-ref** ^32^ and is also evident for our calculated structures (Table S2). Within the present series, **C1-o** shows the largest flattening distortion of 9.0° of the lowest triplet T_1_ with respect to the ground state geometry, whereas **C3-p** exhibits the least flattening of 6.4°. The complex **C2-m** facilitates intermediate flattening of 7.2°. The enhanced excited state distortion of **C1-o** could contribute to its slightly shorter emission lifetime in solution compared to **C3-p**, in line with step-scan FTIR and emission data. Moreover, the larger backbone phenyl torsion angles observed for **C1-o** are consistent with the higher metal center spin density (Table 5).

**Table 5:** Spin Densities of the T_1_ State Calculated with Mulliken Population Analysis (SI Chapter 1) for Different Moieties of the Cu­(I) Complex

Moiety	**C1-o**	**C2-m**	**C3-p**	**C4-ref**
Cu(I) center	0.4825	0.4728	0.4736	0.4793
phenanthroline unit	1.1802	1.1806	1.1741	1.1579
backbone phenyl	0.0236	0.0400	0.0417	0.0415
xantphos ligand	0.3137	0.3066	0.3106	0.3213

**Figure 10:**
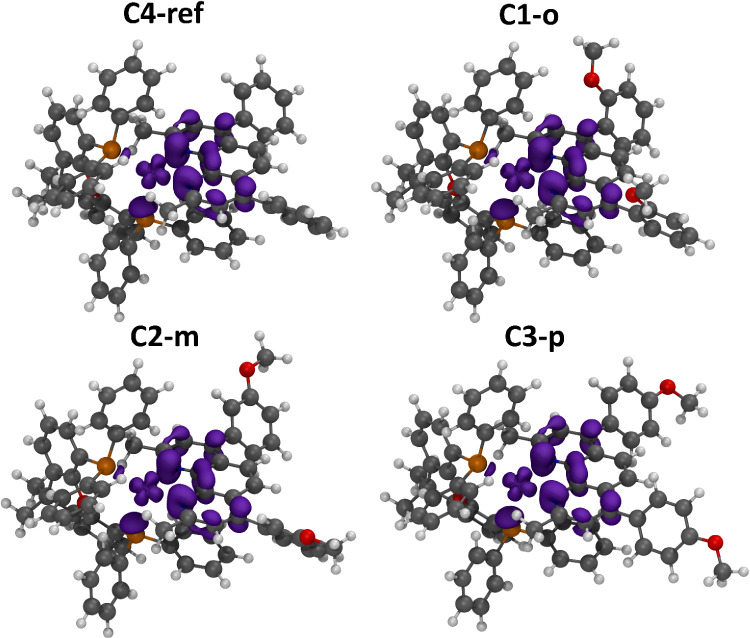
T_1_ spin density isosurfaces of **C4-ref** (top left), **C1-o** (top right), **C2-m** (bottom left) and **C3-p** (bottom right) visualized using an isovalue of 0.005.

As a consequence, the excited-state reduction potential is the least oxidizing for **C1-o** ( $E_{1/2}^{* red}$ = 0.46 V), as shown above (Table 2).

Thus, substitution patterns that increase the spin density at the metal center appear beneficial for electron transfer reactions, consistent with the trends in H_2_ evolution and reductive dehalogenation of E_2_. By contrast, **C2-m** shows the least spin density at the copper center and xantphos moiety, but a higher population at the phenanthroline ligand and the phenyl backbone. The more ligand localized character could lead to a stabilization of the excited-state, prolonging the excited-state lifetime and the activity for reactions governed by energy transfer.

## Conclusion

In this work, a series of heteroleptic Cu­(I) photosensitizers bearing methoxy-substituted phenyl groups in *ortho*-, *meta*-, and *para*-positions were investigated systematically. By combining synthetic, structural, photophysical, electrochemical, and photocatalytic studies, the influence of the substitution pattern on key properties relevant for photosensitizer design was elucidated.

Single-crystal X-ray diffraction and DFT calculations confirmed the expected distorted tetrahedral coordination geometry around the Cu­(I) center, while revealing subtle but systematic effects of the substitution pattern on ligand torsion angles and the overall conformation. Electrochemical studies demonstrated that the *ortho*-methoxy group causes the strongest electron-donating influence, leading to cathodically shifted reduction potentials and an enhanced excited-state oxidizing power compared to the *meta*- and *para*-derivatives. Photophysical investigations in solution revealed that both *ortho*- and *para*-substitution significantly enhance absorptivity, emission quantum yields, and excited-state lifetimes, while *meta*-substitution has only a minor influence. This highlights the crucial role of substituent position for tuning light-harvesting efficiency and excited-state properties. Under identical measurement conditions, singlet oxygen quantum yields are similar and moderate across the series (Φ^1^O_2_) ≈ 40–49%), with **C1-o** and **C3-p** giving comparable values. Complementary temperature-dependent luminescence and step-scan FTIR measurements indicate that the substitution pattern also modulates excited-state relaxation pathways. In particular, the *meta*-isomer shows characteristics that suggest access to a nonemissive (“dark”) manifold, rationalizing its reduced photophysical performance.

The catalytic results directly link the substitution-controlled photophysical and electrochemical parameters to the observed reactivity. In photocatalytic hydrogen evolution, the *para*-substituted complex exhibits the highest initial rate and the highest TON, which is in line with the longest excited-state lifetimes and favorable excited-state reduction potentials. Stern-Volmer quenching also establishes a reductive entry step under the applied H_2_ evolution conditions (THF/TEA). In photocatalytic reductive dehalogenation of two representative substrates, the catalyst ranking becomes substrate-dependent: for the faster transformation (E_1_), the outcomes correlate with excited-state reduction potential $(E_{1/2}^{* red}$ ) and lifetime, whereas for the more demanding substrate (E_2_) the trend follows the ground state reducing power $(E_{1/2}^{\mathrm{red}}$ ) of the reduced Cu­(I) complex. Thus, there is no single complex that is universally optimal for all photocatalytic transformations, and the specific reaction determines which substitution pattern works best.

Taken together, these results provide detailed insight into the interplay between substitution pattern, electronic structure, and photophysical behavior in Cu­(I) photosensitizers. Although the previously reported *para*-substituted complex **C3-p** remains the most efficient system within this series, the new data clearly demonstrate how even subtle positional variations of methoxy groups can be used to systematically tune key properties such as absorption, excited-state lifetimes, redox behavior, and energy-transfer capability — thereby guiding catalyst choice for a given transformation. Looking ahead, the rational design of next-generation Cu­(I) photosensitizers for photocatalytic applications should be further advanced by combining donors in *ortho*/*para*-positions with steric control of aryl torsion, expanding the substrate scope to map the excited- vs. ground-state boundary, and correlating time-resolved spectroscopy with catalytic metrics.

## Experimental Section

A comprehensive description of all materials and methods — including experimental and computational protocols, synthetic procedures, NMR data, DFT calculations, electrochemical measurements, absorption and emission as well as time-dependent and temperature-dependent emission spectroscopy in solid and liquid state, quenching studies, and photocatalytic experiments (singlet oxygen generation, hydrogen evolution reaction, reductive dehalogenation reaction) — is provided in the Supporting Information.

## Supplementary Material



## Notes

Experimental, synthetic, computational, spectroscopic, and electrochemical details, as well as information about photocatalysis. Crystallographic data for complex **C2-m** has been deposited at the joint Cambridge Crystallographic Data Centre (CCDC) and Fachinformationszentrum Karlsruhe under CCDC 2482019 and can be obtained from www.ccdc.cam.ac.uk/structures/or by emailing data_request@ccdc.cam.ac.uk (PDF)
