# Supplementary material for: Positional Tuning of Photophysics and Catalysis in Methoxy-Substituted Heteroleptic Copper(I) Complexes
Source: Inorg Chem. 2025 Dec 2;65(10):5306–18. doi: 10.1021/acs.inorgchem.5c04739 (PMC12997163; doi:10.1021/acs.inorgchem.5c04739)
Supplement: Supplementary file 1 [file ic5c04739_si_001.pdf]

Supporting Information

Positional Tuning of Photophysics and Catalysis in Methoxy-Substituted Heteroleptic Copper(I) Complexes

Kurt J. Haseloff,[1,†] Katharina Rediger,[2,†] Mohammad D. Mandourah,[1] Max Wolf,[1] Christian Kleeberg,[3] Stefanie Tschierlei,[1,] Maria Wächtler,[4,*] and Michael Karnahl [1,*]*

[1] Department of Energy Conversion, Institute of Physical and Theoretical Chemistry, Technische Universität Braunschweig, Rebenring 31, 38106 Braunschweig, Germany.
[2] Department of Chemistry, Rheinland-Pfälzische Technische Universität Kaiserslautern-Landau, Erwin-Schrödinger-Straße 52, 67663 Kaiserslautern, Germany.
[3] Institute of Inorganic and Analytical Chemistry, Technische Universität Braunschweig, Hagenring 30, 38106 Braunschweig, Germany.
[4] Institute of Physical Chemistry, Christian-Albrechts-Universität zu Kiel, May-Eyth-Straße 1, 24118 Kiel, Germany.

† These authors have contributed equally to this work and share first authorship.
*Correspondence:

Prof. Dr. Stefanie Tschierlei	s.tschierlei@tu-bs.de
Prof. Dr. Maria Wächtler	waechtler@phc.uni-kiel.de
PD Dr. Michael Karnahl	michael.karnahl@tu-bs.de

1 Experimental Details	2
2 Synthetic Details	7
3 NMR	10
4 MS	17
5 Crystallographic Data	21
6 Density Functional Theory (DFT)	22
7 UV/vis Spectroscopy	43
8 Steady-state Emission	44
9 Time-resolved Emission	48
10 Step-scan FTIR Spectroscopy	54
11 Electrochemical Data	66
12 Excited State Reduction Potentials	67
13 Photostability	68
14 Photocatalysis	69
15 References	76

1 Experimental Details

NMR Spectroscopy. Nuclear magnetic resonance (NMR) spectra, including ^{1}H NMR, $^{13}C\{^{1}H\}$ NMR, and $^{31}P\{^{1}H\}$ NMR, were acquired using spectrometers of the *Bruker Avance* Series (400 MHz, 500 MHz or 600 MHz) at 298 K. The frequency is specified in the respective NMR data. The measurements were performed by the Institute of Inorganic and Analytical Chemistry at the Technische Universität Braunschweig. Measurements were conducted using Wilmad 528-PP NMR tubes and the solvent for each sample is specified in the respective NMR data. The acquired NMR spectra were subsequently processed using TopSpin software (version 4.3.0). The chemical shifts δ are reported in ppm relative to tetramethylsilane (TMS), and all NMR spectra were referenced to the TMS signal. Coupling constants (J) are presented as absolute values in hertz (Hz). The NMR signal splitting was described using standard notations: '*s*' for singlet, '*d*' for doublet, '*t*' for triplet, '*dd*' for doublet of doublets, '*td*' for triplet of doublets, and '*m*' for multiplet. Quintet splitting was identified as 'quintet'.

Mass Spectrometry. Mass spectrometric (MS) analyses were conducted by the analytical service of the Institute of Organic Chemistry, Technische Universität Braunschweig, using an LTQ-Orbitrap Velos mass analyzer manufactured by ThermoFisher Scientific. High-resolution mass spectra were obtained *via* electrospray ionization (ESI). The samples were dissolved in methanol and spiked with 0.1 mg/mL tetradecyltrimethylammonium bromide. Mass spectrometry values are reported as mass-to-charge ratios (*m/z*).

Elemental Analysis (EA). The elemental analyses were performed by the Institute of Inorganic and Analytical Chemistry at the Technische Universität Braunschweig, using a varioMICRO Cube apparatus from *Elementar*. Under the CHN configuration, only carbon (C), hydrogen (H), and nitrogen (N) were measured. Helium served as the inert gas.

X-ray Diffraction Studies. A single crystal was mounted on top of a Hampton loop using inert perfluoroether oil and placed in the cold nitrogen gas stream on the diffractometer.[1] The diffraction data were collected on a Rigaku Oxford Diffraction Synergy-S instrument using mirror-focused CuKα radiation from a Rigaku Photonjet microfocus source. The reflections were indexed, integrated and corrected for absorption as implemented in the CrysAlisPro software package.[2] The structures were solved employing the program SHELXT and refined anisotropically for all non-hydrogen atoms by full-matrix least squares on all F2 using SHELXL software.[3,4,5] During refinement and analysis of the crystallographic data the programs Mercury, PLATON and OLEX2 were used.[6,7,8] Further details are found in Table S1 and the supplementary crystallographic data available under CCDC 2482019. X-ray structures were depicted using Diamond (Crystal Impact) with all hydrogen atoms omitted for clarity and atoms drawn as thermal ellipsoids at a 50% probability level.[3,9]

(TD-)DFT Calculations. The ground state S_0 and excited triplet state T_1 geometries of the complexes were optimized using the Berny algorithm of Gaussian 16,[10] with the energies and gradients computed by TURBOMOLE (v.7.8).[11] All optimizations were performed with the DFT functional B3LYP with additional dispersion correction (D3-BJ, as implemented in TURBOMOLE) and the def2-TZVP basis set. For acceleration, the resolution of identity (RI) in combination with the multipole accelerated RI-J with a def2-TZVP auxiliary basis set was used. To also consider solvation effects, the conductor-like screening model (COSMO) for KBr with a permittivity of 4.9 and refractive index of 1.3441 was applied. All optimized geometries were checked for negative frequencies and assumed to be energy minimum if no negative

frequencies were present. The extracted IR spectra were plotted using Gaussian shape line and a fwhm of 8 cm^{-1}. The partial vibrational spectra were generated using the Multiwfn wavefunction analyzer.[12] The population analysis of the triplet T_1 state and thus spin densities per atom were evaluated using the Mulliken population analysis as implemented in TURBOMOLE (V.7.8). Further, the spin densities of T_1 were visualized using VMD-Visual Molecular Dynamics Version (1.9.3).[13]

Steady-state UV/vis Absorption Spectroscopy. UV/vis absorption spectra were obtained using a JASCO V 770 spectrophotometer. The compounds were dissolved in acetonitrile or methanol (HPLC grade) and the resulting spectra were recorded using a standard 10 mm fluorescence quartz glass cuvette. All spectra were baseline corrected at 800 nm. The solid-state UV/vis absorption spectra were recorded with a JASCO V-780 spectrophotometer equipped with a 150 mm UV/vis integrating sphere. The powder samples were diluted with KBr (stored in a compartment dryer at 90 °C, purchased from MERCK) powder in a ratio of 1:80, ground to a homogeneous mixture, and measured using a powder sample holder. The diffuse reflectance was measured and converted to absorption using the following equation (eq. 1.1).

$$A = -\log\left(\frac{R_{\text{sample}}}{R_{\text{KBr}}}\right) \qquad \text{eq. 1.1}$$

Steady-state Emission Spectroscopy. The emission spectra were recorded using a HORIBA JOBIN-YVON FLUOROMAX PLUS-C emission spectrometer. All samples were measured in dry acetonitrile under inert conditions using a sealed 10 mm fluorescence quartz glass cuvette. Optical densities (OD) were approximately 0.1 at the respective excitation wavelength. The emission quantum yields were calculated using the following equation (eq. 1.2).

$$\Phi_c = \Phi_R \left(\frac{\eta_C^2}{\eta_R^2}\right)\left(\frac{A_R}{A_C}\right)\left(\frac{I_C}{I_R}\right) \qquad \text{eq. 1.2}$$

Time-resolved Emission Spectroscopy. Time-resolved emission spectra were obtained using a streak camera system (HAMAMATSU PHOTONICS C10910-01 Main Unit, accompanied by a M10913 Slow Single Sweep Unit). The signal acquisition process was facilitated by the utilization of an ORCA-FLASH 4.0 V3 camera from HAMAMATSU PHOTONICS. The spectrograph employed in this study is a KYMERA 328I-A from ANDOR, which is directly connected to the streak unit. Excitation was performed using a titanium:sapphire laser (SOLSTICE ACE from SPECTRA PHYSICS) with a repetition rate of 1 kHz and an approximate pulse width of 70 fs. The output was directly coupled to an optical parametric amplifier (LIGHT CONVERSION TOPAS PRIME with NirUVis extension) to set the excitation wavelength to 390 nm. The power at the sample was approximately 320 nW. The center wavelength of the spectrograph is specified for each spectrum. The emission was detected using the single photon counting mode in a time range of 10-50 µs. For all measurements, the input slit of the spectrograph was set to 250 µm, the slit in front of the streak camera to 20 µm, and a 50 grooves/mm grating was utilized. The measurements were obtained with an exposure time of 30 milliseconds and accumulated over 60000 exposures. All samples were prepared under inert conditions, and the optical density was set to 0.1 at 390 nm. The emission lifetime was determined by fitting equation (1.3).

$$f(t) = \sum_{i=1}^{N} A_i \cdot e^{-\frac{t}{\tau_i}} + A_0 \qquad \text{eq. 1.3}$$

Temperature-dependent Luminescence Spectroscopy. The steady-state luminescence spectra of the samples prepared as KBr pellets were measured with a HORIBA JOBIN-YVON FLUOROLOG 3-22T luminescence spectrometer. A xenon lamp (450 W) was used for excitation of the sample, whereas a photomultiplier detector R928P was equipped to detect the photoluminescence. All measured spectra were corrected by the wavelength-dependent sensitivity of the spectrometer. To achieve cryogenic temperatures, the spectrometer was equipped with a closed-cycle helium cryostat COLDEDGE 101 J cryocooler and temperature control with a heating element. The KBr pellets were prepared using 1.0 mg of the sample (**C1-o**, **C2-m**, **C3-p** or **C4-ref**) diluted with 180 mg of KBr (stored in a compartment dryer at 90 °C, purchased from MERCK) and ground and mixed until homogeneity was achieved. The mixture was transferred into an evacuable pellet die (diameter 13 mm) and sintered at a pressure of 10 t using the manual hydraulic press from SPECAC.

Temperature-dependent Luminescence Lifetime Measurements. For the measurement of the luminescence lifetimes of the samples prepared as KBr pellets, a HORIBA JOBIN-YVON DELTAFLEX TCSPC spectrometer was used. The sample was excited using a pulsed SpectraLED with a wavelength of 390 nm and a pulse width of 1 % of the chosen measurement time range, leading to pulse widths ranging from 3.4 µs up to 220 µs. Spectral selection was achieved with a single grating monochromator, and a PPD-850 detector was used for detection of the emitted photons. Additionally, a long-wave pass filter at 496 nm was used to reduce the influence of scattered excitation light. However, the internal response function was recorded using a scattering cuvette, whereas the monochromators were set to the wavelength of the excitation light. The obtained decay curves were analyzed using DecayFit[14] by reconvolution of a (multi-)exponential fit with the internal response function fitting the measured decay and the amplitude-averaged lifetime was calculated. By this, distortions of the measured decay due to the SpectraLED pulse length and scattering are considered. However, the errors of the decay constants are estimated to be approximately ± 10%. The internal response function was measured for each measurement time range using a scattering cuvette and the monochromators set to the excitation wavelength. The amplitude-averaged lifetimes τ_{av} were calculated according to equation 1.4.

$$\tau_{av} = \frac{\sum_{n=1}^{3} A_n \cdot \tau_n}{\sum_{n=1}^{3} A_n} \qquad \text{eq. 1.4}$$

Further, the temperature-dependent lifetimes were fitted to extract the energy difference ΔE_{ST} between triplet state T_1 and the singlet state S_1 using the following equation (eq. 1.5):

$$\tau(T) = \frac{3+\exp(-\frac{\Delta E_{\mathrm{ST}}}{kT})}{\frac{3}{\tau(\mathrm{T_1})} + \frac{1}{\tau(\mathrm{S_1})}\exp(-\frac{\Delta E_{\mathrm{ST}}}{kT})} \qquad \text{eq. 1.5}$$

where kT corresponds to the thermal energy and $\tau(T_1)$ and $\tau(S_1)$ to the excited states' phosphorescence and fluorescence lifetimes, respectively.[15]

The temperature control and preparation of the KBr pellets are described in detail in the previous section.

Steady-state FTIR and Step-scan FTIR Spectroscopy. The steady-state FTIR and step-scan FTIR spectra were recorded using the BRUKER VERTEX80V. The spectrometer is equipped with a heated silicon carbide rod for generation of radiation in the MIR region and a liquid nitrogen cooled mercury cadmium telluride detector from KOLMAR TECHNOLOGIES KV100-1-B-7/190 for detection. For the time-resolved step-scan measurements, an additional fast preamplifier combined with a transient recorder board from SPECTRUM GERMANY MODEL M3I4142 with a time resolution down to 2.5 ns was used. The sample was excited using a pulsed Q-switched Nd:YAG laser from INNOLAS SPITLIGHT MODEL EVO I with a repetition rate of 100 Hz and a pulse width of 6 ns. The generated 1064 nm was modulated to 355 nm using a second and third harmonic generation stage and attenuated to 1.4-1.8 mJ with neutral density filters. The generated excitation light was transmitted to the sample chamber and adjusted to maximal overlap with the IR beam. To prevent scattering of the UV radiation to the MCT detector, the optical IR apertures were covered with germanium windows. The step-scan FTIR experiment was synchronized with the laser pulse using a STANFORD RESEARCH SYSTEMS DG535 delay generator. To prevent IR intensity outside the measured region from reaching the detector, a broadband filter from 850 to 1750 cm^{-1} was used. All spectra were recorded from 1750 up to 1180 cm^{-1} with a resolution of 4 cm^{-1}. The solely excited state spectra were generated by the addition of the ground state spectra intensity and were baseline corrected using a constant shift. Further, the obtained decay traces were fitted using a triexponential modified Gaussian using the following equation (eq. 1.6).

$$f(x) = y_0 + \sum_{n=1}^{3}\left(\frac{A_n}{\tau_n}\cdot \exp\left(\frac{1}{2}\left(\frac{\omega}{\tau_n}\right)^2 - \left(\frac{x-x_c}{\tau_n}\right)\right)\cdot\frac{\operatorname{erf}\left(\frac{z_n}{\sqrt{2}}\right)+1}{2}\right) \qquad \text{eq. 1.6}$$

$$z_n = \frac{x-x_c}{\omega} - \frac{\omega}{\tau}$$

The amplitude averaged lifetimes τ_{av} were calculated using eq. 1.4. For temperature control a closed-cycle helium cryostat from ARS MODEL DE202A was used and modified with a KBr pellet holder. The head of the cryostat is equipped with CaF_2 windows for transmittance in the IR region. Further, to check for possible photodegradation of the sample, the **C4-ref** was irradiated for 20 min at 355 nm and 1.4 mJ per pulse and FTIR spectra were recorded every 60 seconds. The resulting absorption spectra showed no changes in intensity or band position (see Figure S8.1). Thus, the sample facilitates sufficient stability for the cryogenic and time-resolved step-scan spectra.

Electrochemistry. Electrochemical measurements were performed on a METROHM AUTOLAB PGSTAT204 potentiostat in deaerated acetonitrile containing 1 mM of the analyte and 0.1 M $[Bu_4N][PF_6]$ as the supporting electrolyte. A three-electrode configuration was utilized, consisting of a glassy carbon working electrode with a diameter of 3 mm, a platinum wire counter electrode, and a non-aqueous Ag/Ag^+ reference electrode. This configuration was employed throughout the experimental process. Cyclic voltammograms were recorded at a scan rate of 100 mV s^{-1}.

Photostability. UV/vis absorption spectra were conducted using an AVANTES AVASPEC-ULS2048CL-EVO-RS spectrophotometer. A 150 W xenon arc lamp (*LOT-QUANTUMDESIGN GMBH*, LSE140/160.25C) was operated at 112 W and used as light source. The measurements were carried out under ambient conditions in a sealed quartz glass cuvette (10 mm) equipped with a magnetic stir bar. Samples were dissolved in acetonitrile (c = 0.02 mM, V = 3 mL) and stirred at 500 rpm.

Singlet Oxygen Measurement. Singlet-oxygen quantum yields (ϕ^1O_2) were determined by monitoring the near-infrared phosphorescence of $^1O_2$ at ca. 1276 nm using a HORIBA JOBIN-YVON FLUOROMAX PLUS-C spectrofluorometer. The instrument was equipped with a 150 W Xe-arc excitation source, CZERNY-TURNER monochromators fitted with an NIR grating blazed at 1000 nm, a R13456 photomultiplier-tube detector (190-930 nm) and a liquid-nitrogen–cooled DSS-IGA020L InGaAs photodiode (800-1550 nm). To ensure sample integrity, absorption spectra were recorded before and after each luminescence measurement on a JASCO V-770 UV–VIS–NIR spectrometer. Emission spectra of each sample were collected upon excitation at 390 nm. Phenalenone (ϕ^1O_2 = 0.98 in the literature[16]) was employed as an external standard under identical instrumental settings. Quantum yields for the unknowns were then calculated (*cf.* eq. 1.2) by comparing their integrated intensities to that of phenalenone with an estimated uncertainty of ± 10%.

Photocatalytic H_2 Generation. The experimental setup involves a 150 W xenon lamp LSE140 from LOT-QUANTUM DESIGN GMBH as the light source and an automatic gas burette system GASMESS-5 from MESSEN NORD GMBH for measuring hydrogen production. A thermostatically controlled reaction vessel connected to the burette ensures stable conditions. Gas evolution is monitored using a sensitive pressure sensor, with the hydrogen volume directly measured by pressure changes. Before measurements, the system was purged with argon to remove oxygen. The reactor was then loaded with 3.5 µmol of the respective photosensitiser (PS) and 5.0 µmol of the water reduction precatalyst [$Fe_3(CO)_{12}$]. 5 mL Tetrahydrofuran, 1.25 mL H_2O and 3.75 mL triethylamine were added under inert conditions using Schlenk technique to give a total volume of 10 mL.

Turnover numbers (TON) and hydrogen volumes ($\mathrm{V}_{\mathrm{m},H_2,25°\mathrm{C}}$) were calculated using the following equations (eq. 1.7 and eq. 1.8):

$$\mathrm{TON}_{\mathrm{PS},H_2,25°\mathrm{C}} = \frac{\mathrm{V_{obs}} - \mathrm{V_{blank}}}{\mathrm{V}_{\mathrm{m},H_2,25°\mathrm{C}} \cdot \mathrm{n(PS)}} \cdot 2 \qquad \text{eq. 1.7}$$

$$\mathrm{V}_{\mathrm{m},H_2,25°\mathrm{C}} = \frac{\mathrm{RT}}{\mathrm{p}} + \mathrm{b} - \frac{\mathrm{a}}{\mathrm{RT}} = 23.50\ \frac{\mathrm{mL}}{\mathrm{mmol}} \qquad \text{eq. 1.8}$$

Photocatalytic Dehalogenation. The vial was charged with 0.05 mmol of BIH, evacuated and filled with argon. Under inert atmosphere, 0.5 mmol TEA was added. Afterwards 1.5 mL of a previously prepared solution of 0.05 mmol starting material, 0.0005 mmol Cu(I)-photosensitizer and 0.01705 mmol *n*-hexadecane in 1.5 mL MeCN was added to the vial under inert conditions. The vial was sealed with a screw cap with a Teflon plate, and placed in the photoreactor (blue LED irradiation (λ = 460 nm)) for the respective reaction time of 15 minutes for E_1 (4-bromobenzophenone) or 5 hours for E_2 (2-bromoacetanilide). The temperature of the solution was cooled by an airstream and the distance to the light source was kept constant. After the reaction, 30 µL of the solution was diluted in 1 mL DCM and the amount of Ar-H and Ar-X was determined by GC-FID analysis, using *n*-hexadecane as the internal standard. All measurements were performed twice in order to estimate uncertainty. Control experiments confirmed that light, photocatalyst, and sacrificial donors are all needed.

2 Synthetic Details

General Techniques. All reactions were carried out under an argon atmosphere, unless otherwise specified. Standard Schlenk techniques were employed. Glassware underwent vacuum drying and then was flushed with argon. This process was repeated a total of three times to ensure complete removal of any residual moisture or air. For stirring and heating purposes, magnetic stirring bars, stirrers and hotplates from IKA were used. Solvent removal was achieved using a rotary evaporator from HEIDOLPH followed by drying under high vacuum (10^{-3} mbar) and heating at 45 °C. For weighing of substances, a balance (EG 420-3NM from KERN for approximate weighing, over 100 mg mass and a PX125D from OHAUS for more accurate weighing) were used.

Chemicals and Solvents. The chemicals utilized in the experiments were purchased from different suppliers such as SIGMA-ALDRICH, CARL ROTH or BLDPHARM and were used as received unless otherwise specified. Solvents, acids, and bases were provided from the chemical supply of the Chemistry Institute at the Technische Universität of Braunschweig. Dry, inert solvents were obtained through distillation over suitable drying agents under an inert atmosphere. Degassed water was prepared in a Schlenk flask using an ultrasonic bath (BANDELIN) under reduced pressure for 30 min, followed by purging with argon. Solvents used for column chromatography were employed without further treatment.

Thin Layer Chromatography. To monitor the progress of the reaction, neutral aluminium oxide plates (*60 F254*) from MERCK were employed and visualized using UV detection (λ = 254 nm or 365 nm).

Column Chromatography. Column chromatography utilized aluminum oxide (90 active neutral) from MERCK as the stationary phase. Overpressure was applied manually using a pump ball to facilitate the chromatographic process. Appropriate column size selection was crucial for efficient separation. Separation progress was monitored using a UV lamp (λ = 254 nm or 365 nm) to observe emission from derivatives. Efforts were made to recycle solvents, when possible, to promote sustainability.

Synthetic Procedure

The synthesis of the ligands **L3-p** and **L4-ref** and the complexes **C3-p** and **C4-ref** were adapted from literature.[17,18] For the new ligands **L1-o** and **L2-m** and complexes **C1-o** and **C2-m**, the synthetic procedure is described below.

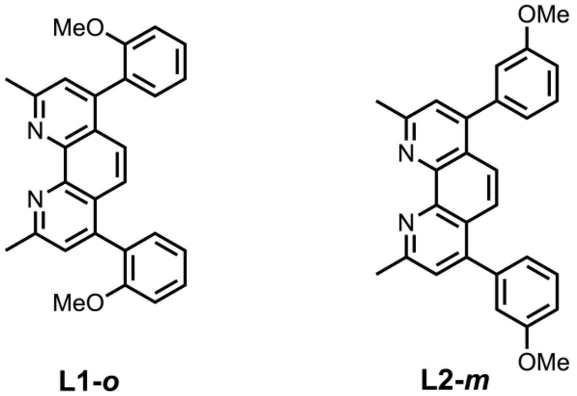

L1-*o* **L2-*m***

Figure S1. Molecular structures of **L1-o** (left) and **L2-m** (right).

4,7-dichloro-neocuproine (200 mg, 0.72 mmol, 1 eq.), boronic acid (329 mg, 2.17 mmol, 3 eq.), Xphos-Pd-G2-Cat. (17.0 mg, 0.02 mmol, 3 mol%) and tripotassium phosphate salt (2604 mg, 12.27 mmol, 17 eq.) were introduced into a 100 mL dried Schlenk round-bottom flask equipped with a magnetic stir bar. The vessel was attached to a reflux cooler and then evacuated and backfilled with argon (this process was repeated three times). The mixture was dissolved in degassed tetrahydrofuran (18 mL) and degassed water (20 mL). The reaction was heated to 75 °C and refluxed overnight (16 h) under strong stirring. Tetrahydrofuran was evaporated and the aqueous phase was extracted with dichloromethane three times. The combined organic phases were washed with aq. NaOH (2 M) three times, then with water twice. The organic phase was dried with $MgSO_4$ and the solvent was removed. The crude product was flashed through a short plug of aluminium oxide (neutral, dichloromethane, 1% MeOH). The ligands **L1-o** (215 mg, 71 %) and **L2-m** (207 mg, 68 %) were obtained as white-beige solids.

L1-o ($C_{28}H_{24}N_2O_2$, M: 420.51 g/mol): **^{1}H NMR** ($CDCl_3$, 600 MHz, 25 °C) δ [ppm] = 7.44 (m, 2H, Ar), 7.40 (s, 1H, Ar), 7.39 (s, 1H, Ar), 7.38 (s, 1H, Ar), 7.36 (s, 1H, Ar), 7.29 (*dd*, *J* = 7.4 Hz, *J* = 1.7 Hz, 1H, Ar), 7.26 (*dd*, *J* = 7.4 Hz, *J* = 1.7 Hz, 1H, Ar), 7.08 (*dtd*, *J* = 11.8 Hz, *J* = 7.4 Hz, *J* = 1.0 Hz, 2H, Ar), 7.03 (*t*, *J* = 9.0 Hz, 2H, Ar), 3.73 (*s*, 3H, OMe), 3.71 (*s*, 3H, OMe), 2.98 (*s*, 6H, CH_3). **^{13}C{^{1}H} NMR** ($CDCl_3$, 150 MHz, 25 °C) δ [ppm] = 158.4, 156.7, 145.7,145.6, 131.3, 129.8, 127.3, 125.5, 124.4, 123.2, 120.7, 111.1, 55.5, 25.9. **DEPT 135 NMR** ($CDCl_3$, 150 MHz, 25 °C) δ [ppm] 131.3(+), 129.8(+), 124.4(+), 123.2(+), 120.7(+), 111.0(+), 55.5(+), 25.9(+). **HRMS (ESI)** *m/z*: calcd. for [**L1-o**+H]$^+$: 421.1916, found: 421.1913.

L2-m ($C_{28}H_{24}N_2O_2$, M: 420.51 g/mol): **^{1}H NMR** ($CDCl_3$, 400 MHz, 25 °C) δ [ppm] = 7.77 (s, 2H, Ar), 7.45 (s, 2H, Ar), 7.42 (*t*, *J* = 7.8 Hz, 2H, Ar), 7.09 (*dt*, *J* = 7.5 Hz, *J* = 1.2 Hz, 2H, Ar), 7.03 (*m*, 4H, Ar), 3.86 (s, 6H, OMe), 2.99 (s, 6H, CH_3). **^{13}C{^{1}H} NMR** ($CDCl_3$, 100 MHz, 25 °C) δ [ppm] = 159.7, 158.7, 148.4, 146.0, 139.6, 129.5, 124.7, 123.8, 123.0, 122.1, 115.2, 113.9, 55.4, 26.0. **DEPT 135 NMR** ($CDCl_3$, 100 MHz, 25 °C) δ [ppm] 129.6(+), 123.8(+), 123.0(+), 122.1(+), 115.3(+), 114.0(+), 55.5(+), 26.0(+). **HRMS (ESI)** *m/z*: calcd. for [**L2-m**+H]$^+$: 421.1916, found: 421.1913.

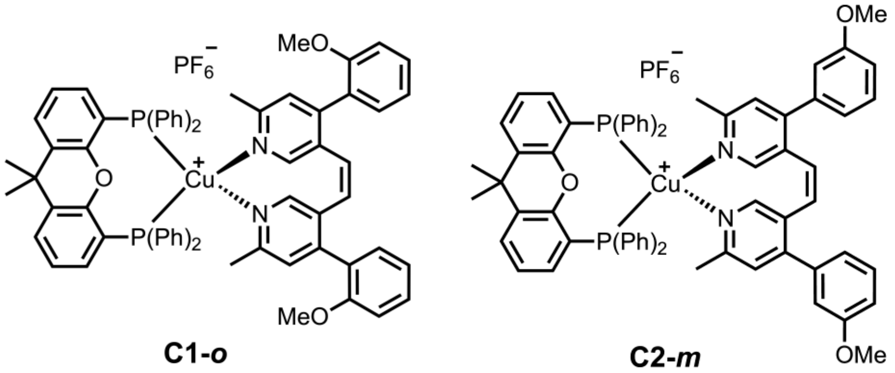

Figure S2. Molecular structures of **C1-o** (left) and **C2-m** (right).

$[Cu(MeCN)_4]PF_6$ (62.1 mg, 0.17 mmol, 1 eq.) and xantphos (96.3 mg, 0.17 mmol, 1 eq.) were introduced into a 100 mL dried Schlenk tube equipped with a magnetic stir bar. The vessel was attached to a reflux cooler and then evacuated and backfilled with argon (this process was repeated 3 times). The mixture was dissolved in degassed dichloromethane (20 mL), which was added *via* a syringe. The reaction was heated to 45 °C and refluxed for 16 h under strong stirring. The solution was first cooled to rt and then 0 °C. Under strong stirring, a solution of the ligand (80.0 mg, 0.17 mmol, 1 eq.) in dry and degassed dichloromethane (15 mL) was added (dropwise) at 0 °C using a syringe pump (ca. 10 mL h^{-1}). After complete addition, stirring was continued for 30 min at 0 °C followed by 3 h at 45 °C. The heteroleptic target complex was precipitated with *n*-hexane in the Schlenk tube. The solution was stored at +4 °C overnight. The solid product was collected, washed with *n*-hexane and diethyl ether and dried under vacuum to afford 174 mg (76 %) of **C1-o** and 177 mg (77 %) of **C2-m** as yellow crystals.

C1-o ($C_{67}H_{56}CuF_6N_2O_3P_3$, M: 1207.65 g/mol): **^{1}H NMR** (CD_3CN, 500 MHz, 25 °C) δ [ppm] = 7.76 (*m*,2H, Ar), 7.53 (*ddd*, *J* = 8.5 Hz, *J* = 7.6 Hz, *J* = 1.9 Hz 2H, Ar), 7.44 (*s*, 1H, Ar), 7.43 (*s*, 1H, Ar),7.35 (*d*, *J* = 2.2 Hz, 2H, Ar), 7.32 (*t*, *J* = 7.3 Hz 2H, Ar), 7.19 (*m*, 18H, Ar), 7.04 (*m,* 10H, Ar), 3.72 (*s*, 3H, OMe), 3.70 (*s*, 3H, OMe), 2.29 (*s*, 6H, CH_3), 1.72 (*s*, 6H, CH_3). **^{13}C{^{1}H} NMR** (CD_3CN, 125 MHz, 25 °C) δ [ppm] = 159.0, 157.6, 156.0, 148.7, 144.1, 135.0, 134.6, 134.4, 134.2, 133.8, 133.4, 132.6, 132.0, 131.3, 131.2, 130.8, 129.8, 129.5, 128.9, 127.3, 126.4, 124.7, 122.8, 122.0, 112.7, 56.3, 37.0, 28.9, 27.8. **DEPT 135 NMR** (CD_3CN, 125 MHz, 25 °C) δ [ppm] 134.6(+), 134.4(+), 134.2(+), 133.8(+), 133.4(+), 132.0(+), 131.3(+), 131.2(+), 130.8(+), 129.8(+), 129.5(+), 128.9(+), 127.1(+), 126.4(+), 124.7(+), 122.0(+), 112.7(+), 56.3(+), 28.9(+), 27.8(+). **^{31}P{^{1}H} NMR** (CD_3CN, 202 MHz, 25 °C) δ [ppm] = -11.7 (s, Ar), -143.2 (septet, *J* = 706 Hz, PF_6). **HRMS (ESI)** *m/z*: calcd. for [**C1-o**-PF_6]$^+$: 1061.3062, found: 1061.3063. **EA (CHN)** calcd.: 66.64, 4.67, 2.32, found: 65.16, 4.54, 2.14.

C2-m ($C_{67}H_{56}CuF_6N_2O_3P_3$, M: 1207.65 g/mol): **^{1}H NMR** (CD_3CN, 500 MHz, 25 °C) δ [ppm] = 7.76 (*dd*, *J* = 7.8 Hz, *J* = 1.4 Hz ,2H, Ar), 7.75 (*s*, 2H, Ar), 7.51 (*s*, 2H, Ar), 7.49 (*t*, *J* = 7.9 Hz, 2H, Ar), 7.27 (*m*, 6H, Ar), 7.09 (*m,* 24H, Ar), 3.85 (*s*, 6H, OMe), 2.31 (*s*, 6H, CH_3), 1.73 (*s*, 6H, CH_3). **^{13}C{^{1}H} NMR** (CD_3CN, 125 MHz, 25 °C) δ [ppm] = 161.1, 159.1, 155.9, 150.6, 144.5, 139.1, 135.0, 134.0, 132.6, 131.3, 131.1, 131.0, 129.6, 129.0, 126.6, 126.5, 126.4, 124.4, 122.9, 122.7, 116.2, 115.5, 56.2, 37.0, 28.9, 27.9. **DEPT 135 NMR** (CD_3CN, 125 MHz, 25 °C) δ [ppm] 134.0(+), 131.3(+), 131.1(+), 131.0(+), 129.6(+), 129.0(+), 126.6(+), 126.4(+), 124.4(+), 122.9(+), 116.2(+), 115.5(+), 56.2(+), 37.0(+), 28.9(+), 27.9(+). **^{31}P{^{1}H} NMR** (CD_3CN, 202 MHz, 25 °C) δ [ppm] = -11.6 (s, Ar), -143.2 (septet, *J* = 706 Hz, PF_6). **HRMS (ESI)** *m/z*: calcd. for [**C2-m**-PF_6]$^+$: 1061.3062, found: 1061.3064. **EA (CHN)** calcd.: 66.64, 4.67, 2.32, found: 66.24, 4.53, 2.21.

3 NMR

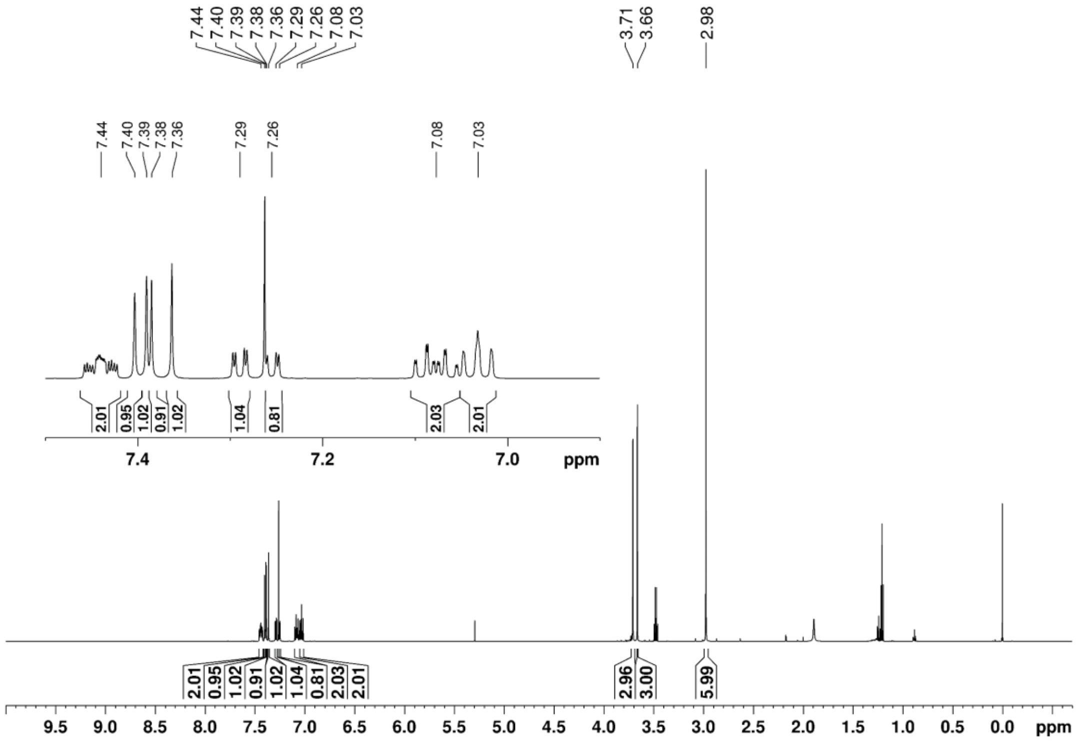

Figure S3. ^{1}H NMR of **L1-o** in $CDCl_3$ (600 MHz).

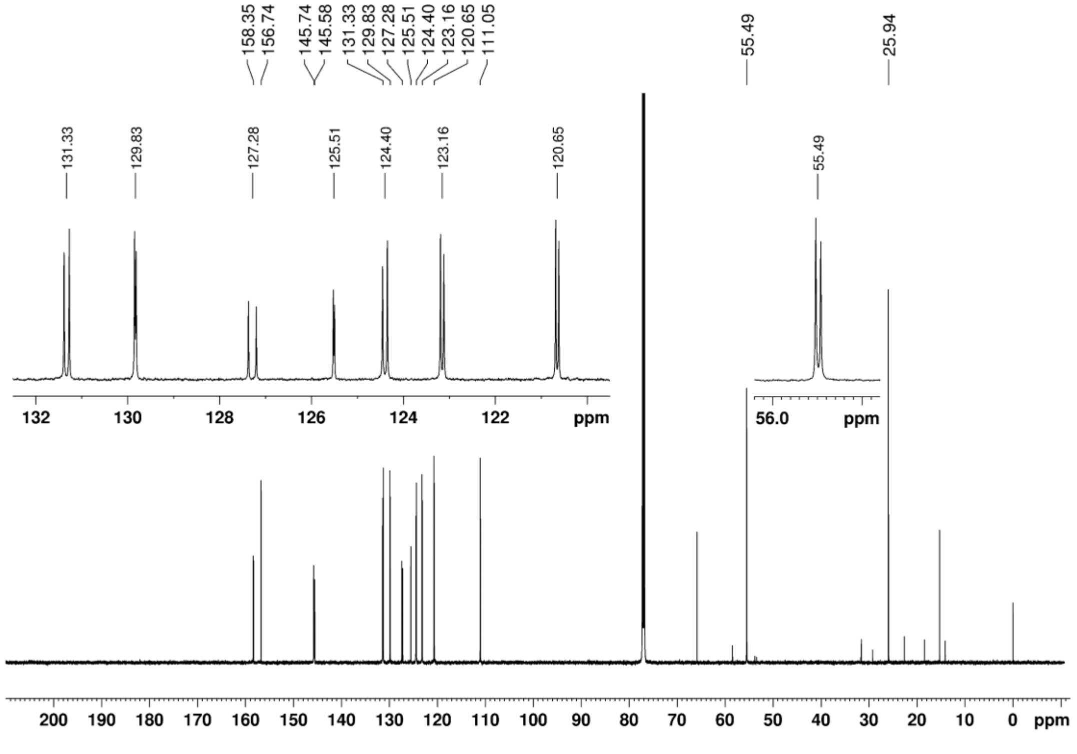

Figure S4. $^{13}C\{^{1}H\}$ NMR of **L1-o** in $CDCl_3$ (125 MHz).

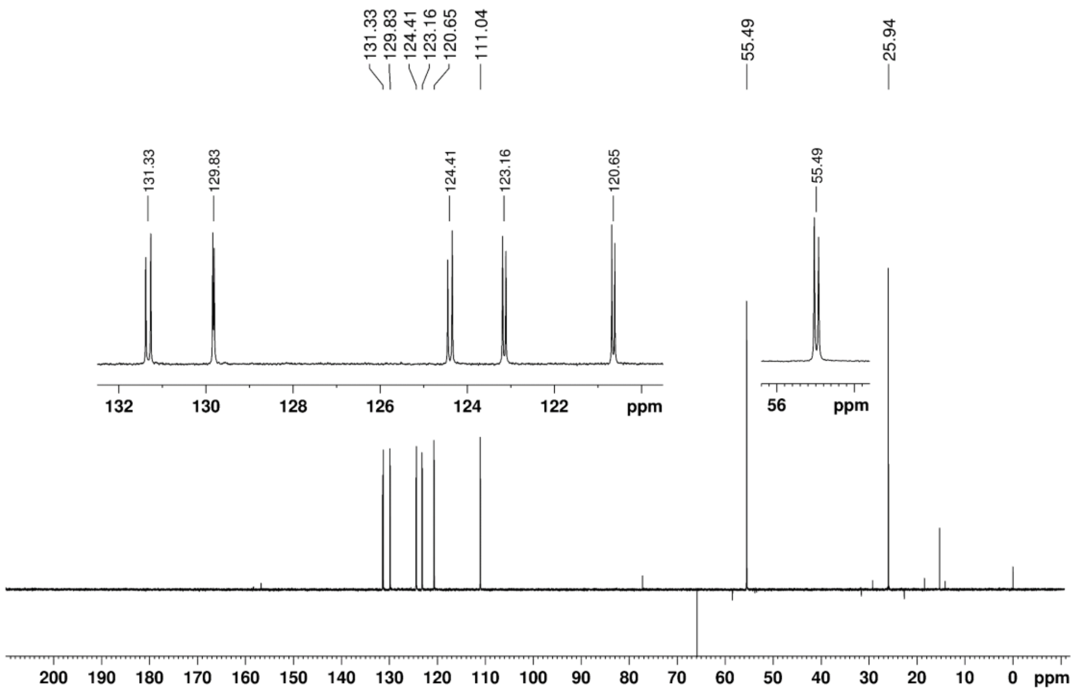

Figure S5. DEPT 135 NMR of **L1-o** in $CDCl_3$ (125 MHz).

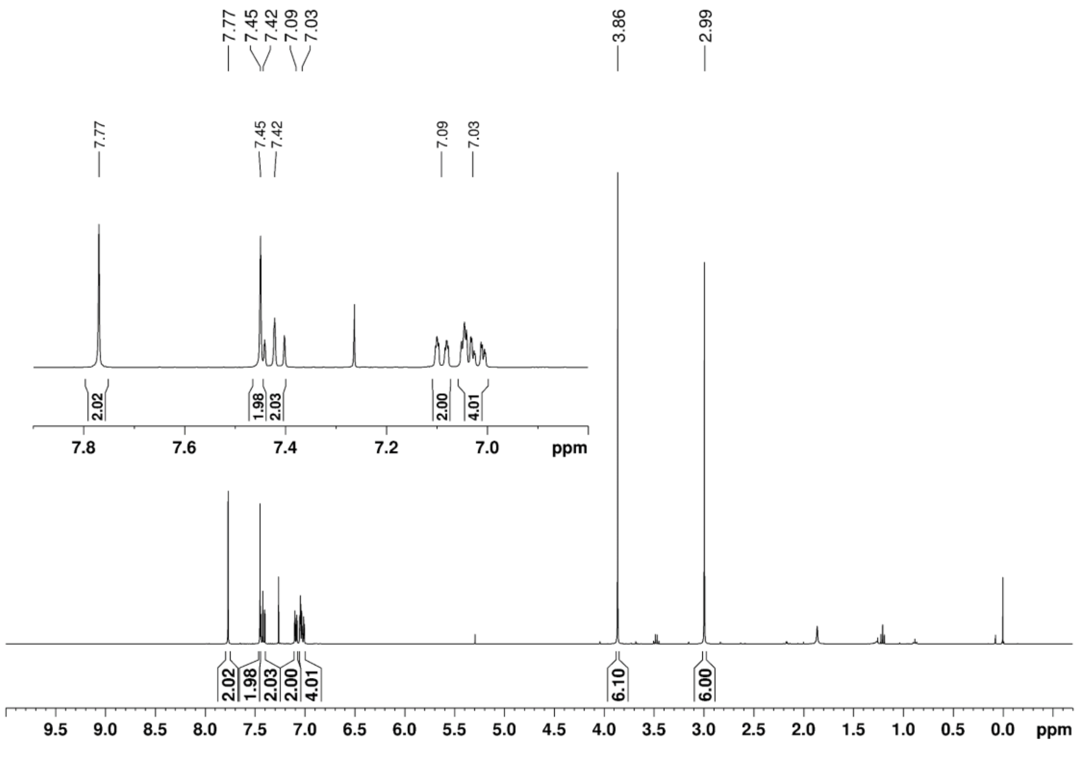

Figure S6. ^1H NMR of **L2-m** in $CDCl_3$ (400 MHz).

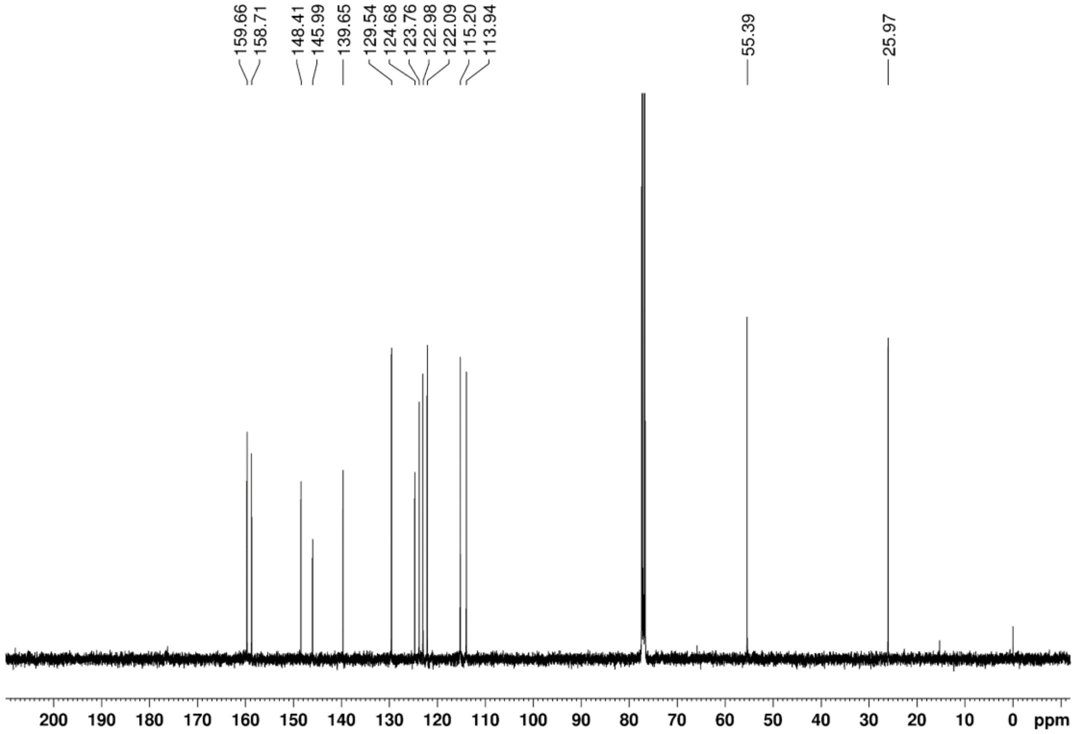

Figure S7. $^{13}C\{^{1}H\}$ NMR of **L2-m** in $CDCl_3$ (125 MHz).

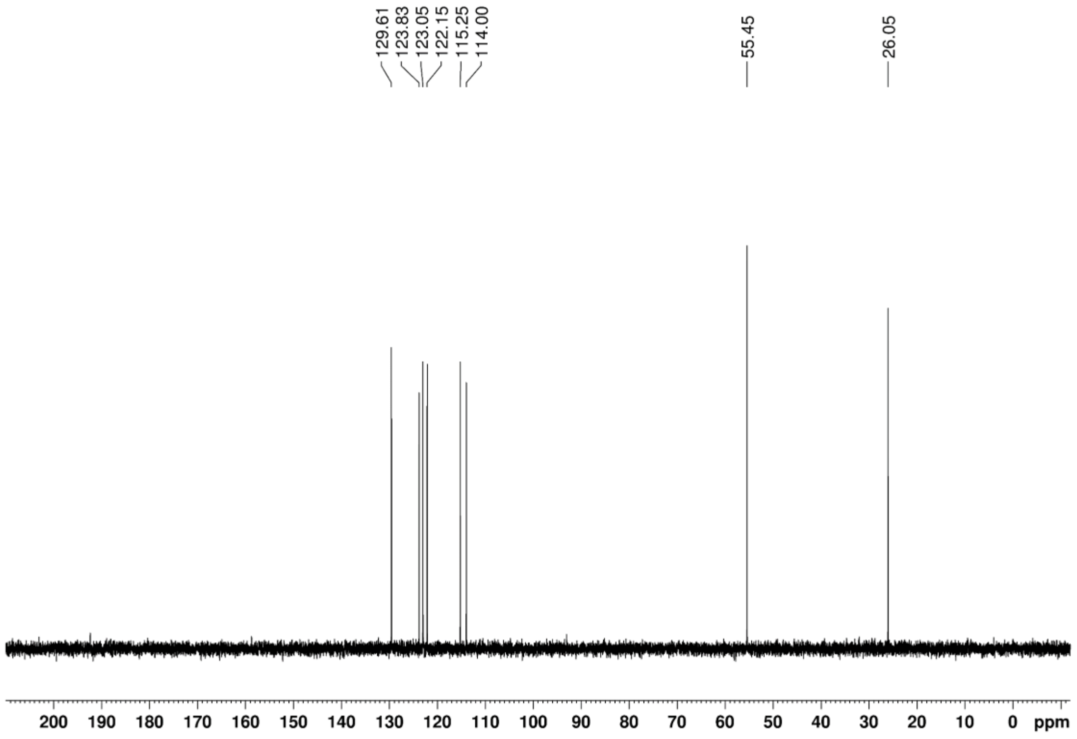

Figure S8. DEPT 135 NMR of **L2-m** in $CDCl_3$ (125 MHz).

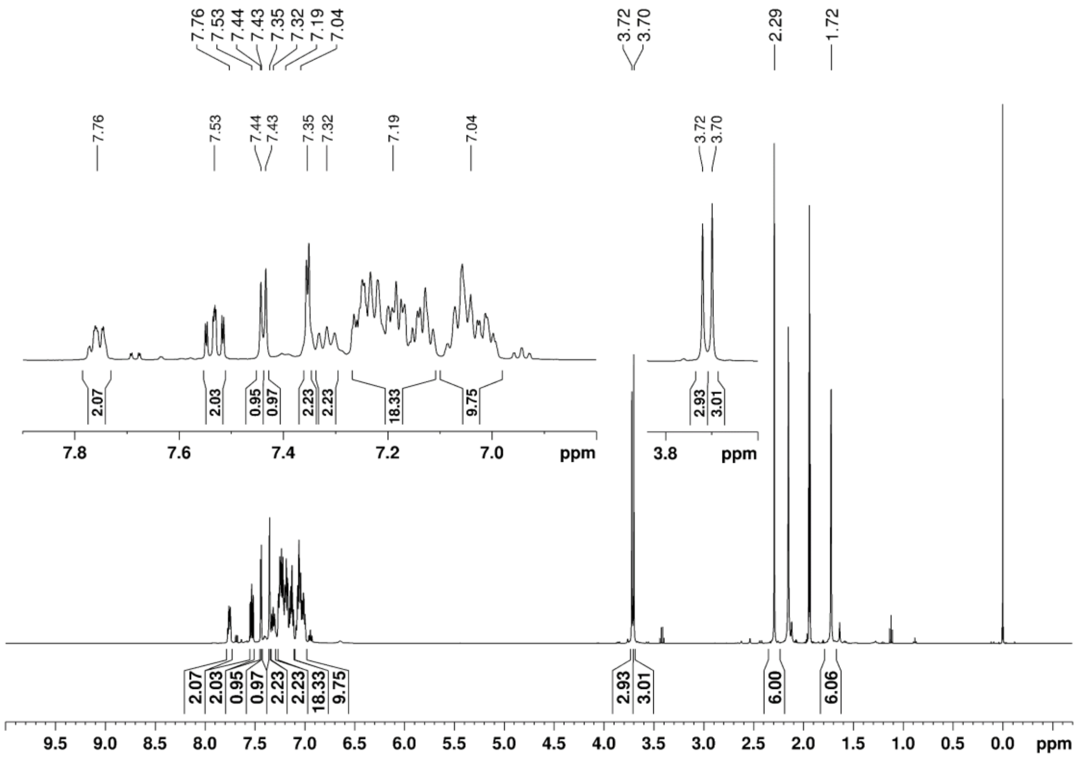

Figure S9. ^{1}H NMR of **C1-o** in CD_3CN (500 MHz).

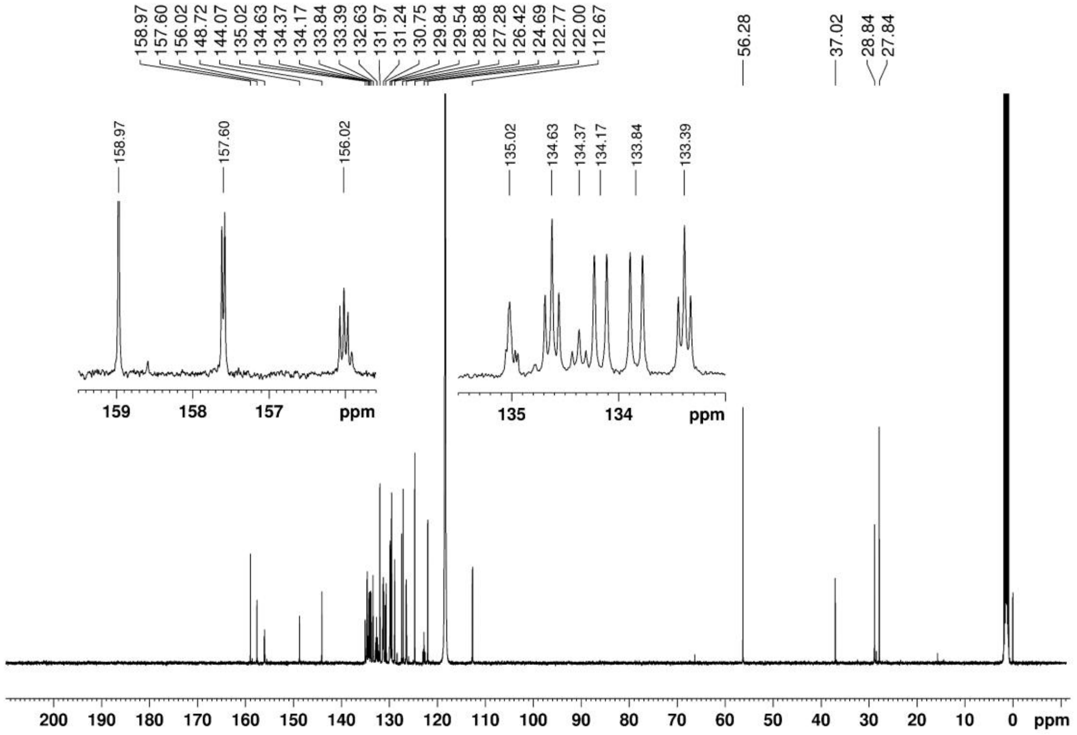

Figure S10. $^{13}C\{^{1}H\}$ NMR of **C1-o** in CD_3CN (125 MHz).

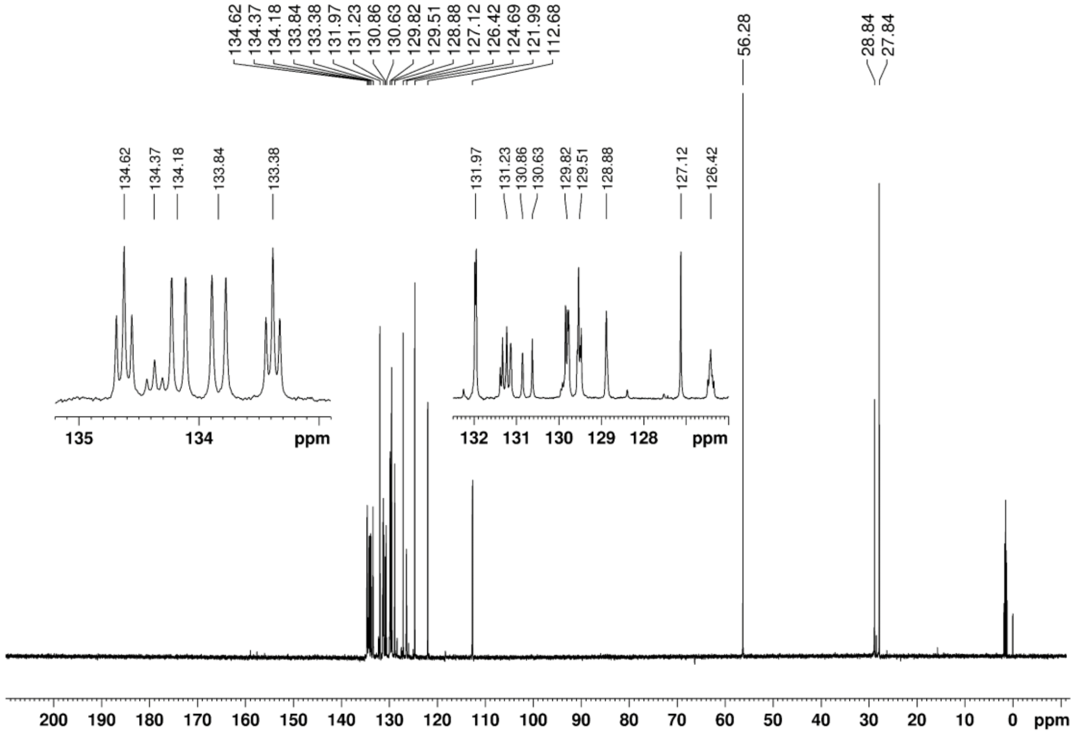

Figure S11. DEPT 135 NMR of **C1-o** in CD_3CN (125 MHz).

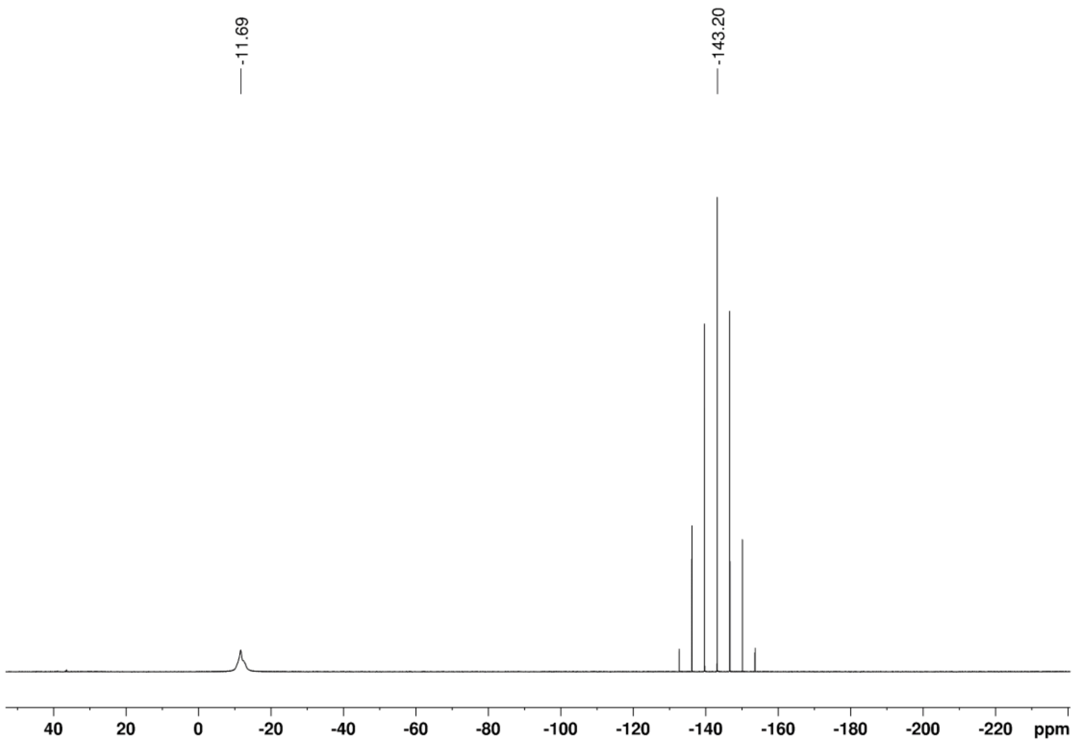

Figure S12. $^{31}P\{^{1}H\}$ NMR of **C1-o** in CD_3CN (202 MHz).

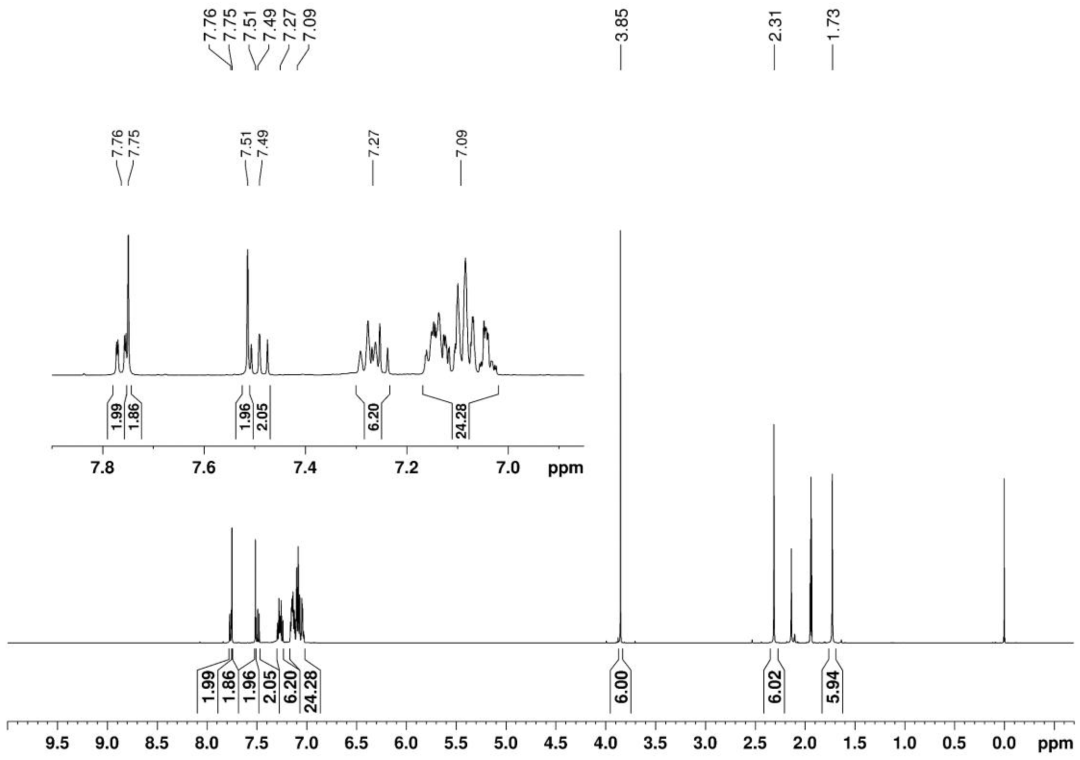

Figure S13. ^{1}H NMR of **C2-m** in CD_3CN (500 MHz).

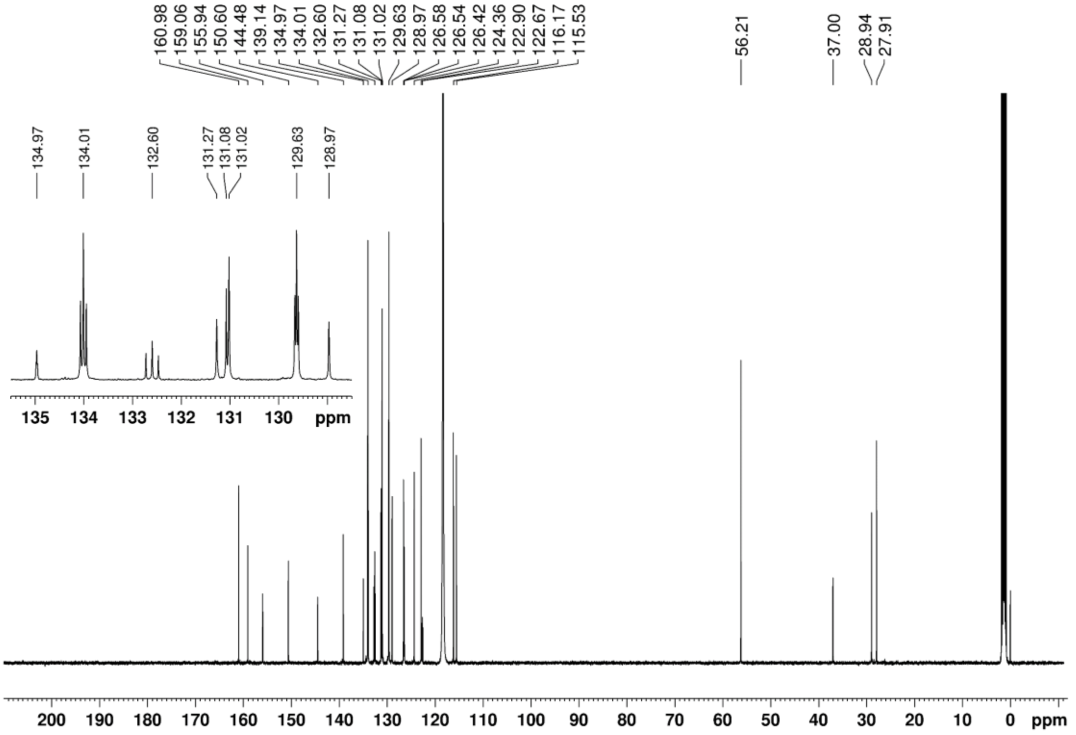

Figure S14. $^{13}C\{^{1}H\}$ NMR of **C2-m** in CD_3CN (125 MHz).

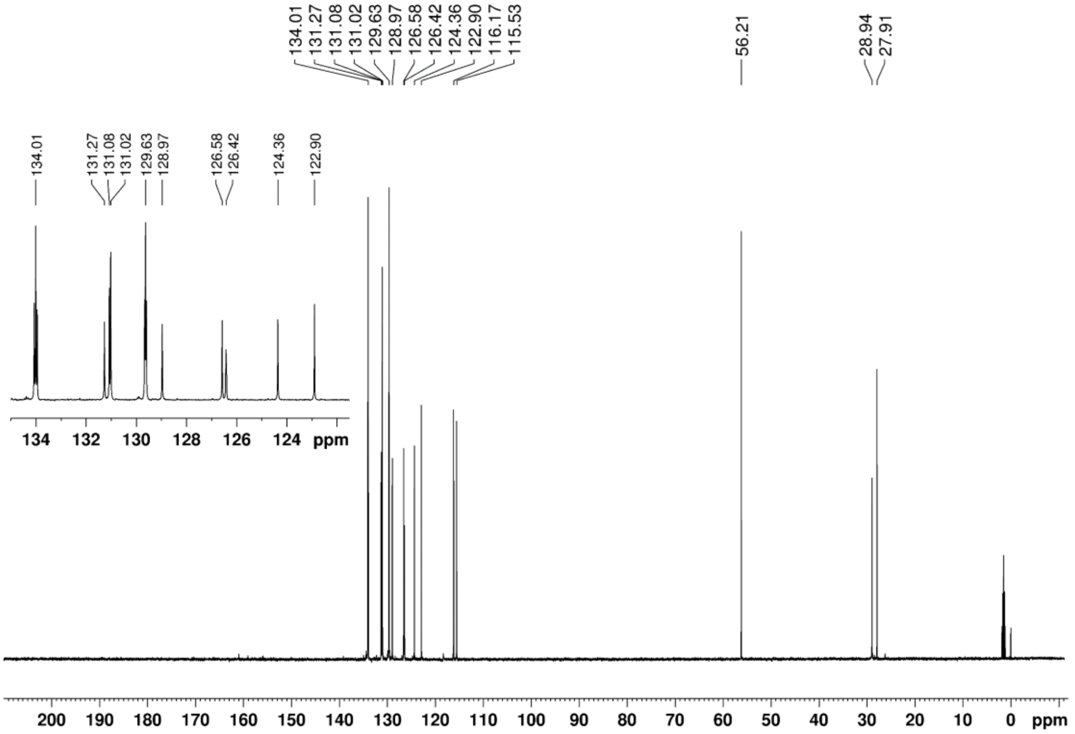

Figure S15. DEPT 135 NMR of **C2-m** in CD_3CN (125 MHz).

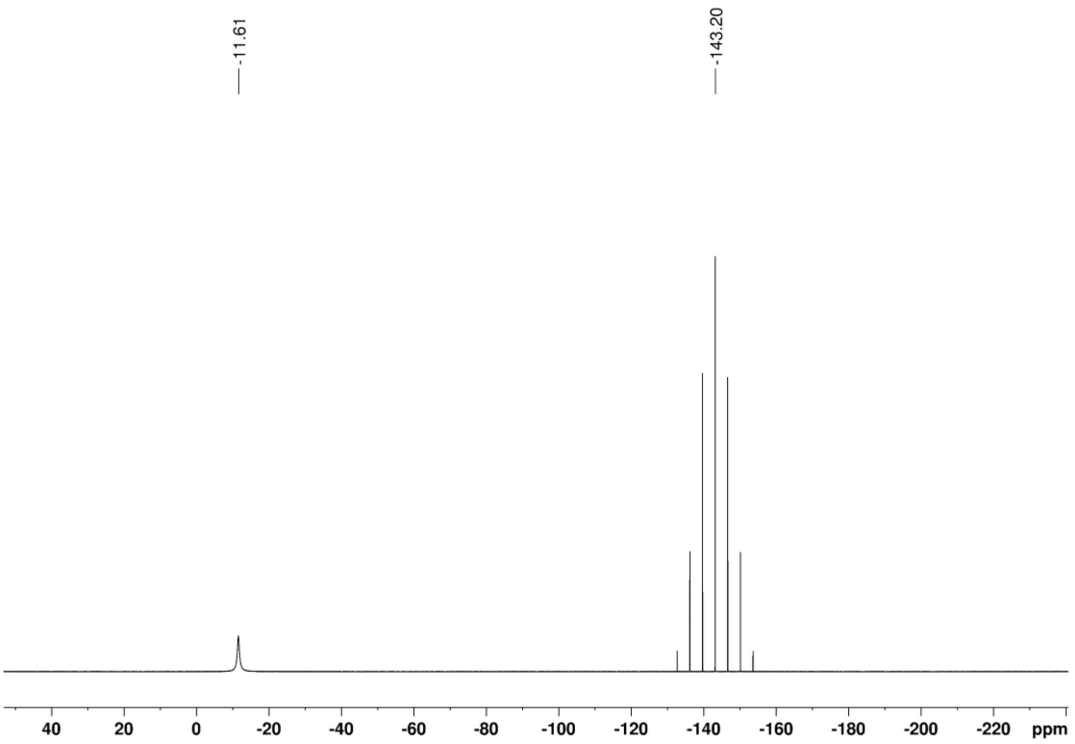

Figure S16. $^{31}P\{^1H\}$ NMR of **C2-m** in CD_3CN (202 MHz).

4 MS

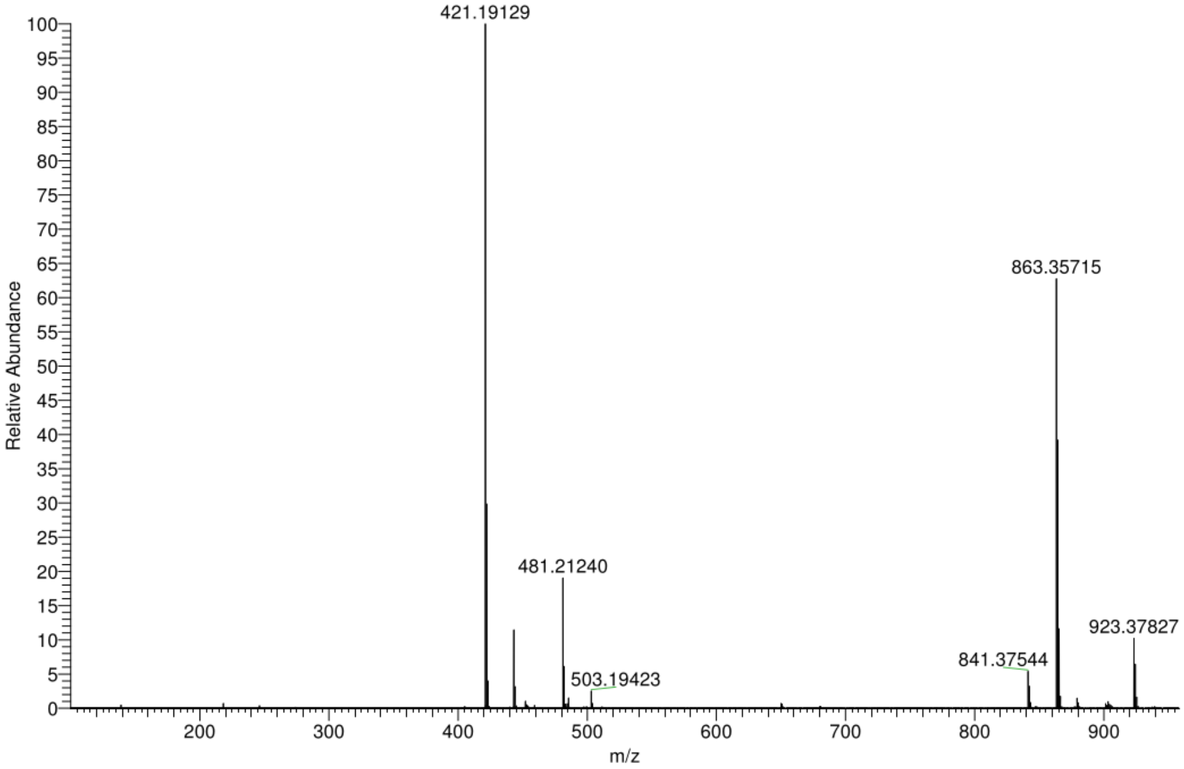

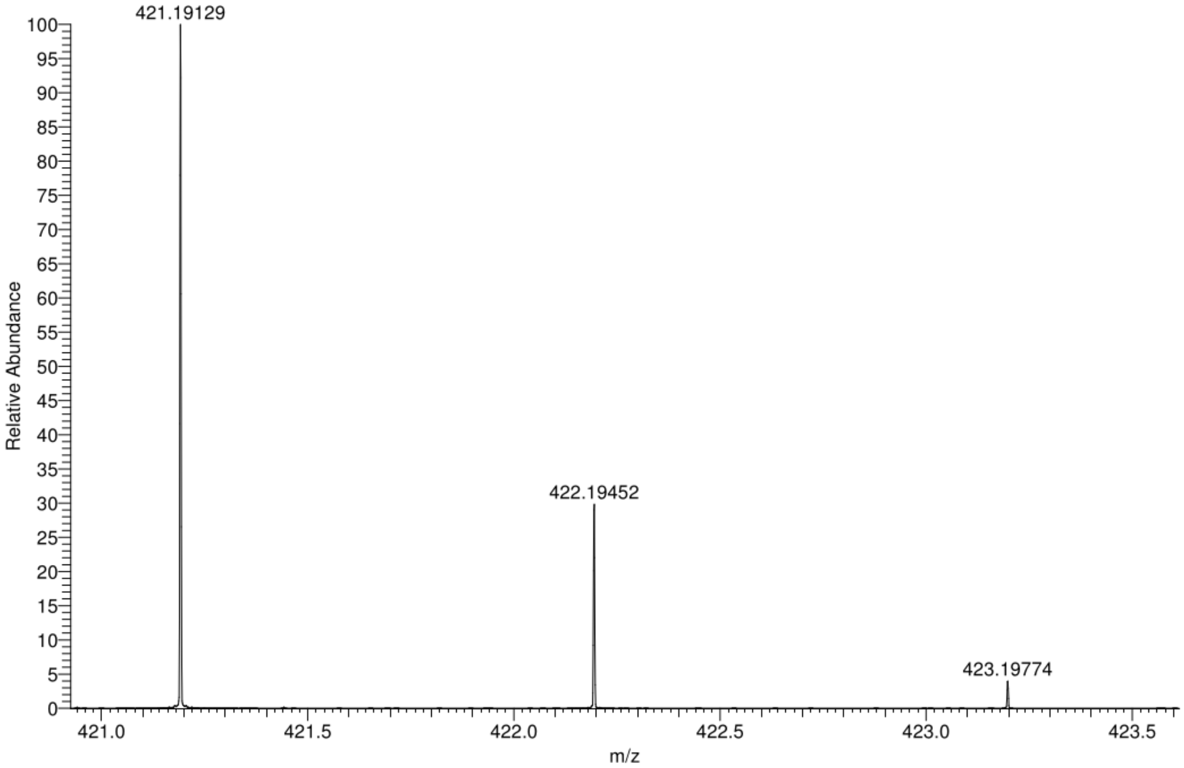

Figure S17. HRMS (ESI) of **L1-o** in MeOH as fullscan (top) and zoom (bottom).

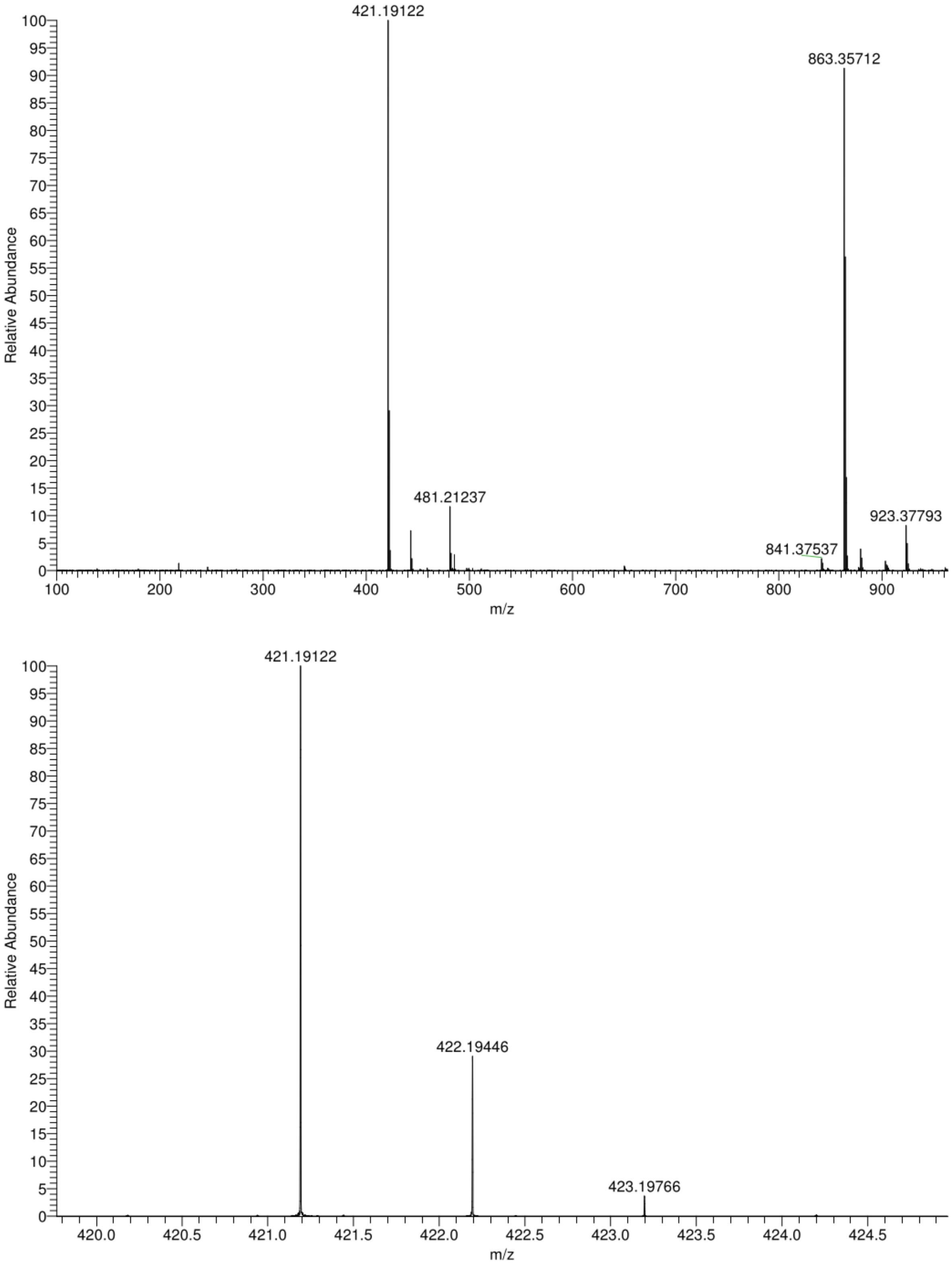

Figure S18. HRMS (ESI) of **L2-m** in MeOH as fullscan (top) and zoom (bottom).

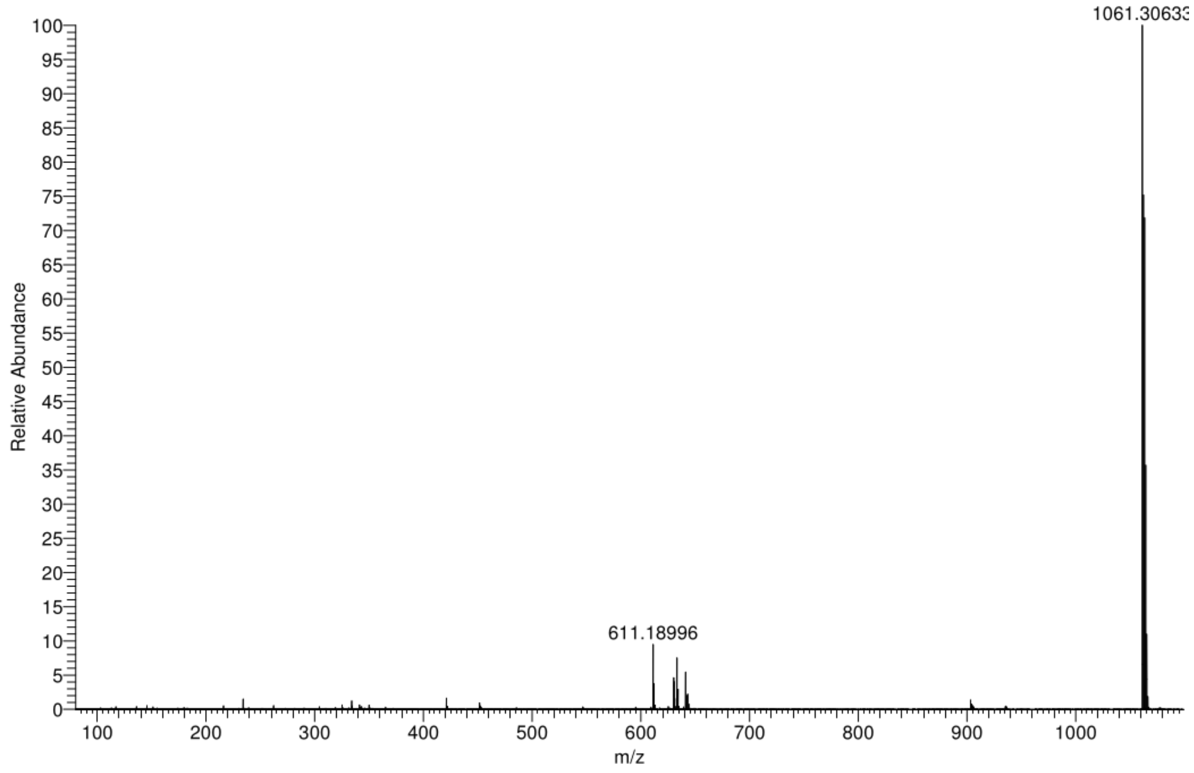

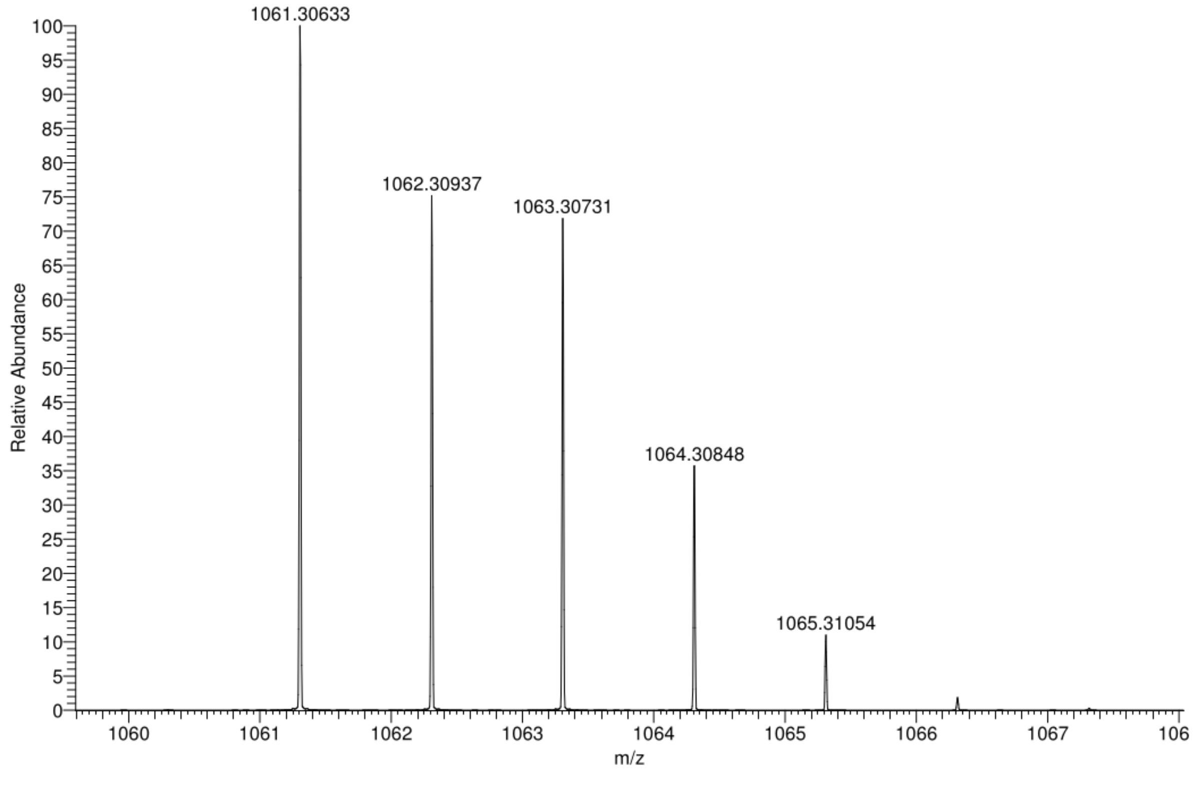

Figure S19. HRMS (ESI) of **C1-o** in MeOH as fullscan (top) and zoom (bottom).

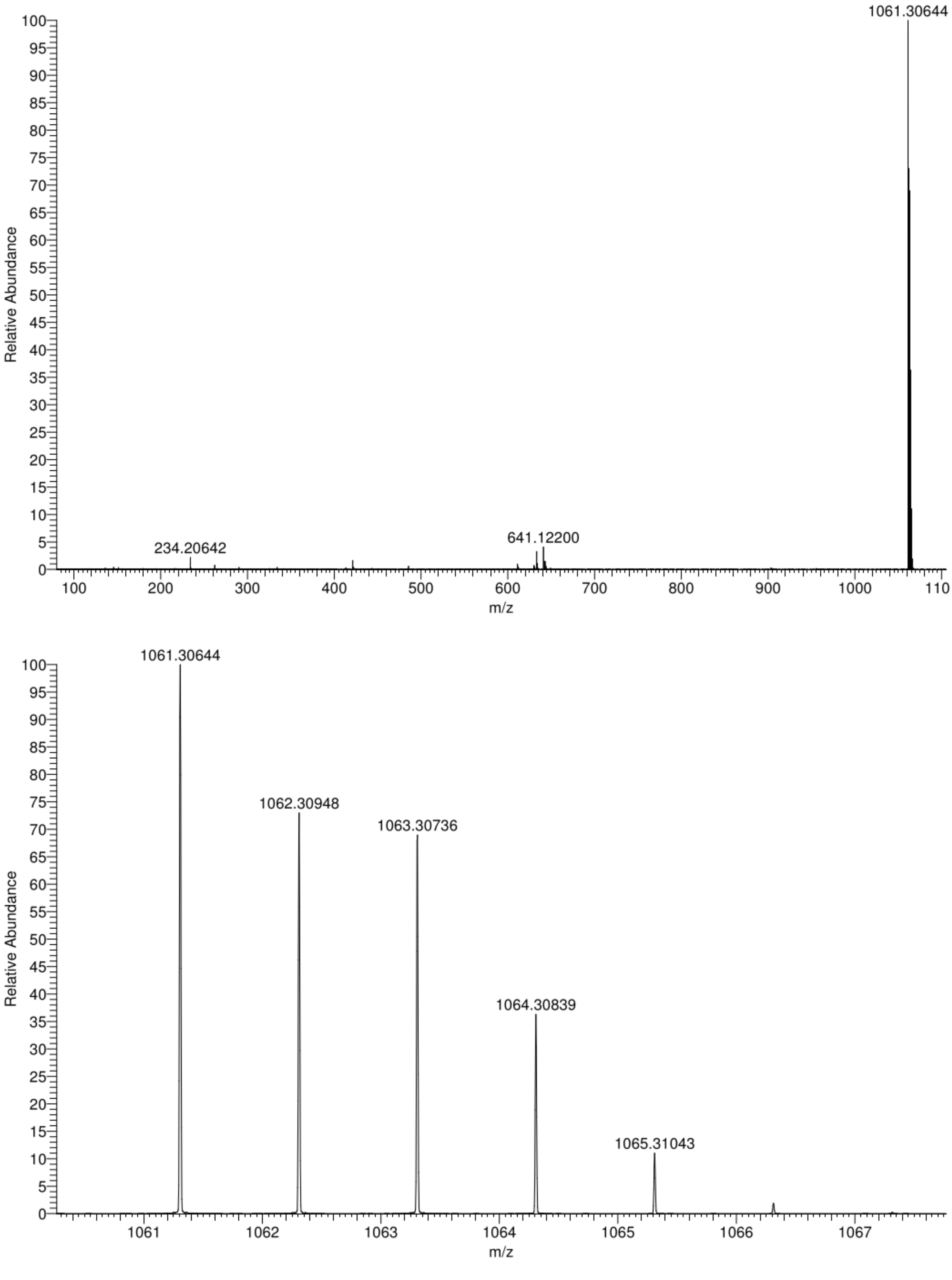

Figure S20. HRMS (ESI) of **C2-m** in MeOH as fullscan (top) and zoom (bottom).

5 Crystallographic Data

Table S1. Crystallographic data of the structural determination of **C2-m**.

	**C2-m**$(CH_2Cl_2)_2$[a]
Sum formula	$C_{69}H_{60}Cl_4CuF_6N_2O_3P_3$
Moiety formula	$C_{67}H_{56}CuN_2O_3P_2$, F_6P, 2 (CH_2Cl_2)
Formula mass	1377.44 g mol^{-1}
Density (calculated)	1.452 g cm^{-3}
Crystal shape, color	lath-like fragment (clear yellow)
Crystal size	0.470 × 0.120 × 0.040 mm^3
Temperature, Radiation	100(2) K, 1.54184 Å
Abs. coefficient	3.336 mm^{-1}
F(000)	2832
Crystal system	monoclinic
Space group type (no.)	$P2_1/c$ (no. 14)
Z, Z'	4, 1
a, b, c	12.39100(10) Å, 17.48960(10) Å, 29.1019(2) Å
α, β, γ	90°, 92.3710(10)°, 90°
Volume	6301.38(8) $Å^3$
Reflections collected	291563
indep.	13678
observed [$I>2\sigma(I)$]	12773
Data collection ranges	$-13 \le h \le 15$, $-22 \le k \le 22$, $-37 \le l \le 37$
Completeness to $\theta = 80.0°$	99.2%
Data / restr. / param.	13678 / 0 / 799
R_{int}	0.0398
R_1 [$I > 2\sigma(I)$]	0.0433
wR_2 (all data)	0.1175
GoF on F^2	1.077
Largest peak/hole	0.741 and –0.970e $Å^{-3}$
CCDC Number	CCDC 2482019
Crystallization Details	From a solution in CH_2Cl_2/*n*-hexane at rt

6 Density Functional Theory (DFT)

Table S2. Selected bond lengths (pm), bite angles (°), interplane angles between the N-Cu-N / P-Cu-P planes (PP-Cu-NN, °), and the torsion angles $\tau_{sub,1}$ (°) and $\tau_{sub,2}$ (°) of the backbone phenyl group of the geometry optimized T_1 excited state structures of **C2-m**, **C1-o**, **C3-p** and **C4-ref** predicted by DFT calculations.

	**C1-o**	**C2-m**	**C3-p**	**C4-ref**
**Cu-N**	197.3	197.3	197.4	197.4
	203.1	203.5	203.1	203.5
**Cu-P**	235.2	235.1	235.2	235.2
	238.9	239.0	239.3	239.0
**C-O**	137.6	137.6	137.6	137.6
	137.6	137.6	137.7	137.6
**N-Cu-N**	83.7	83.5	83.5	83.5
**P-Cu-P**	111.5	111.6	111.0	111.6
**PP-Cu-NN**	79.3	80.4	80.2	80.4
$\tau_{sub,1}$	-55.5	-48.0	-130.7	-48.0
$\tau_{sub,2}$	57.8	50.1	50.1	48.0

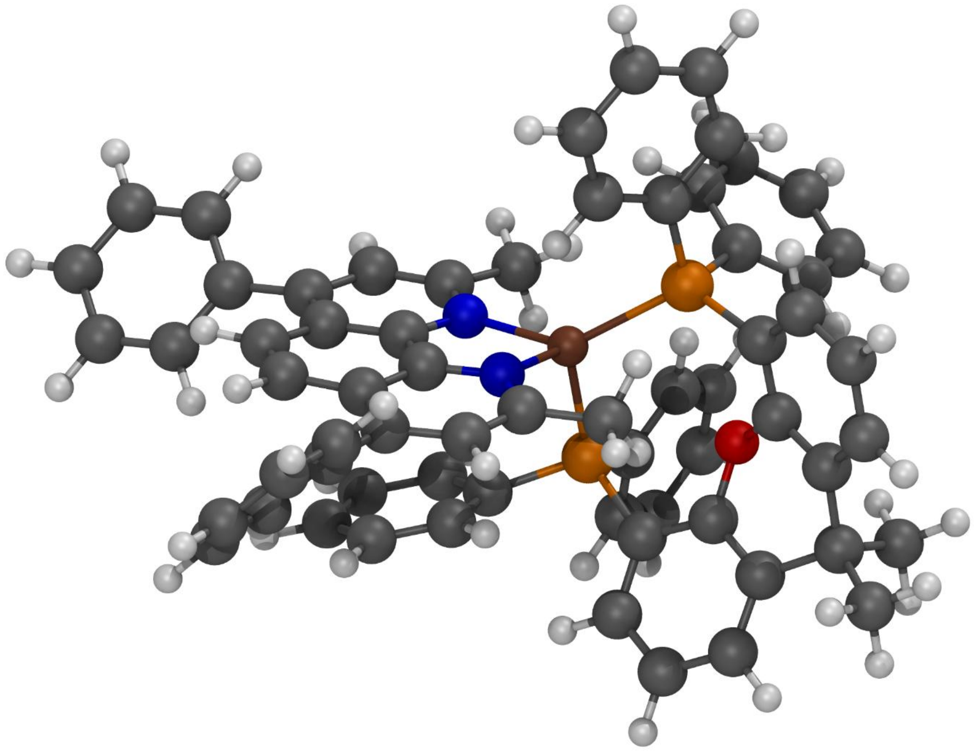

Figure S21. Structure of the geometry optimized S_0 ground state **C4-ref** complex (B3-LYP(BJ)/def2 TZVP with COSMO).

Table S3. Cartesian coordinates of the optimized S_0 **C4-ref** geometry in Å (B3-LYP(BJ)/def2-TZVP with COSMO).

Atom	x	y	z
C	-4.18736	1.60067	2.42793
C	-3.56841	0.86651	1.41718
C	-3.80453	1.25845	0.10014
C	-4.61988	2.33034	-0.24720
C	-5.20568	3.04809	0.79062
C	-4.99414	2.68549	2.11569
C	-3.54313	2.30831	-2.45569
C	-2.76769	1.25006	-1.99518
C	-1.57033	0.85846	-2.59352
C	-1.16468	1.54132	-3.73907
C	-1.91733	2.60064	-4.22525
C	-3.08839	2.98725	-3.58193
H	-4.02217	1.34052	3.46313
H	-5.84085	3.89447	0.57216
H	-5.46173	3.25276	2.90976
H	-0.25095	1.25796	-4.24199
H	-1.59066	3.13121	-5.11011
H	-3.65451	3.81842	-3.97734
C	-5.92112	1.60030	-2.24163
H	-6.09278	1.75022	-3.30896
H	-5.60518	0.56806	-2.08881
H	-6.86218	1.75501	-1.71094
C	-5.34483	4.02567	-1.98545
H	-5.52425	4.18505	-3.04816
H	-4.62443	4.76918	-1.64070
H	-6.29260	4.20050	-1.47738
C	-4.85263	2.59999	-1.73086
O	-3.17550	0.53783	-0.88958
P	-0.64935	-0.49590	-1.79269
P	-2.38307	-0.48941	1.74112
C	-1.37309	-1.97323	-2.57614
C	-2.06935	-2.88362	-1.78589
C	-1.29065	-2.18537	-3.95530
C	-2.67198	-3.99832	-2.35942
H	-2.15253	-2.71817	-0.72321
C	-1.88571	-3.29980	-4.52727
H	-0.75966	-1.48209	-4.58391
C	-2.57766	-4.20913	-3.72876
H	-3.21491	-4.69527	-1.73478
H	-1.81479	-3.46010	-5.59567
H	-3.04362	-5.07702	-4.17814
C	1.04946	-0.39616	-2.43248
C	1.73047	0.82497	-2.43323
C	1.76967	-1.56655	-2.68177
C	3.09094	0.87451	-2.69981
H	1.20195	1.74343	-2.21651
C	3.13434	-1.51629	-2.93469
H	1.26765	-2.52447	-2.67382
C	3.79884	-0.29586	-2.94761
H	3.60004	1.82954	-2.70752
H	3.67717	-2.43394	-3.12201
H	4.86119	-0.25430	-3.15095
C	-2.11944	-0.42362	3.54099
C	-0.91878	0.11934	4.00038
C	-3.06304	-0.87654	4.46626
C	-0.66854	0.22260	5.36384
H	-0.17560	0.45362	3.28726
C	-2.80982	-0.78023	5.82769
H	-3.99443	-1.30874	4.12488
C	-1.61362	-0.22786	6.27833
H	0.26606	0.64513	5.71017
H	-3.54516	-1.13548	6.53838
H	-1.41789	-0.15517	7.34065
C	-3.38461	-1.99846	1.50485
C	-4.53039	-2.01574	0.71085
C	-2.92739	-3.19827	2.05783
C	-5.19842	-3.20946	0.46399
H	-4.91046	-1.10147	0.27938

C	-3.59618	-4.38917	1.81328
H	-2.05225	-3.20406	2.69444
C	-4.73233	-4.39900	1.00960
H	-6.08581	-3.20716	-0.15610
H	-3.23255	-5.30923	2.25298
H	-5.25335	-5.32794	0.81612
Cu	-0.48755	-0.34615	0.50886
C	2.13944	0.88742	0.75740
C	0.56137	2.58012	0.76020
C	2.41018	-0.52791	0.73321
C	3.21464	1.80163	0.73902
C	1.57461	3.54631	0.73265
C	3.74217	-0.98089	0.65470
C	4.54892	1.30096	0.77348
C	2.90481	3.18421	0.70400
H	1.29978	4.59134	0.69830
C	3.95995	-2.37985	0.55067
C	4.80118	-0.02923	0.72510
C	1.56389	-2.67844	0.71056
H	5.36856	1.99985	0.84596
C	2.85911	-3.20393	0.59746
H	5.82071	-0.38288	0.75833
H	2.98163	-4.27547	0.51490
N	0.84438	1.28162	0.76433
N	1.35038	-1.37140	0.76725
C	-0.87589	2.99186	0.74680
H	-1.41556	2.54276	1.57955
H	-0.97512	4.07378	0.80911
H	-1.35626	2.65661	-0.17289
C	0.38700	-3.59932	0.72533
H	0.53838	-4.41423	1.43513
H	-0.51571	-3.05822	0.98869
H	0.24057	-4.04812	-0.26007
C	3.94479	4.23144	0.61980
C	3.95628	5.28071	1.54208
C	4.89789	4.21973	-0.40254
C	4.90828	6.28843	1.45253
C	5.84140	5.23376	-0.49853
C	5.85250	6.26843	0.43156
C	5.30648	-2.96442	0.37946
C	6.15133	-2.53481	-0.64805
C	5.73478	-3.99626	1.21839
C	7.39515	-3.12376	-0.83101
C	6.98415	-4.57733	1.04171
C	7.81719	-4.14384	0.01579
H	8.03471	-2.78818	-1.63749
H	4.91280	7.08910	2.18108
H	6.56665	5.21777	-1.30205
H	8.78934	-4.59894	-0.12406
H	6.59188	7.05570	0.35945
H	7.30751	-5.36812	1.70655
H	3.22637	5.29703	2.34154
H	4.88945	3.42447	-1.13621
H	5.82419	-1.75053	-1.31667
H	5.09229	-4.33234	2.02244

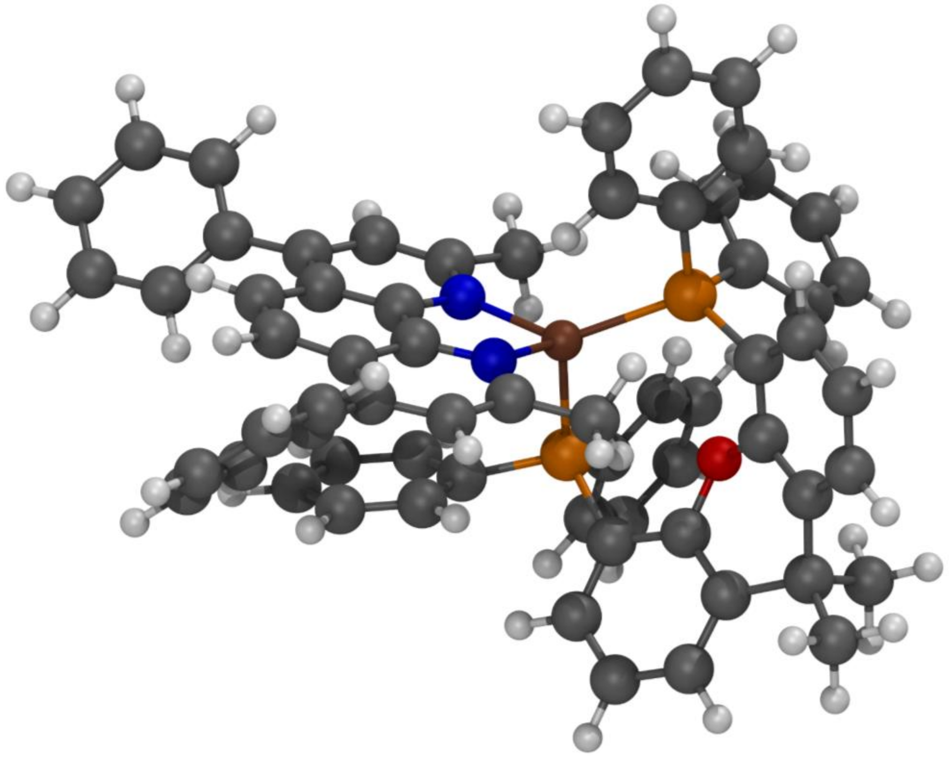

Figure S22. Structure of the geometry optimized T_1 excited state **C4-ref** complex (B3-LYP(BJ)/def2 TZVP with COSMO).

Table S4. Cartesian coordinates of the optimized T_1 **C4-ref** geometry in Å (B3-LYP(BJ)/def2-TZVP with COSMO).

Atom	x	y	z
C	-4.13342	2.17561	2.12055
C	-3.54526	1.22547	1.28874
C	-3.86203	1.25285	-0.06435
C	-4.71233	2.19043	-0.63432
C	-5.27221	3.13492	0.22116
C	-4.99394	3.12267	1.58405
C	-3.69647	1.62422	-2.79635
C	-2.89768	0.71281	-2.11468
C	-1.72563	0.17678	-2.63853
C	-1.35212	0.55817	-3.92764
C	-2.12409	1.46895	-4.63220
C	-3.27884	2.00246	-4.06821
H	-3.90246	2.19378	3.17580
H	-5.93811	3.89026	-0.17066
H	-5.44510	3.86390	2.23013
H	-0.45457	0.15554	-4.37408
H	-1.82627	1.76706	-5.62869
H	-3.86111	2.71147	-4.63900
C	-6.06188	0.98551	-2.34718
H	-6.26113	0.86466	-3.41323
H	-5.73392	0.02438	-1.95019
H	-6.99071	1.26584	-1.84757
C	-5.50075	3.40245	-2.71285
H	-5.71281	3.29452	-3.77582
H	-4.77622	4.20797	-2.58377
H	-6.43440	3.69639	-2.23451
C	-4.99040	2.08372	-2.12973
O	-3.25653	0.30042	-0.85155
P	-0.80213	-1.02640	-1.63231
P	-2.29514	0.03287	1.83698
C	-1.58946	-2.60532	-2.05298
C	-2.35658	-3.26537	-1.09606
C	-1.50054	-3.12457	-3.34779
C	-3.02449	-4.43903	-1.42397

H	-2.43768	-2.86577	-0.09744
C	-2.16092	-4.30071	-3.66996
H	-0.91720	-2.61546	-4.10366
C	-2.92384	-4.95921	-2.70809
H	-3.61992	-4.94426	-0.67537
H	-2.08444	-4.70287	-4.67195
H	-3.43974	-5.87605	-2.96331
C	0.87380	-1.03272	-2.31790
C	1.52842	0.18894	-2.50571
C	1.59558	-2.22044	-2.44869
C	2.87452	0.21857	-2.83475
H	0.99465	1.12095	-2.38099
C	2.94540	-2.18539	-2.76928
H	1.11251	-3.17482	-2.29304
C	3.58716	-0.96776	-2.96329
H	3.36958	1.17016	-2.97564
H	3.49730	-3.11174	-2.86013
H	4.64029	-0.94269	-3.21116
C	-1.85184	0.52369	3.52570
C	-0.64283	1.18582	3.74628
C	-2.70459	0.26650	4.60348
C	-0.29544	1.59675	5.02761
H	0.03153	1.37349	2.92182
C	-2.35360	0.67764	5.88094
H	-3.63871	-0.25634	4.44746
C	-1.14965	1.34423	6.09416
H	0.64474	2.10704	5.19170
H	-3.01819	0.47706	6.71134
H	-0.87706	1.66041	7.09291
C	-3.18743	-1.53625	2.03563
C	-4.41937	-1.77330	1.42710
C	-2.57629	-2.55871	2.76794
C	-5.02552	-3.01828	1.54017
H	-4.91405	-0.99264	0.86814
C	-3.18369	-3.80082	2.87839
H	-1.63374	-2.38143	3.26960
C	-4.40786	-4.03479	2.25925
H	-5.98270	-3.19214	1.06600
H	-2.70402	-4.58430	3.45051
H	-4.88155	-5.00421	2.34477
Cu	-0.32767	-0.26873	0.58447
C	2.18400	1.01310	0.54576
C	0.54913	2.68869	0.36599
C	2.46851	-0.35293	0.66343
C	3.23166	1.95629	0.35925
C	1.52803	3.63894	0.15342
C	3.80549	-0.83089	0.64919
C	4.57281	1.46762	0.44000
C	2.89529	3.29928	0.13862
H	1.22859	4.66031	-0.03266
C	4.02907	-2.21171	0.75054
C	4.84482	0.14762	0.58677
C	1.62388	-2.53674	0.89612
H	5.38731	2.17587	0.40510
C	2.90629	-3.04361	0.92609
H	5.87116	-0.17561	0.67935
H	3.03757	-4.11275	1.01342
N	0.85208	1.38980	0.56740
N	1.39450	-1.21660	0.75972
C	-0.89367	3.08095	0.35381
H	-1.35996	2.90777	1.32403
H	-1.00458	4.13600	0.11186
H	-1.44530	2.50097	-0.38664
C	0.44872	-3.45942	0.95093
H	0.71283	-4.39147	1.44844
H	-0.38790	-3.00871	1.48005
H	0.10432	-3.71030	-0.05388
C	3.89314	4.35221	-0.12106
C	3.82838	5.56691	0.57270
C	4.89034	4.19534	-1.09153
C	4.74282	6.58088	0.32142
C	5.80068	5.21207	-1.34787
C	5.73453	6.40758	-0.63940

C	5.36404	-2.82409	0.65629
C	6.25282	-2.48511	-0.37214
C	5.75360	-3.81198	1.56997
C	7.49265	-3.10145	-0.47384
C	6.99634	-4.42270	1.47250
C	7.87259	-4.06972	0.45038
H	8.16123	-2.83048	-1.28145
H	4.68354	7.50733	0.87889
H	6.55893	5.07335	-2.10841
H	8.84056	-4.54805	0.37251
H	6.44602	7.19899	-0.83767
H	7.28357	-5.17362	2.19788
H	3.06574	5.70695	1.32846
H	4.93824	3.27938	-1.66541
H	5.95845	-1.75116	-1.10949
H	5.08308	-4.08620	2.37460

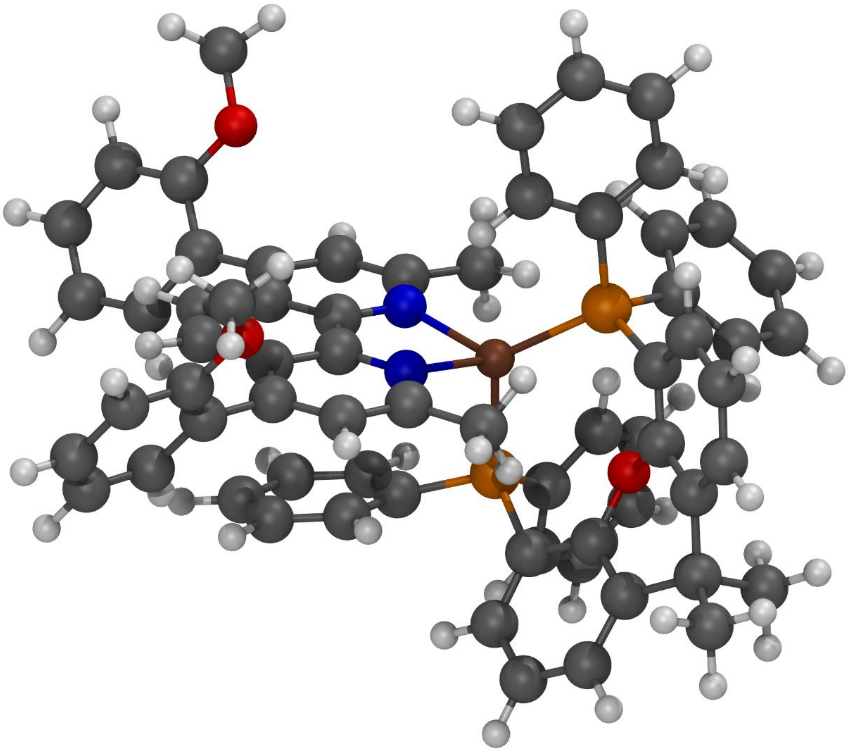

Figure S23. Structure of the geometry optimized S_0 ground state **C1-o** complex (B3-LYP(BJ)/def2 TZVP with COSMO).

Table S5. Cartesian coordinates of the optimized S_0 **C1-o** geometry in Å (B3-LYP(BJ)/def2-TZVP with COSMO).

Atom	x	y	z
C	-4.23752	1.57483	2.59633
C	-3.70205	0.80545	1.56431
C	-4.08679	1.11926	0.26141
C	-4.96829	2.14854	-0.05163
C	-5.46735	2.90399	1.00459
C	-5.10829	2.61831	2.31666
C	-4.12381	2.03957	-2.35636
C	-3.27206	1.02773	-1.92751
C	-2.12996	0.63806	-2.62659
C	-1.86414	1.27247	-3.83893
C	-2.69588	2.28483	-4.29567
C	-3.80793	2.67180	-3.55503
H	-3.95737	1.37504	3.62015
H	-6.14933	3.71961	0.81179
H	-5.51022	3.21361	3.12604
H	-0.99691	0.98885	-4.41834

H	-2.47756	2.77831	-5.23376
H	-4.43779	3.46577	-3.92990
C	-6.44300	1.27890	-1.86083
H	-6.72776	1.36706	-2.91073
H	-6.07947	0.26532	-1.69002
H	-7.32939	1.43593	-1.24368
C	-5.92284	3.72936	-1.78705
H	-6.21493	3.82725	-2.83201
H	-5.19535	4.50925	-1.55639
H	-6.81879	3.90507	-1.19259
C	-5.36100	2.33301	-1.51450
O	-3.54162	0.36387	-0.75159
P	-1.09120	-0.64990	-1.86103
P	-2.44399	-0.49674	1.83040
C	-1.85117	-2.18053	-2.49444
C	-2.44754	-3.05988	-1.59491
C	-1.89793	-2.46341	-3.86250
C	-3.07842	-4.21303	-2.04968
H	-2.43076	-2.84054	-0.53889
C	-2.52068	-3.61639	-4.31650
H	-1.44604	-1.78502	-4.57480
C	-3.11223	-4.49396	-3.40925
H	-3.54289	-4.88536	-1.34051
H	-2.54972	-3.83142	-5.37722
H	-3.60027	-5.39207	-3.76638
C	0.52939	-0.54241	-2.67699
C	1.17323	0.69259	-2.80082
C	1.25260	-1.70668	-2.94502
C	2.49886	0.75986	-3.20378
H	0.64422	1.60800	-2.57259
C	2.58328	-1.63759	-3.33649
H	0.78035	-2.67427	-2.84318
C	3.21021	-0.40457	-3.46934
H	2.97929	1.72434	-3.30429
H	3.12864	-2.55086	-3.53734
H	4.24607	-0.34823	-3.77835
C	-1.99441	-0.31949	3.58574
C	-0.77639	0.29349	3.88282
C	-2.81405	-0.75300	4.63052
C	-0.38794	0.48488	5.20366
H	-0.12828	0.61368	3.07669
C	-2.42234	-0.56886	5.94954
H	-3.75696	-1.23836	4.41562
C	-1.21033	0.05304	6.23793
H	0.55886	0.96155	5.42346
H	-3.06226	-0.90965	6.75355
H	-0.90657	0.19437	7.26745
C	-3.41369	-2.04352	1.78425
C	-4.63959	-2.13550	1.12687
C	-2.85603	-3.19882	2.33970
C	-5.28917	-3.35947	1.01581
H	-5.09669	-1.25640	0.69692
C	-3.50651	-4.41961	2.23159
H	-1.91463	-3.14571	2.87093
C	-4.72422	-4.50458	1.56305
H	-6.23955	-3.41553	0.50043
H	-3.06430	-5.30448	2.67154
H	-5.23094	-5.45715	1.47538
Cu	-0.69464	-0.37516	0.39838
C	1.90300	0.93176	0.33432
C	0.28567	2.58596	0.39355
C	2.21241	-0.47559	0.35397
C	2.94518	1.87005	0.17489
C	1.26486	3.57316	0.23469
C	3.54619	-0.89708	0.18171
C	4.29143	1.40676	0.09146
C	2.59553	3.23936	0.10918
H	0.96263	4.60976	0.19250
C	3.79846	-2.29121	0.13703
C	4.57815	0.08297	0.08819
C	1.43567	-2.64697	0.50045
H	5.08920	2.13224	0.03079
C	2.73271	-3.14239	0.31019

H	5.60480	-0.24616	0.02489
H	2.88541	-4.21232	0.28762
N	0.60424	1.29546	0.43810
N	1.18703	-1.34407	0.51838
C	-1.15868	2.96165	0.48707
H	-1.60908	2.55061	1.38980
H	-1.28270	4.04278	0.49700
H	-1.70939	2.56156	-0.36468
C	0.29166	-3.59704	0.65249
H	0.52447	-4.37446	1.38205
H	-0.60259	-3.06879	0.96609
H	0.07846	-4.09407	-0.29699
C	3.60958	4.29118	-0.12824
C	3.83583	5.29925	0.82537
C	4.33616	4.31377	-1.31553
C	4.77360	6.29937	0.57148
C	5.26678	5.31197	-1.57585
C	5.48059	6.30153	-0.62630
C	5.15168	-2.82933	-0.12594
C	5.80875	-2.52705	-1.31583
C	5.78507	-3.67684	0.80112
C	7.06226	-3.05136	-1.60540
C	7.04621	-4.19968	0.51765
C	7.67547	-3.88733	-0.68276
H	7.55060	-2.81020	-2.54013
H	4.95967	7.07501	1.29903
H	5.81442	5.31566	-2.50874
O	3.11365	5.21317	1.97152
C	3.30065	6.20972	2.97342
H	4.32924	6.21020	3.34187
H	3.04673	7.20241	2.59410
H	2.62484	5.94554	3.78276
H	7.54369	-4.84629	1.22469
O	5.10994	-3.91938	1.95334
C	5.70513	-4.77204	2.92817
H	5.87528	-5.77324	2.52511
H	6.64888	-4.35779	3.29088
H	4.99316	-4.82587	3.74778
H	5.31852	-1.87738	-2.02820
H	4.15830	3.53931	-2.05059
H	8.65311	-4.30375	-0.88967
H	6.20344	7.08605	-0.81068

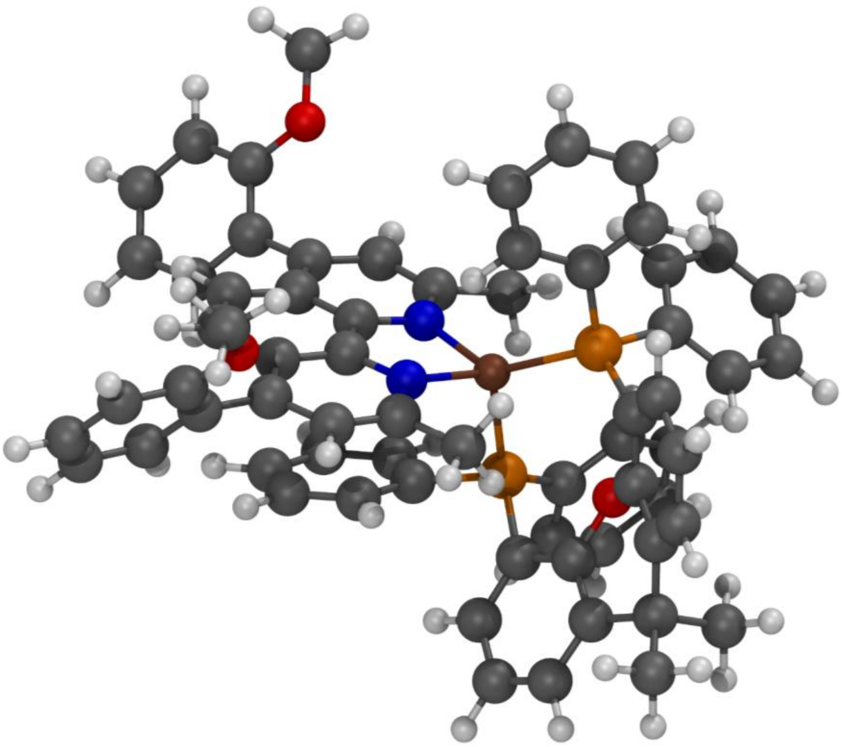

Figure S24. Structure of the geometry optimized T_1 ground state **C1-o** complex (B3-LYP(BJ)/def2 TZVP with COSMO).

Table S6. Cartesian coordinates of the optimized T_1 **C1-o** geometry in Å (B3-LYP(BJ)/def2-TZVP with COSMO).

Atom	x	y	z
C	-4.22463	2.04644	2.33806
C	-3.69908	1.10331	1.45772
C	-4.14031	1.11946	0.13972
C	-5.05497	2.04086	-0.35166
C	-5.54963	2.97923	0.54952
C	-5.14722	2.97668	1.88112
C	-4.22989	1.48626	-2.59604
C	-3.35764	0.58959	-1.98891
C	-2.22857	0.07253	-2.61586
C	-1.97843	0.45934	-3.93283
C	-2.82564	1.35620	-4.56512
C	-3.93454	1.87011	-3.90003
H	-3.89722	2.07283	3.36732
H	-6.26092	3.72234	0.21876
H	-5.54956	3.71274	2.56431
H	-1.11718	0.07212	-4.45766
H	-2.62294	1.65873	-5.58394
H	-4.57762	2.56846	-4.41604
C	-6.53377	0.80843	-1.93357
H	-6.82674	0.68134	-2.97702
H	-6.15480	-0.14594	-1.56686
H	-7.41809	1.07434	-1.35202
C	-6.04964	3.23336	-2.35132
H	-6.35529	3.11913	-3.39066
H	-5.33047	4.05151	-2.28920
H	-6.94110	3.51239	-1.79063
C	-5.46590	1.92513	-1.81547
O	-3.59384	0.17453	-0.69792
P	-1.19832	-1.11428	-1.69651
P	-2.37870	-0.06096	1.88995
C	-1.99654	-2.70491	-2.04862
C	-2.66121	-3.37839	-1.02654
C	-2.02045	-3.22091	-3.34746
C	-3.33873	-4.56172	-1.29439
H	-2.65496	-2.98170	-0.02345

C	-2.68985	-4.40669	-3.61044
H	-1.51773	-2.70165	-4.15271
C	-3.35001	-5.07837	-2.58386
H	-3.85413	-5.07723	-0.49513
H	-2.70078	-4.80603	-4.61643
H	-3.87343	-6.00264	-2.79281
C	0.40810	-1.09231	-2.53232
C	1.02427	0.14112	-2.76806
C	1.13226	-2.26728	-2.73972
C	2.33348	0.19468	-3.21917
H	0.49034	1.06357	-2.58604
C	2.44585	-2.20839	-3.18414
H	0.68007	-3.23035	-2.54879
C	3.04837	-0.97937	-3.42550
H	2.79946	1.15486	-3.39584
H	3.00035	-3.12536	-3.33480
H	4.07299	-0.93491	-3.77104
C	-1.79252	0.44756	3.52939
C	-0.58823	1.14584	3.63364
C	-2.53168	0.16985	4.68318
C	-0.13392	1.57199	4.87592
H	-0.00064	1.34951	2.74884
C	-2.07382	0.59601	5.92137
H	-3.46075	-0.38041	4.61667
C	-0.87547	1.29860	6.01890
H	0.80213	2.11020	4.94973
H	-2.65073	0.37920	6.81114
H	-0.51912	1.62653	6.98703
C	-3.21461	-1.64788	2.17282
C	-4.49520	-1.91098	1.68804
C	-2.51245	-2.65739	2.83833
C	-5.06059	-3.16880	1.85698
H	-5.05885	-1.14079	1.18224
C	-3.07947	-3.91221	3.00514
H	-1.52848	-2.46022	3.24395
C	-4.35353	-4.17210	2.50918
H	-6.05580	-3.36307	1.47869
H	-2.52890	-4.68567	3.52462
H	-4.79561	-5.15159	2.63826
Cu	-0.53052	-0.33716	0.46207
C	1.93829	0.99124	0.21998
C	0.26129	2.63459	0.15561
C	2.25952	-0.36746	0.31414
C	2.94841	1.95591	-0.04722
C	1.20160	3.60309	-0.12808
C	3.60200	-0.81606	0.19724
C	4.30174	1.49451	-0.09772
C	2.56983	3.28940	-0.23465
H	0.87323	4.62143	-0.27407
C	3.86404	-2.18797	0.28793
C	4.61260	0.18130	0.02802
C	1.48615	-2.57208	0.59681
H	5.09147	2.22077	-0.22538
C	2.77775	-3.04888	0.53330
H	5.64801	-0.12719	0.01246
H	2.94471	-4.11157	0.62681
N	0.60575	1.34178	0.34300
N	1.21627	-1.25495	0.48975
C	-1.18663	2.99649	0.24788
H	-1.57545	2.82841	1.25267
H	-1.33737	4.04566	0.00174
H	-1.78050	2.39539	-0.44132
C	0.33897	-3.52126	0.73380
H	0.66561	-4.45806	1.18281
H	-0.45565	-3.10138	1.34694
H	-0.09085	-3.75712	-0.24120
C	3.53975	4.34362	-0.59173
C	3.67999	5.50041	0.20023
C	4.31621	4.23530	-1.74463
C	4.57516	6.50193	-0.17149
C	5.21044	5.23069	-2.12068
C	5.33515	6.36344	-1.32914
C	5.20953	-2.75110	0.06919

C	5.89810	-2.49663	-1.11696
C	5.81700	-3.59146	1.02415
C	7.14761	-3.04851	-1.37127
C	7.07039	-4.14745	0.77445
C	7.72923	-3.87550	-0.42080
H	7.65544	-2.83876	-2.30352
H	4.69157	7.38718	0.43565
H	5.79577	5.12297	-3.02438
O	2.92163	5.55094	1.32686
C	3.01879	6.69806	2.16398
H	4.03107	6.81590	2.55895
H	2.72896	7.60543	1.62797
H	2.32741	6.52529	2.98514
H	7.54284	-4.78587	1.50585
O	5.12542	-3.78914	2.17688
C	5.68992	-4.63095	3.17669
H	5.84106	-5.64595	2.80061
H	6.64004	-4.23153	3.54026
H	4.96922	-4.64875	3.99052
H	5.42932	-1.86341	-1.85784
H	4.20209	3.35506	-2.36426
H	8.70172	-4.31617	-0.60060
H	6.02542	7.15040	-1.60569

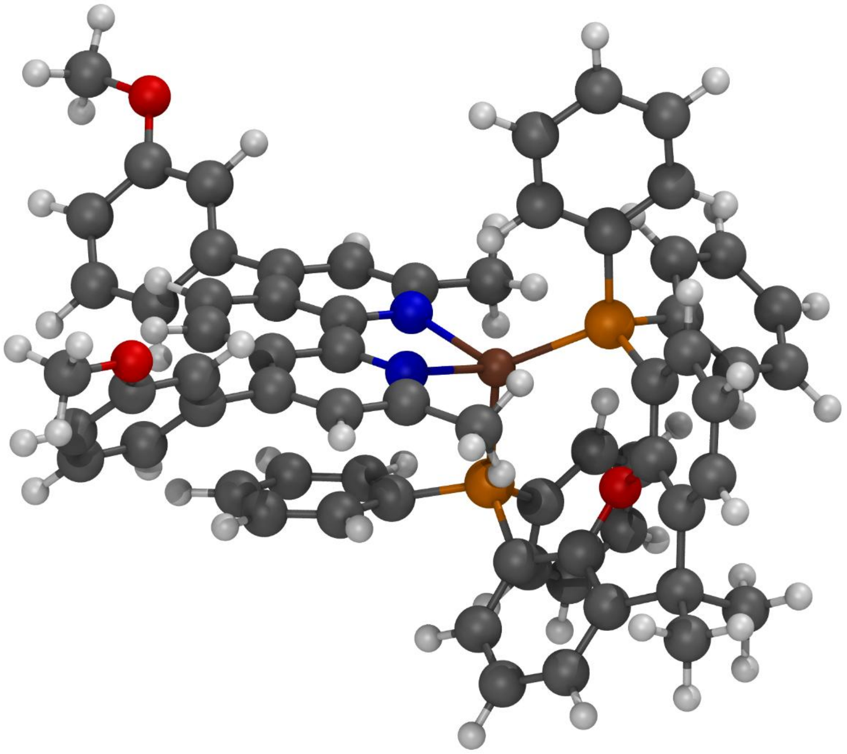

Figure S25. Structure of the geometry optimized S_0 ground state **C2-*m*** complex (B3-LYP(BJ)/def2 TZVP with COSMO).

Table S7. Cartesian coordinates of the optimized S_0 **C2-*m*** geometry in Å (B3-LYP(BJ)/def2-TZVP with COSMO).

Atom	x	y	z
C	-4.41859	1.52756	2.59775
C	-3.86189	0.76539	1.57174
C	-4.21562	1.09141	0.26304
C	-5.08748	2.12521	-0.06150
C	-5.60850	2.87316	0.98955
C	-5.27994	2.57585	2.30695
C	-4.19295	2.03137	-2.34820
C	-3.35246	1.01487	-1.90829
C	-2.19667	0.62718	-2.58588
C	-1.90499	1.26805	-3.78887

C	-2.72505	2.28499	-4.25641
C	-3.85096	2.67009	-3.53623
H	-4.16220	1.31864	3.62595
H	-6.28382	3.69228	0.78820
H	-5.69852	3.16570	3.11185
H	-1.02693	0.98593	-4.35238
H	-2.48664	2.78344	-5.18693
H	-4.47117	3.46793	-3.91889
C	-6.52458	1.27451	-1.90931
H	-6.78618	1.37172	-2.96441
H	-6.16781	0.25841	-1.73885
H	-7.42363	1.42930	-1.31025
C	-5.99860	3.72268	-1.80490
H	-6.26783	3.82990	-2.85512
H	-5.27395	4.49837	-1.55225
H	-6.90679	3.89653	-1.22879
C	-5.44722	2.32234	-1.53115
O	-3.64790	0.34343	-0.74317
P	-1.17550	-0.66660	-1.80657
P	-2.61387	-0.54298	1.85451
C	-1.91888	-2.19468	-2.46452
C	-2.51846	-3.08828	-1.58130
C	-1.94640	-2.46356	-3.83593
C	-3.13385	-4.24197	-2.05568
H	-2.51609	-2.87968	-0.52300
C	-2.55412	-3.61675	-4.30933
H	-1.49145	-1.77412	-4.53553
C	-3.14904	-4.50870	-3.41840
H	-3.60109	-4.92554	-1.35920
H	-2.56863	-3.82100	-5.37248
H	-3.62516	-5.40701	-3.79079
C	0.46249	-0.55443	-2.58688
C	1.10685	0.68135	-2.69801
C	1.19375	-1.71781	-2.83746
C	2.44101	0.75083	-3.07189
H	0.57134	1.59622	-2.48304
C	2.53309	-1.64682	-3.19770
H	0.72062	-2.68604	-2.74576
C	3.16034	-0.41265	-3.31874
H	2.92122	1.71643	-3.16317
H	3.08587	-2.55897	-3.38259
H	4.20285	-0.35543	-3.60420
C	-2.20495	-0.38558	3.62136
C	-0.98926	0.21414	3.95310
C	-3.05325	-0.82087	4.64233
C	-0.63113	0.39079	5.28450
H	-0.31914	0.53549	3.16557
C	-2.69195	-0.65133	5.97190
H	-3.99481	-1.29618	4.40065
C	-1.48197	-0.04257	6.29480
H	0.31404	0.85736	5.53134
H	-3.35400	-0.99336	6.75724
H	-1.20194	0.08732	7.33249
C	-3.58606	-2.08674	1.76876
C	-4.78853	-2.17123	1.06824
C	-3.05364	-3.24647	2.33952
C	-5.43907	-3.39177	0.93018
H	-5.22657	-1.28892	0.62546
C	-3.70492	-4.46416	2.20376
H	-2.13233	-3.19948	2.90542
C	-4.89861	-4.54138	1.49254
H	-6.37105	-3.44180	0.38165
H	-3.28244	-5.35231	2.65627
H	-5.40620	-5.49130	1.38377
Cu	-0.83060	-0.40713	0.46434
C	1.76971	0.90455	0.44225
C	0.14813	2.55492	0.49448
C	2.07918	-0.50265	0.46252
C	2.81288	1.84361	0.29457
C	1.12762	3.54413	0.34046
C	3.41412	-0.92429	0.30172
C	4.15997	1.38020	0.24343
C	2.46073	3.21416	0.22151

H	0.82172	4.57939	0.28190
C	3.66456	-2.32034	0.25134
C	4.44695	0.05620	0.23963
C	1.29784	-2.67291	0.60758
H	4.96043	2.10391	0.21178
C	2.59612	-3.16972	0.42221
H	5.47538	-0.27046	0.20451
H	2.74532	-4.24035	0.38512
N	0.46824	1.26584	0.53571
N	1.05065	-1.37069	0.61852
C	-1.29681	2.92958	0.57921
H	-1.75732	2.50003	1.46787
H	-1.42102	4.01032	0.60892
H	-1.83761	2.54697	-0.28689
C	0.15275	-3.62192	0.75320
H	0.39216	-4.41834	1.45940
H	-0.73505	-3.09774	1.09136
H	-0.07551	-4.09376	-0.20575
C	3.46253	4.28065	0.00467
C	3.52178	5.35458	0.88437
C	4.32002	4.24751	-1.10169
C	4.43620	6.39048	0.68135
C	5.21582	5.28400	-1.30526
H	4.27039	3.42690	-1.80386
C	5.28894	6.35749	-0.42132
H	5.87143	5.26554	-2.16662
H	6.00123	7.14876	-0.60036
C	5.01282	-2.87642	0.00685
C	5.75803	-2.48687	-1.11280
C	5.52783	-3.83511	0.87114
C	6.99658	-3.06137	-1.34501
H	5.35824	-1.75669	-1.80150
C	6.78244	-4.40246	0.63834
C	7.52309	-4.01525	-0.47774
H	7.56908	-2.76935	-2.21633
H	8.49380	-4.44245	-0.67969
H	4.97349	-4.14568	1.74723
H	2.87311	5.39801	1.74961
O	4.41925	7.38115	1.60908
O	7.19361	-5.31824	1.55235
C	5.33095	8.46811	1.45872
H	5.14521	9.12983	2.30069
H	6.36619	8.11987	1.48874
H	5.15127	9.00577	0.52464
C	8.46812	-5.93289	1.37125
H	8.58844	-6.61835	2.20609
H	8.50618	-6.49034	0.43221
H	9.26907	-5.18989	1.38978

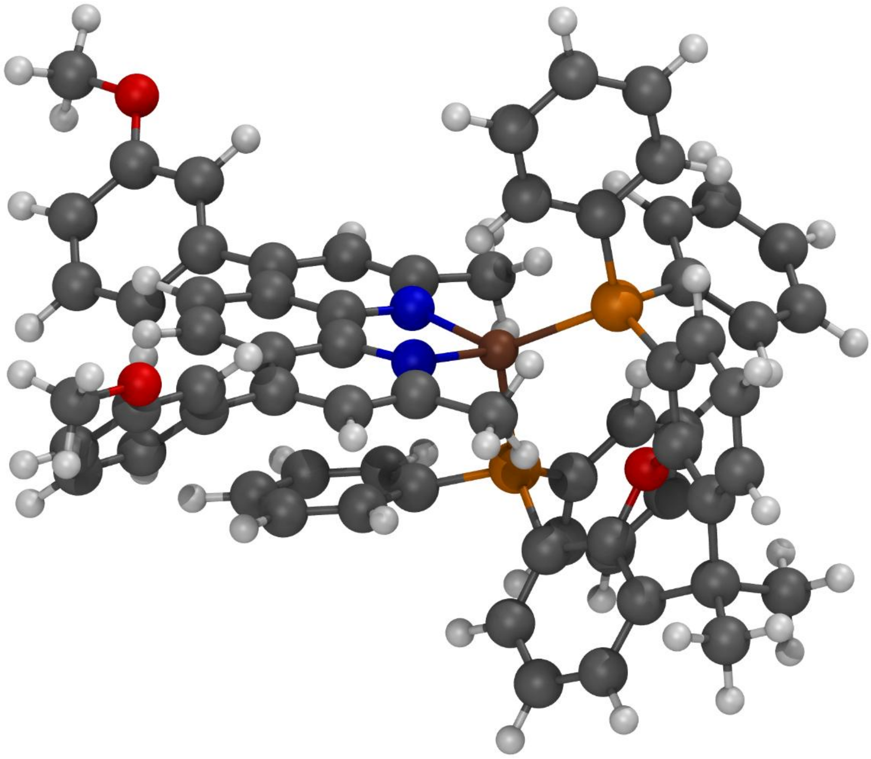

Figure S26. Structure of the geometry optimized T_1 ground state **C2-m** complex (B3-LYP(BJ)/def2 TZVP with COSMO).

Table S8. Cartesian coordinates of the optimized T_1 **C2-m** geometry in Å (B3-LYP(BJ)/def2-TZVP with COSMO).

Atom	x	y	z
C	-4.43115	1.88641	2.38221
C	-3.87066	0.98267	1.48237
C	-4.27458	1.04444	0.15391
C	-5.18672	1.97321	-0.32819
C	-5.71705	2.87165	0.59316
C	-5.35119	2.82358	1.93442
C	-4.29501	1.51175	-2.56813
C	-3.42748	0.60419	-1.97053
C	-2.27526	0.12446	-2.58504
C	-1.99599	0.56122	-3.88049
C	-2.83819	1.46979	-4.50259
C	-3.97052	1.94613	-3.84913
H	-4.13315	1.87730	3.42065
H	-6.42792	3.61876	0.27066
H	-5.78056	3.52930	2.63298
H	-1.11677	0.20334	-4.39616
H	-2.61307	1.81104	-5.50431
H	-4.60888	2.65493	-4.35666
C	-6.60674	0.78259	-1.99358
H	-6.87017	0.69064	-3.04852
H	-6.22545	-0.18014	-1.65193
H	-7.50955	1.01631	-1.42683
C	-6.14305	3.22688	-2.30920
H	-6.41966	3.14730	-3.35984
H	-5.43643	4.05075	-2.19801
H	-7.05252	3.47439	-1.76284
C	-5.55690	1.90687	-1.80587
O	-3.69297	0.13826	-0.70292
P	-1.25386	-1.08164	-1.68194
P	-2.55280	-0.18659	1.90847
C	-2.01951	-2.66982	-2.10822
C	-2.70198	-3.38695	-1.12857
C	-1.99997	-3.14112	-3.42408
C	-3.35434	-4.56967	-1.45530
H	-2.72955	-3.02474	-0.11294

C	-2.64453	-4.32612	-3.74576
H	-1.48265	-2.58785	-4.19678
C	-3.32265	-5.04171	-2.76138
H	-3.88384	-5.11938	-0.68877
H	-2.62196	-4.69077	-4.76464
H	-3.82648	-5.96536	-3.01614
C	0.37373	-1.01232	-2.47224
C	0.97829	0.23484	-2.66017
C	1.12030	-2.17191	-2.68865
C	2.29850	0.31755	-3.07411
H	0.42657	1.14504	-2.46977
C	2.44471	-2.08392	-3.09381
H	0.67684	-3.14551	-2.53398
C	3.03589	-0.84098	-3.28801
H	2.75505	1.28834	-3.21379
H	3.01726	-2.98878	-3.24966
H	4.06956	-0.77476	-3.60143
C	-2.01766	0.26214	3.58218
C	-0.81912	0.95820	3.74879
C	-2.79029	-0.05995	4.70206
C	-0.40338	1.33842	5.01915
H	-0.20585	1.19630	2.89036
C	-2.37114	0.32064	5.96840
H	-3.71521	-0.60922	4.58723
C	-1.17829	1.02125	6.12816
H	0.52826	1.87547	5.14110
H	-2.97393	0.06982	6.83167
H	-0.85227	1.31357	7.11812
C	-3.38268	-1.78960	2.10644
C	-4.64289	-2.04612	1.56763
C	-2.69541	-2.81571	2.76203
C	-5.20218	-3.31351	1.67325
H	-5.19568	-1.26367	1.06884
C	-3.25615	-4.08015	2.86525
H	-1.72888	-2.62468	3.21013
C	-4.50931	-4.33305	2.31541
H	-6.18164	-3.50257	1.25350
H	-2.71735	-4.86646	3.37773
H	-4.94660	-5.31992	2.39511
Cu	-0.66123	-0.38282	0.52592
C	1.80130	0.98165	0.36800
C	0.10752	2.60754	0.35205
C	2.13485	-0.37720	0.42092
C	2.80467	1.96394	0.14346
C	1.04053	3.59545	0.10694
C	3.48191	-0.81026	0.30141
C	4.16267	1.51747	0.11795
C	2.41294	3.30087	-0.00997
H	0.69901	4.61219	-0.02336
C	3.75366	-2.18533	0.34022
C	4.48431	0.20320	0.20220
C	1.37517	-2.59416	0.63352
H	4.95000	2.25349	0.05020
C	2.67159	-3.05908	0.56148
H	5.52363	-0.09013	0.21327
H	2.84190	-4.12536	0.60400
N	0.46279	1.31366	0.48957
N	1.09663	-1.27842	0.55823
C	-1.34400	2.95333	0.44856
H	-1.73957	2.73263	1.44030
H	-1.50192	4.01188	0.25247
H	-1.92614	2.38157	-0.27465
C	0.23538	-3.55648	0.73372
H	0.56168	-4.49605	1.17718
H	-0.57614	-3.15246	1.33481
H	-0.16993	-3.78325	-0.25393
C	3.35938	4.39295	-0.30327
C	3.29885	5.57372	0.43329
C	4.28990	4.29544	-1.34869
C	4.15815	6.63736	0.15668
C	5.13510	5.35708	-1.62455
H	4.32974	3.40149	-1.95522
C	5.08622	6.53272	-0.87895

H	5.84468	5.28003	-2.43895
H	5.75872	7.34401	-1.11342
C	5.09774	-2.75004	0.13249
C	5.90018	-2.34664	-0.94604
C	5.56827	-3.75056	0.98076
C	7.13866	-2.93242	-1.14498
H	5.53835	-1.59822	-1.63586
C	6.82044	-4.33155	0.78070
C	7.61633	-3.92240	-0.28903
H	7.74902	-2.62414	-1.98474
H	8.58709	-4.36049	-0.46551
H	4.97721	-4.08047	1.82519
H	2.59494	5.67889	1.24866
O	4.01764	7.73175	0.95204
O	7.17921	-5.28550	1.68147
C	4.87390	8.84828	0.72497
H	4.59466	9.59321	1.46582
H	5.92325	8.57446	0.86026
H	4.72958	9.25886	-0.27756
C	8.45237	-5.90947	1.53597
H	8.52971	-6.62237	2.35298
H	8.52616	-6.43821	0.58230
H	9.26093	-5.17811	1.61195

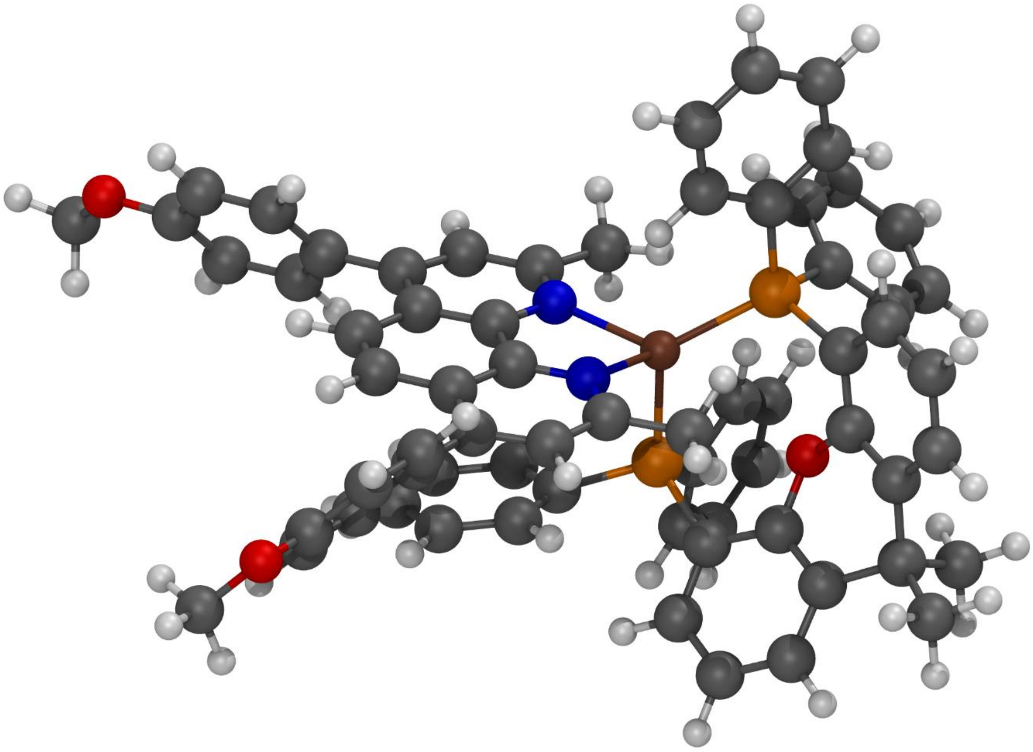

Figure S27. Structure of the geometry optimized S_0 ground state **C3-p** complex (B3-LYP(BJ)/def2 TZVP with COSMO).

Table S9. Cartesian coordinates of the optimized S_0 **C3-p** geometry in Å (B3-LYP(BJ)/def2-TZVP with COSMO).

Atom	x	y	z
C	-4.67287	1.50046	2.46429
C	-4.04836	0.79951	1.43371
C	-4.26367	1.24455	0.12998
C	-5.06084	2.33983	-0.18521
C	-5.65269	3.02302	0.87235
C	-5.46380	2.60571	2.18462
C	-3.95171	2.39261	-2.37719
C	-3.19859	1.30481	-1.95020

C	-2.00006	0.91775	-2.54955
C	-1.56759	1.64095	-3.65977
C	-2.29650	2.73229	-4.11072
C	-3.47074	3.11037	-3.46813
H	-4.52395	1.19837	3.49053
H	-6.27473	3.88514	0.67917
H	-5.93610	3.14649	2.99426
H	-0.65238	1.36279	-4.16295
H	-1.94911	3.29396	-4.96807
H	-4.01877	3.96565	-3.83666
C	-6.34172	1.70966	-2.22659
H	-6.49484	1.90481	-3.28944
H	-6.04197	0.66780	-2.11085
H	-7.28874	1.85607	-1.70418
C	-5.73699	4.11445	-1.86293
H	-5.89782	4.31939	-2.92075
H	-5.01193	4.83298	-1.47736
H	-6.68997	4.28182	-1.36213
C	-5.26793	2.67277	-1.65982
O	-3.63157	0.55493	-0.87952
P	-1.12020	-0.48928	-1.79504
P	-2.88064	-0.58025	1.72056
C	-1.88531	-1.91262	-2.63890
C	-2.60451	-2.83905	-1.88921
C	-1.80773	-2.06587	-4.02615
C	-3.23500	-3.91175	-2.51108
H	-2.68323	-2.71923	-0.82019
C	-2.43083	-3.13824	-4.64655
H	-1.25873	-1.34908	-4.62334
C	-3.14590	-4.06413	-3.88846
H	-3.79535	-4.62175	-1.91735
H	-2.36374	-3.25276	-5.72109
H	-3.63374	-4.89919	-4.37533
C	0.58384	-0.42469	-2.42404
C	1.30768	0.77073	-2.38510
C	1.26553	-1.61314	-2.69674
C	2.67304	0.77741	-2.63148
H	0.80992	1.70170	-2.14931
C	2.63506	-1.60572	-2.92611
H	0.72920	-2.55198	-2.72236
C	3.34394	-0.41105	-2.89494
H	3.21572	1.71328	-2.60585
H	3.14836	-2.53664	-3.12992
H	4.41188	-0.40609	-3.07009
C	-2.63281	-0.57811	3.52418
C	-1.43362	-0.05706	4.01209
C	-3.58635	-1.05787	4.42528
C	-1.19497	-0.00142	5.38034
H	-0.68255	0.29723	3.31712
C	-3.34456	-1.00936	5.79140
H	-4.51668	-1.47372	4.06143
C	-2.15001	-0.47827	6.27089
H	-0.26154	0.40462	5.74886
H	-4.08766	-1.38532	6.48315
H	-1.96323	-0.44274	7.33671
C	-3.89926	-2.06725	1.42612
C	-5.04824	-2.03971	0.63720
C	-3.45327	-3.29322	1.92849
C	-5.73107	-3.21504	0.34609
H	-5.41919	-1.10452	0.24414
C	-4.13684	-4.46569	1.64008
H	-2.57503	-3.33415	2.55964
C	-5.27657	-4.43050	0.84205
H	-6.62071	-3.17801	-0.26965
H	-3.78190	-5.40668	2.04088
H	-5.80905	-5.34503	0.61415
Cu	-0.97075	-0.41573	0.51361
C	1.66698	0.78696	0.79831
C	0.10253	2.49045	0.86462
C	1.92585	-0.63041	0.75155
C	2.74793	1.69464	0.76653
C	1.12122	3.44946	0.83289
C	3.25425	-1.09606	0.68017

C	4.07727	1.18303	0.73211
C	2.44927	3.08206	0.75752
H	0.85137	4.49631	0.82310
C	3.46170	-2.49798	0.57603
C	4.31872	-0.14909	0.67706
C	1.05621	-2.76763	0.61967
H	4.90490	1.87554	0.74129
C	2.34778	-3.30539	0.53121
H	5.33602	-0.50430	0.61817
H	2.46202	-4.37830	0.45524
N	0.37436	1.18970	0.83229
N	0.85630	-1.46309	0.74187
C	-1.33113	2.91434	0.89032
H	-1.86574	2.42628	1.70394
H	-1.41950	3.99236	1.01060
H	-1.82488	2.63357	-0.04050
C	-0.13199	-3.67246	0.56224
H	0.00954	-4.54623	1.19994
H	-1.02823	-3.14448	0.87133
H	-0.28567	-4.03403	-0.45755
C	3.48758	4.12549	0.65577
C	3.50617	5.19363	1.56196
C	4.43492	4.11841	-0.36728
C	4.44570	6.20098	1.45950
C	5.37720	5.13141	-0.49277
C	5.39057	6.17900	0.42887
C	4.80635	-3.10082	0.49261
C	5.78532	-2.86037	1.46544
C	5.11780	-3.97092	-0.55019
C	7.02511	-3.46457	1.39224
C	6.36557	-4.57625	-0.64549
C	7.32876	-4.32391	0.33116
H	7.77474	-3.28866	2.15272
H	4.46690	7.01641	2.17068
H	6.08456	5.09621	-1.30764
O	8.57277	-4.86206	0.33793
H	6.57229	-5.23352	-1.47674
H	5.56421	-2.21193	2.30324
H	4.42997	3.32250	-1.10001
O	6.27011	7.20922	0.40399
C	7.25905	7.23678	-0.62414
C	8.93457	-5.75393	-0.71514
H	9.95684	-6.05907	-0.50784
H	8.89186	-5.25369	-1.68567
H	8.28545	-6.63282	-0.72586
H	7.84679	8.13350	-0.44660
H	6.79654	7.29237	-1.61250
H	7.90631	6.35819	-0.56978
H	4.37926	-4.17155	-1.31587
H	2.78490	5.22275	2.36887

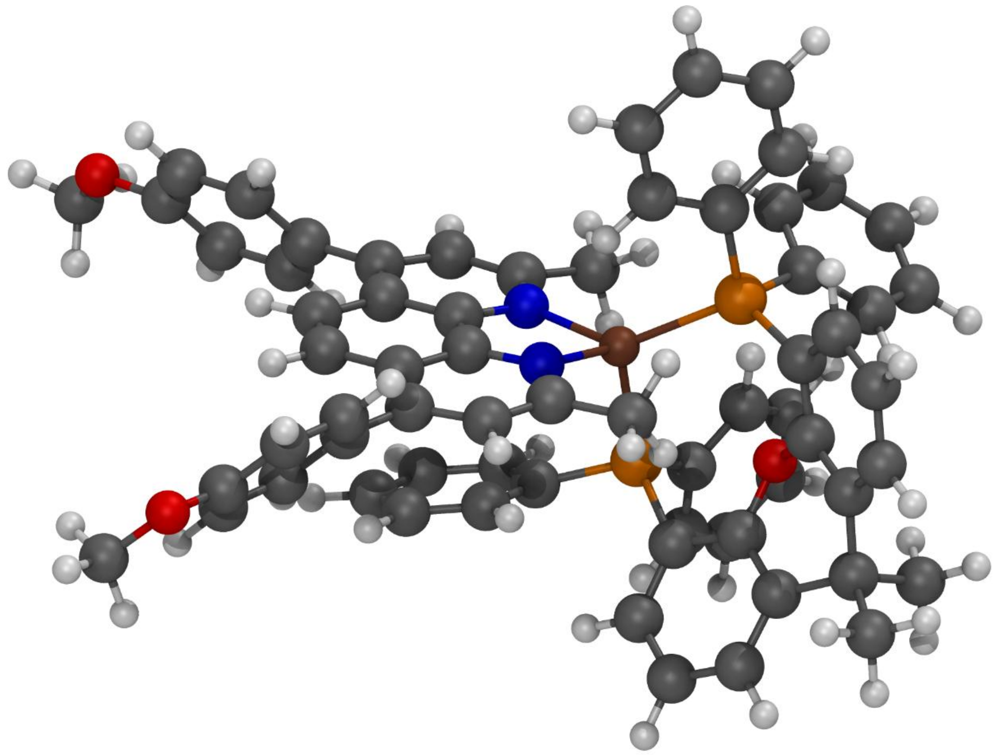

Figure S28. Structure of the geometry optimized T_1 ground state **C3-p** complex (B3-LYP(BJ)/def2 TZVP with COSMO).

Table S10. Cartesian coordinates of the optimized T_1 **C3-p** geometry in Å (B3-LYP(BJ)/def2-TZVP with COSMO).

Atom	x	y	z
C	-4.60605	1.89320	2.34423
C	-4.01931	1.04257	1.40996
C	-4.31299	1.24273	0.06626
C	-5.13293	2.26317	-0.39590
C	-5.69166	3.10479	0.56154
C	-5.44050	2.91597	1.91665
C	-4.07378	1.96018	-2.58878
C	-3.31222	0.94833	-2.01544
C	-2.13913	0.45602	-2.57870
C	-1.72079	0.99859	-3.79394
C	-2.45302	2.01534	-4.38780
C	-3.61283	2.49529	-3.78740
H	-4.39251	1.77693	3.39688
H	-6.33434	3.91799	0.25623
H	-5.89026	3.57970	2.64300
H	-0.82033	0.63669	-4.26856
H	-2.12033	2.43791	-5.32659
H	-4.16435	3.28788	-4.27230
C	-6.46084	1.31711	-2.28077
H	-6.63466	1.33875	-3.35785
H	-6.16043	0.30629	-2.00351
H	-7.39709	1.55017	-1.77083
C	-5.84893	3.74878	-2.31705
H	-6.03533	3.78343	-3.38980
H	-5.11444	4.51611	-2.06772
H	-6.78943	3.99710	-1.82644
C	-5.37634	2.35575	-1.89832
O	-3.71122	0.38372	-0.82493
P	-1.28060	-0.89866	-1.71854
P	-2.78797	-0.22180	1.82450
C	-2.13404	-2.37787	-2.33247
C	-2.87484	-3.16585	-1.45558
C	-2.10922	-2.69344	-3.69423
C	-3.57962	-4.26594	-1.92998
H	-2.90765	-2.92327	-0.40530

C	-2.80739	-3.79543	-4.16406
H	-1.54597	-2.08279	-4.38767
C	-3.54357	-4.58329	-3.28167
H	-4.15441	-4.87154	-1.24234
H	-2.78125	-4.03906	-5.21837
H	-4.08941	-5.44190	-3.65148
C	0.39882	-0.91015	-2.39538
C	1.12773	0.28376	-2.41247
C	1.04992	-2.11055	-2.68407
C	2.47906	0.27451	-2.71927
H	0.64783	1.22141	-2.16784
C	2.40574	-2.11538	-2.98103
H	0.50674	-3.04503	-2.66907
C	3.12415	-0.92570	-2.99526
H	3.03272	1.20373	-2.72253
H	2.90208	-3.05271	-3.19580
H	4.18380	-0.93420	-3.21456
C	-2.36156	0.05577	3.56558
C	-1.14646	0.66715	3.87932
C	-3.23261	-0.31672	4.59391
C	-0.81124	0.91451	5.20515
H	-0.45816	0.94258	3.09178
C	-2.89383	-0.06856	5.91595
H	-4.17167	-0.80243	4.36448
C	-1.68372	0.54849	6.22288
H	0.13372	1.38636	5.44110
H	-3.57273	-0.35764	6.70780
H	-1.42068	0.73742	7.25578
C	-3.69860	-1.79257	1.82329
C	-4.92889	-1.93776	1.18363
C	-3.10583	-2.90362	2.43090
C	-5.55253	-3.17859	1.14414
H	-5.40860	-1.08903	0.71862
C	-3.73039	-4.14139	2.38876
H	-2.16449	-2.80066	2.95513
C	-4.95372	-4.28217	1.74021
H	-6.50835	-3.28124	0.64701
H	-3.26511	-4.99439	2.86536
H	-5.44106	-5.24804	1.70701
Cu	-0.80514	-0.39127	0.57073
C	1.71276	0.86555	0.68695
C	0.08836	2.56308	0.70751
C	1.98948	-0.50700	0.67642
C	2.76360	1.81694	0.56456
C	1.07086	3.52220	0.57757
C	3.32487	-0.99155	0.63668
C	4.10016	1.31152	0.52275
C	2.43545	3.17816	0.48359
H	0.77604	4.55953	0.50900
C	3.53935	-2.37751	0.62578
C	4.36676	-0.01733	0.56048
C	1.12729	-2.69552	0.61932
H	4.91915	2.01243	0.46346
C	2.40577	-3.21275	0.62233
H	5.39233	-0.35098	0.51470
H	2.53113	-4.28604	0.62930
N	0.38180	1.24554	0.75847
N	0.90841	-1.36767	0.65897
C	-1.34984	2.96679	0.76557
H	-1.80744	2.67398	1.71079
H	-1.45160	4.04525	0.66301
H	-1.91630	2.49357	-0.03682
C	-0.05482	-3.60721	0.54299
H	0.20105	-4.60253	0.90312
H	-0.88880	-3.22966	1.13076
H	-0.39939	-3.71040	-0.48737
C	3.42935	4.24718	0.28966
C	3.39742	5.39748	1.09281
C	4.39131	4.19413	-0.72081
C	4.29849	6.42866	0.91096
C	5.30052	5.22638	-0.92246
C	5.26173	6.35127	-0.09941
C	4.87741	-2.98996	0.58591

C	5.89118	-2.63486	1.48821
C	5.16117	-3.99402	-0.34165
C	7.13046	-3.24478	1.45197
C	6.40542	-4.61106	-0.39765
C	7.40155	-4.23519	0.50337
H	7.90314	-2.97441	2.16017
H	4.27621	7.30526	1.54565
H	6.02008	5.14622	-1.72366
O	8.64811	-4.77320	0.53962
H	6.58483	-5.37303	-1.14154
H	5.69540	-1.88930	2.24750
H	4.42205	3.34194	-1.38665
O	6.10417	7.41143	-0.20579
C	7.10823	7.38087	-1.21701
C	8.97292	-5.79647	-0.39807
H	10.00173	-6.07736	-0.18794
H	8.89718	-5.42874	-1.42449
H	8.32370	-6.66659	-0.27219
H	7.66257	8.31025	-1.11463
H	6.66244	7.32962	-2.21359
H	7.78469	6.53432	-1.07478
H	4.39977	-4.29111	-1.05167
H	2.66422	5.47238	1.88602

7 UV/vis Spectroscopy

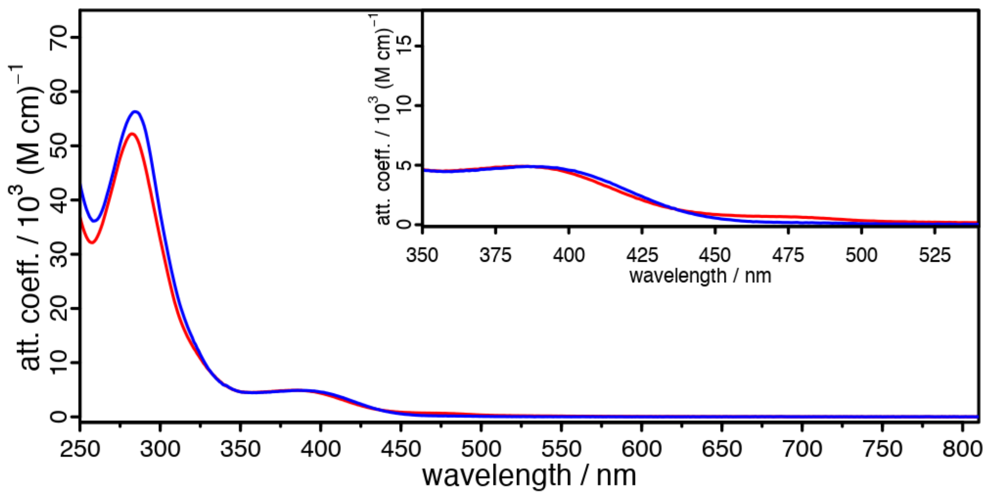

Figure S29. UV/vis absorption spectra at room temperature of **C1-o** (red) and **C2-m** (blue) in acetonitrile solution.

8 Steady-state Emission

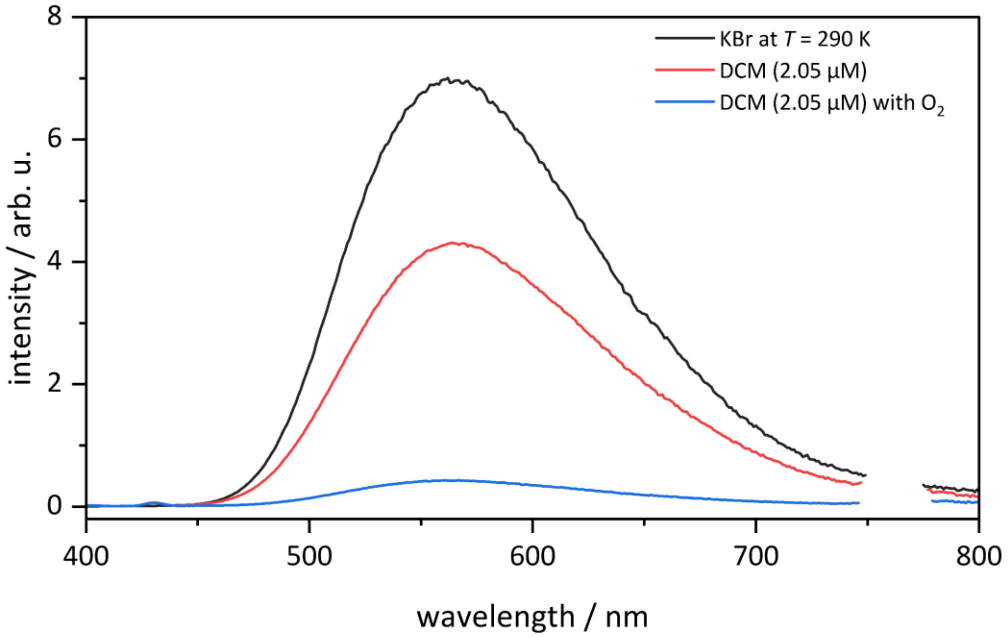

Figure S30. Photoluminescence spectra of **C1-o** prepared as KBr pellet (black line) and in dichloromethane (DCM) with (blue line) and without (red line) the presence of oxygen. For all spectra, an excitation wavelength of 380 nm was used. Missing parts of the spectra are due to masking of the excitation wavelength overtone.

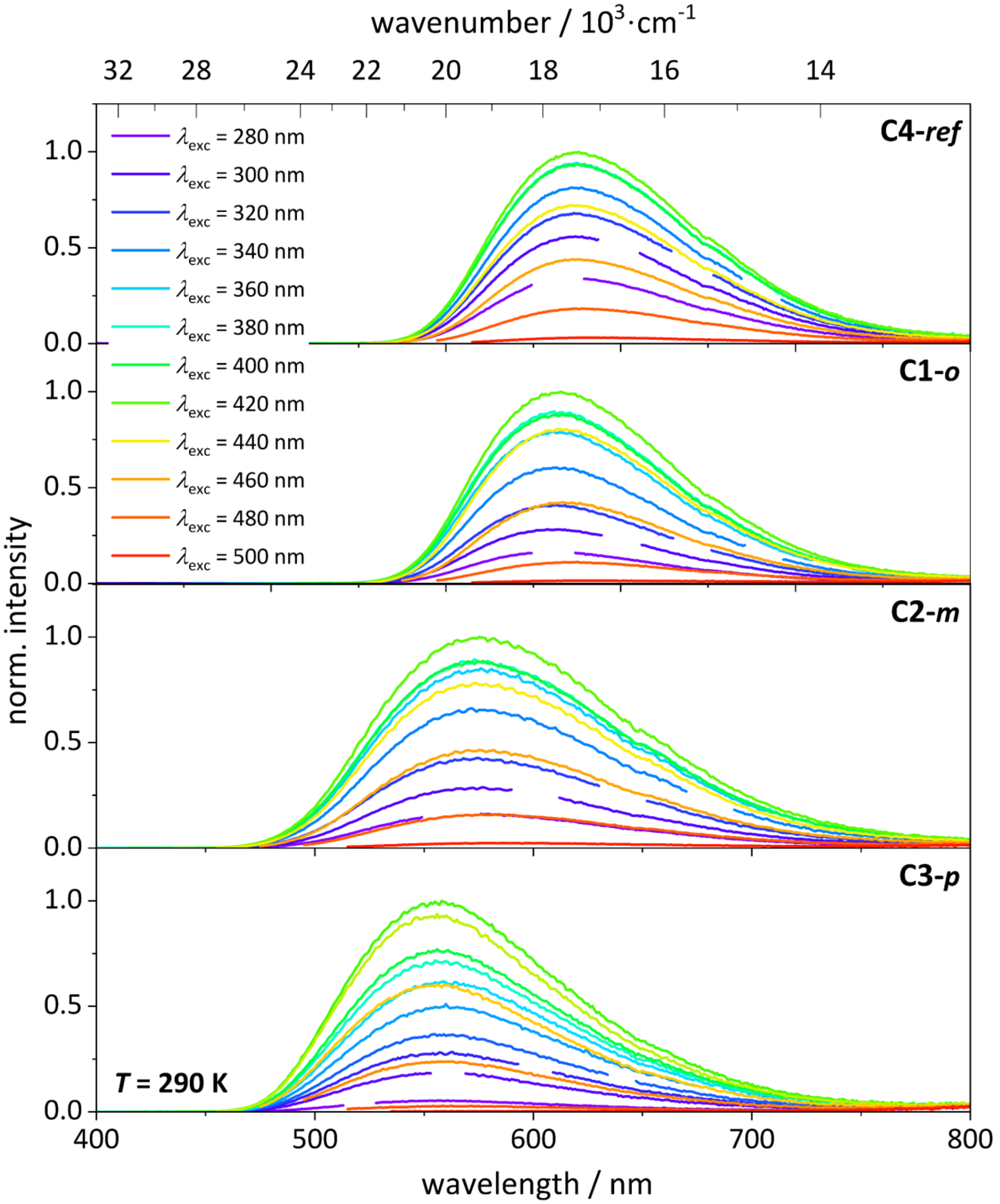

Figure S31. Photoluminescence spectra of **C4-ref** (top), **C1-o** (second from top), **C2-m** (third from top) and **C3-p** (bottom) with varying excitation wavelengths at 290 K measured in KBr matrix. Missing parts of the spectra are due to masking of the excitation wavelength overtone.

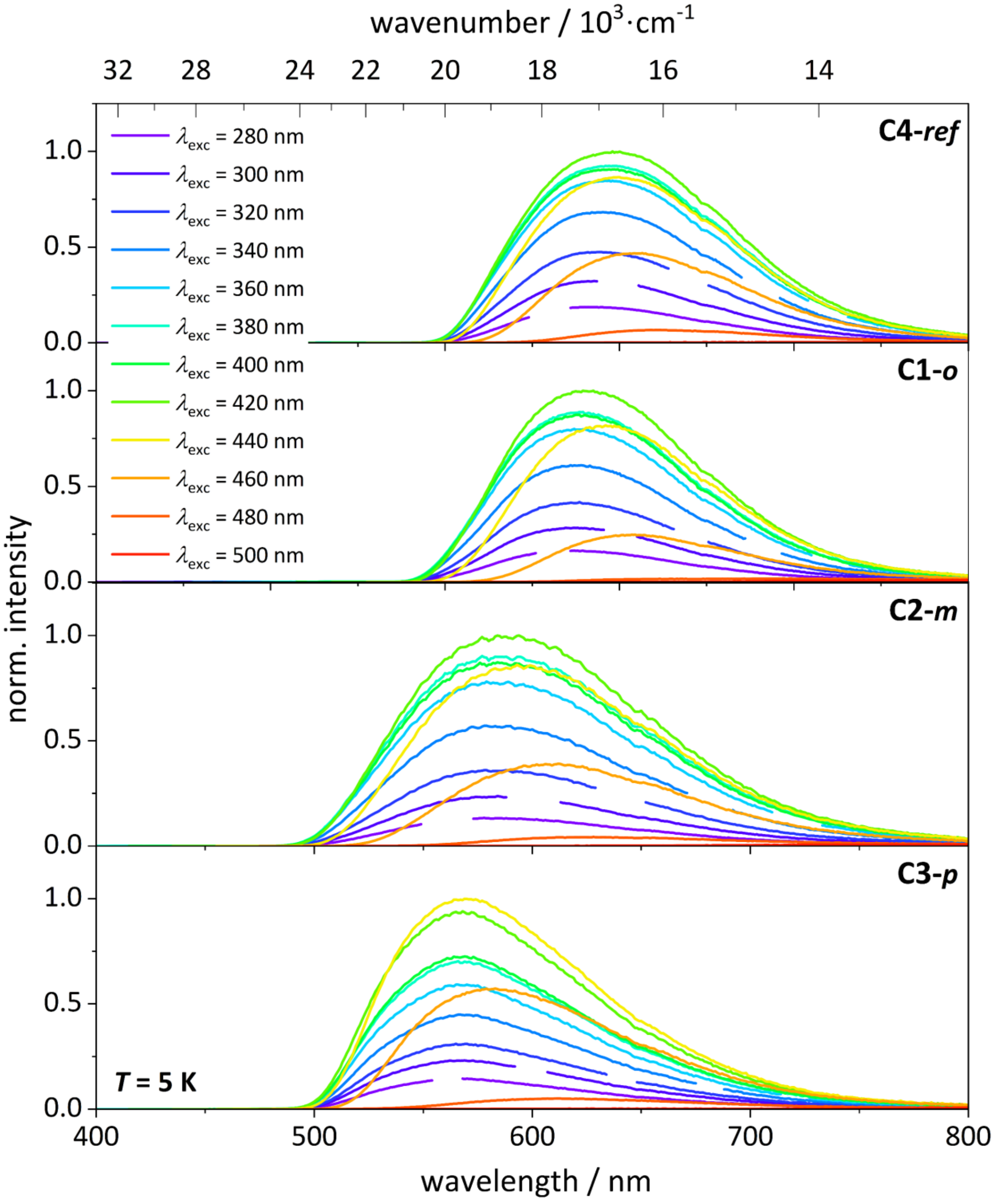

Figure S32. Photoluminescence spectra of **C4-ref** (top), **C1-o** (second from top), **C2-m** (third from top) and **C3-p** (bottom) with varying excitation wavelengths at 5 K measured in KBr matrix. Missing parts of the spectra are due to masking of the excitation wavelength overtone.

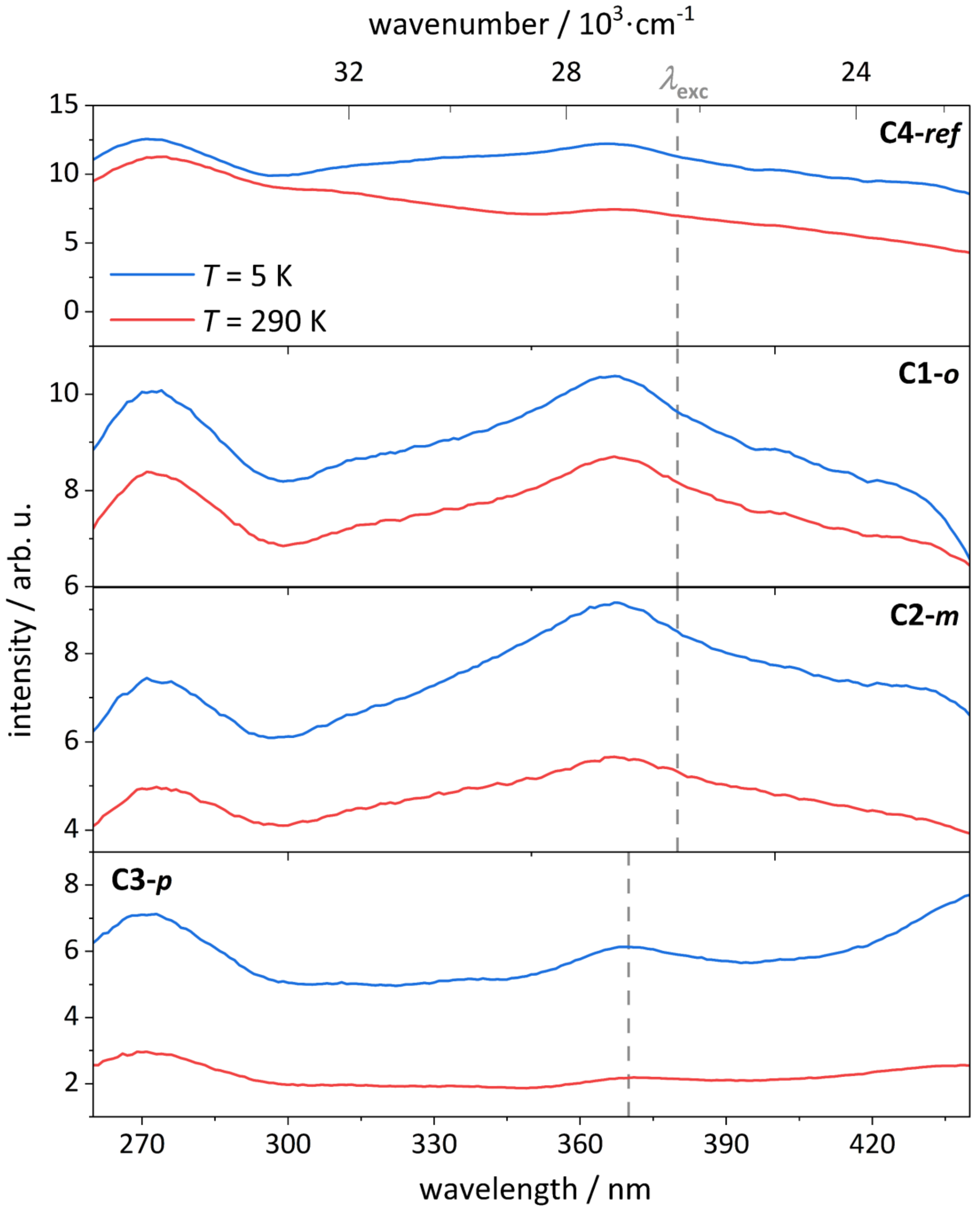

Figure S33. Excitation spectra of **C4-ref** (top), **C1-o** (second from top), **C2-m** (third from top) and **C3-p** (bottom) at 5 K (blue line) and 290 K (red line) measured in KBr matrix. The excitation wavelength used for the temperature-dependent photoluminescence spectra in Figure 3 is marked with a dashed grey line.

9 Time-resolved Emission

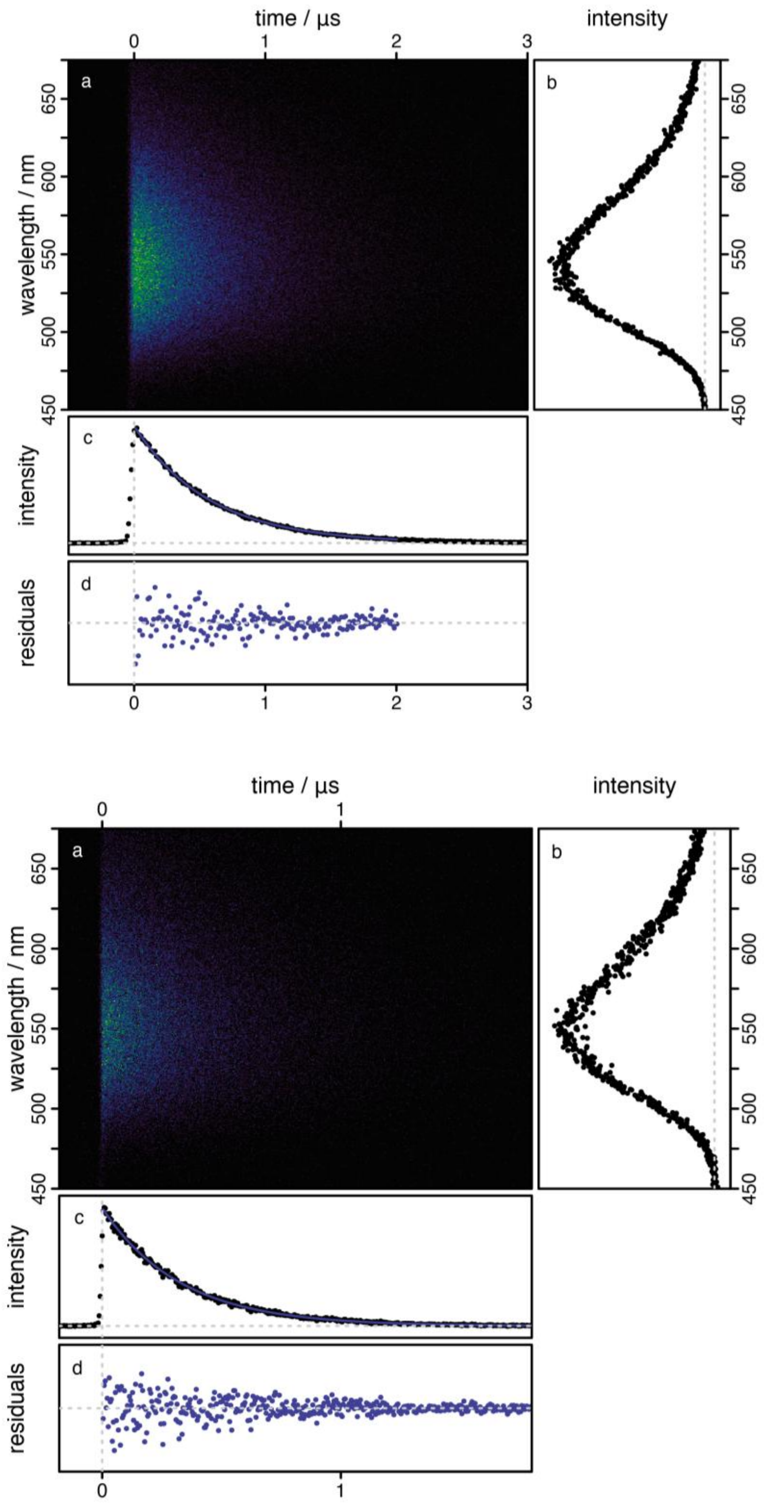

Figure S34. Streak-camera measurements of **C1-o** (top) and **C2-m** (bottom) in acetonitrile under inert conditions excited at 390 nm. The plots show the Streak images (a), the emission spectra (b), the kinetic traces (c) with the corresponding lifetime and the residuals (d) from the decay fits. The lifetimes are 576 ± 4 ns for **C1-o** and 326 ± 2 ns for **C2-m**.

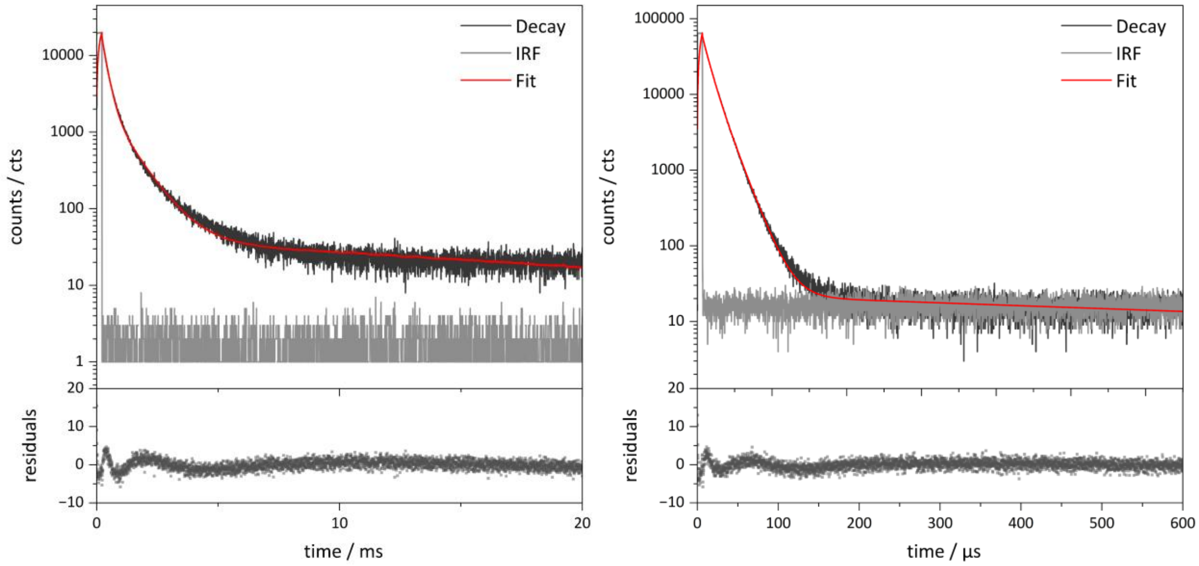

Figure S35. Excited state decay curve measured with TCSPC of **C4-ref** (black solid line) at 5 K (left) and 330 K (right) with λ_{exc} = 390 nm and fit (red solid line) of the decay using three exponentials via reconvolution fitting with the internal response function (IRF, grey solid line).

Table S11. Measurement and fit parameters of the excited state decay curves of **C4-ref** at temperatures ranging from 5 K up to 330 K with λ_{exc} = 390 nm.

T / K	λ_{em} / nm	τ_1 (A_1%)	τ_2 (A_2%)	τ_3 (A_3%)	τ_{av}
5	566	205 µs (89.9 %)	893 µs (9.9 %)	20.1 ms (0.1 %)	303 µs
10	566	203 µs (90.0 %)	886 µs (9.9 %)	21.8 ms (0.2 %)	303 µs
50	569	199 µs (89.7 %)	798 µs (10.2 %)	28.9 ms (0.1 %)	300 µs
90	572	185 µs (88.3 %)	657 µs (11.6 %)	39.8 ms (0.1 %)	290 µs
130	575	144 µs (80.0 %)	431 µs (19.7 %)	18.1 ms (0.3 %)	248 µs
170	573	91.4 µs (68.5 %)	258 µs (31.0 %)	8.34 ms (0.5 %)	181 µs
210	566	49.9 µs (62.4 %)	128 µs (37.2 %)	4.10 ms (0.5 %)	97.2 µs
250	559	26.4 µs (63.1 %)	58.4 µs (36.5 %)	4.09 ms (0.4 %)	43.8 µs
290	555	8.32 µs (32.2 %)	21.6 µs (68.0 %)	456 µs (0.8 %)	20.9 µs
330	555	8.47 µs (59.5 %)	17.0 µs (40.5 %)	1.24 ms (0.03 %)	12.3 µs

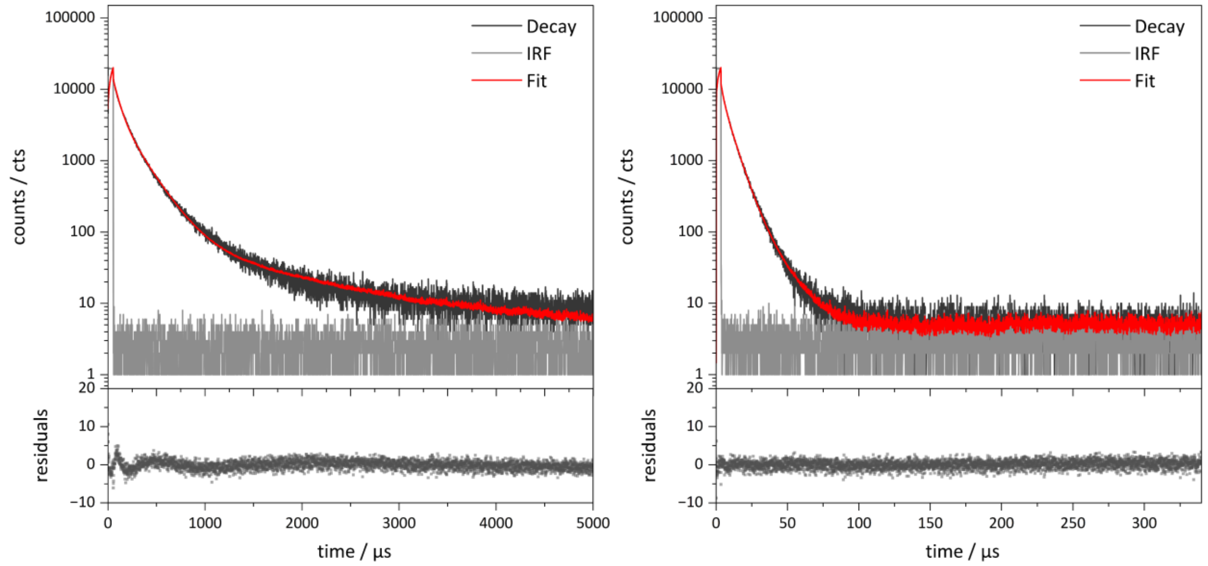

Figure S36. Excited state decay curve measured with TCSPC of **C1-o** (black solid line) at 5 K (left) and 330 K (right) with λ_{exc} = 390 nm and fit (red solid line) of the decay using three exponentials via reconvolution fitting with the internal response function (IRF, grey solid line).

Table S12. Measurement and fit parameters of the excited state decay curves of **C1-o** at temperatures ranging from 5 K up to 330 K with λ_{exc} = 390 nm.

T / K	λ_{em} / nm	τ_1 (A_1%)	τ_2 (A_2%)	τ_3 (A_3%)	τ_{av}
5	554	58.6 µs (70.8 %)	199 µs (28.6 %)	1.12 ms (0.5 %)	105 µs
10	554	61.6 µs (73.1 %)	209 µs (26.5 %)	1.38 ms (0.4 %)	106 µs
50	559	41.7 µs (55.6 %)	145 µs (41.5 %)	456 µs (2.8 %)	96.4 µs
90	562	35.8 µs (52.7 %)	132 µs (43.6 %)	378 µs (3.7 %)	90.3 µs
130	565	34.6 µs (54.2 %)	127 µs (42.7 %)	360 µs (3.1 %)	84.1 µs
170	563	22.8 µs (50.2 %)	87.2 µs (44.1 %)	217 µs (5.6 %)	62.1 µs
210	554	15.0 µs (55.5 %)	56.8 µs (42.8 %)	180 µs (1.7 %)	35.7 µs
250	548	9.50 µs (63.1 %)	31.7 µs (36.7 %)	584 µs (0.2 %)	18.5 µs
290	545	4.18 µs (57.9 %)	14.5 µs (41.9 %)	115 µs (0.2 %)	8.70 µs
330	545	1.75 µs (45.6 %)	6.35 µs (51.0 %)	14.7 µs (3.4 %)	4.54 µs

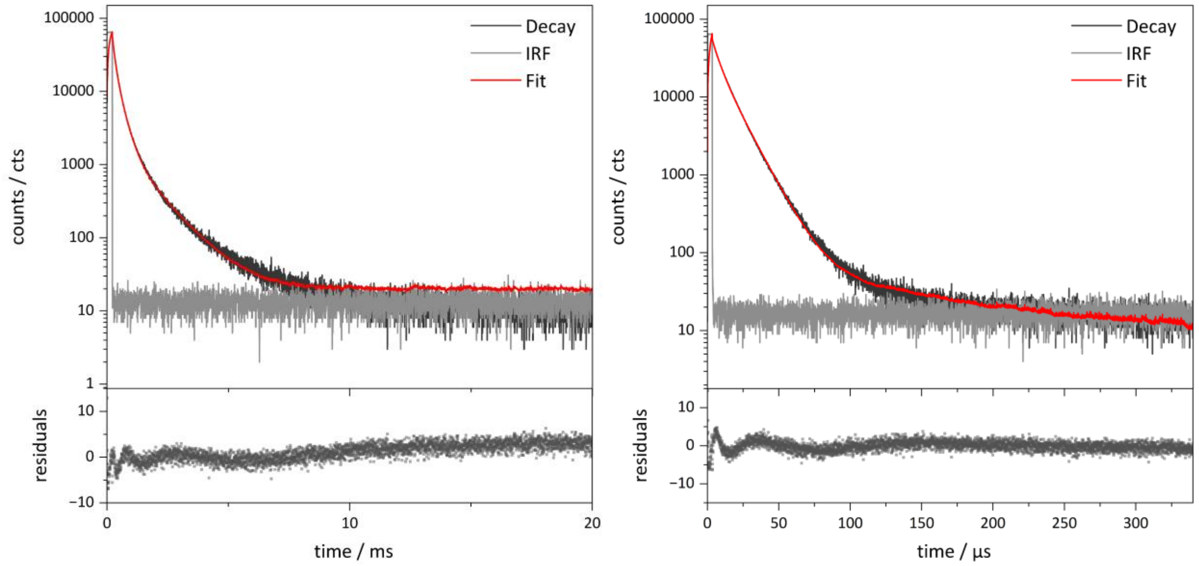

Figure S37. Excited state decay curve measured with TCSPC of **C2-m** (black solid line) at 5 K (left) and 330 K (right) with λ_{exc} = 390 nm and fit (red solid line) of the decay using three exponentials via reconvolution fitting with the internal response function (IRF, grey solid line).

Table S13. Measurement and fit parameters of the excited state decay curves of **C2-m** at temperatures ranging from 5 K up to 330 K with λ_{exc} = 390 nm.

T / K	λ_{em} / nm	τ_1 (A_1%)	τ_2 (A_2%)	τ_3 (A_3%)	τ_{av}
5	563	92.8 µs (67.4 %)	278 µs (30.6 %)	1.14 ms (2.0 %)	170 µs
10	562	97.7 µs (64.9 %)	271 µs (32.6 %)	1.05 ms (2.5 %)	178 µs
50	567	103 µs (74.0 %)	312 µs (24.9 %)	1.34 ms (1.1 %)	168 µs
90	571	112 µs (78.0 %)	329 µs (21.4 %)	1.54 ms (0.6 %)	167 µs
130	574	74.2 µs (57.4 %)	214 µs (40.8 %)	749 µs (1.8 %)	144 µs
170	571	37.2 µs (45.9 %)	137 µs (50.7 %)	421 µs (3.4 %)	101 µs
210	564	27.4 µs (45.3 %)	80.8 µs (51.8 %)	234 µs (2.9 %)	61.0 µs
250	557	9.93 µs (40.8 %)	35.8 µs (55.6 %)	91.9 µs (3.6 %)	27.3 µs
290	555	1.21 µs (59.3 %)	14.7 µs (33.5 %)	31.6 µs (7.1 %)	7.89 µs
330	555	4.51 µs (50.1 %)	12.4 µs (49.8 %)	113 µs (0.1 %)	8.56 µs

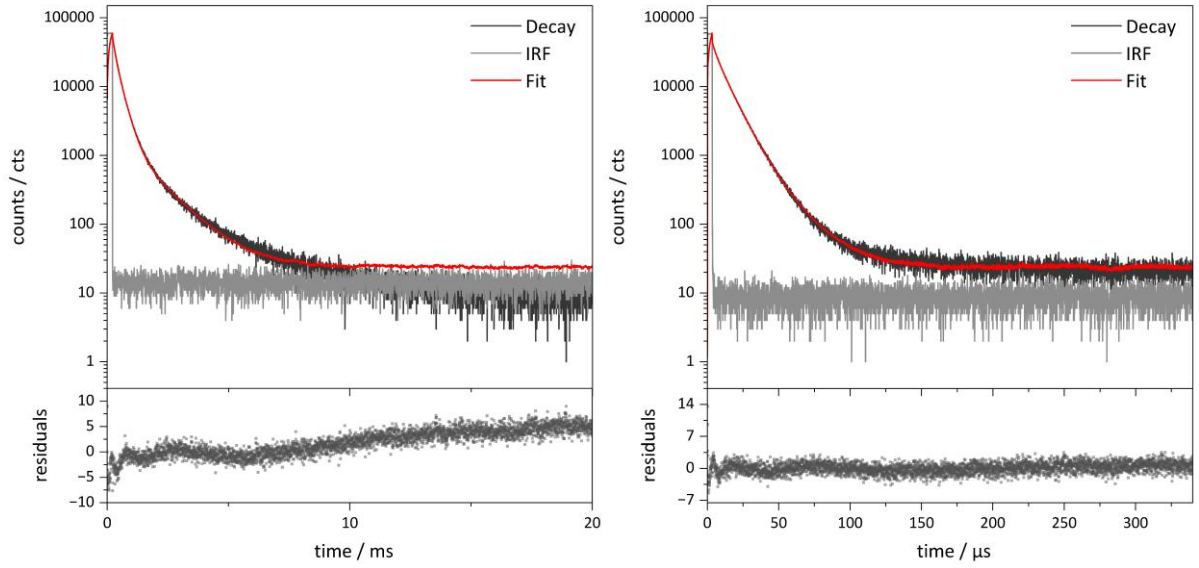

Figure S38. Excited state decay curve measured with TCSPC of **C3-p** (black solid line) at 5 K (left) and 330 K (right) with λ_{exc} = 390 nm and fit (red solid line) of the decay using three exponentials via reconvolution fitting with the internal response function (IRF, grey solid line).

Table S14. Measurement and fit parameters of the excited state decay curves of **C3-p** at temperatures ranging from 5 K up to 330 K with λ_{exc} = 390 nm.

T / K	λ_{em} / nm	τ_1 (A_1%)	τ_2 (A_2%)	τ_3 (A_3%)	τ_{av}
5	568 nm	91.3 µs (45.3 %)	281 µs (52.5 %)	1.21 ms (2.2 %)	216 µs
10	569 nm	92.5 µs (45.8 %)	280 µs (52.0 %)	1.21 ms (2.2 %)	214 µs
50	570 nm	95.4 µs (48.4 %)	278 µs (50.0 %)	1.22 ms (1.7 %)	206 µs
90	575 nm	74.7 µs (37.7 %)	245 µs (60.2 %)	932 µs (2.1 %)	195 µs
130	577 nm	55.0 µs (29.7 %)	204 µs (65.4 %)	531 µs (4.9 %)	176 µs
170	574 nm	30.7 µs (27.1 %)	133 µs (62.9 %)	280 µs (9.9 %)	120 µs
210	566 nm	12.4 µs (25.2 %)	65.3 µs (62.6 %)	130 µs (12.2 %)	59.8 µs
250	561 nm	4.37 µs (23.1 %)	29.2 µs (59.3 %)	55.6 µs (17.6 %)	28.1 µs
290	556 nm	1.76 µs (32.7 %)	14.2 µs (53.3 %)	27.7 µs (14.0 %)	12.0 µs
330	556 nm	2.12 µs (31.0 %)	9.22 µs (63.9 %)	19.9 µs (5.0 %)	7.56 µs

Table S15. Fit parameters of ΔE_{ST} from the temperature-dependent lifetimes of **C4-ref**, **C1-o**, **C2-m,** and **C3-p** using the TADF process as a model.

Complex	ΔE_{ST} / J	τ_{T1} / µs	τ_{S1} / µs
**C4-ref**	$1.436 \cdot 10^{-20} \pm 1.32 \cdot 10^{-21}$	296.4 ± 5.2	0.3029 ± 0.1513
**C1-o**	$1.395 \cdot 10^{-20} \pm 2.02 \cdot 10^{-21}$	98.63 ± 2.76	0.1341 ± 0.0994
**C2-m**	$1.420 \cdot 10^{-20} \pm 1.50 \cdot 10^{-21}$	169.3 ± 3.4	0.1895 ± 0.1062
**C3-p**	$1.464 \cdot 10^{-20} \pm 1.46 \cdot 10^{-21}$	206.4 ± 3.8	0.1675 ± 0.0937

10 Step-scan FTIR Spectroscopy

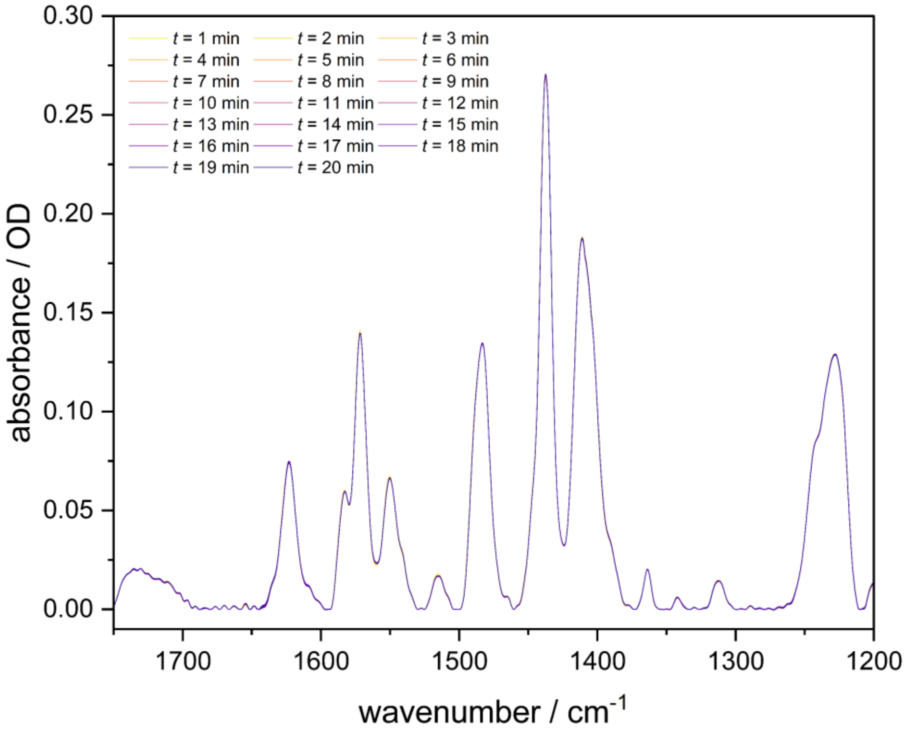

Figure S39. Steady state FTIR absorption spectra of **C4-ref** recorded every 60 seconds up to 20 min under irradiation at 355 nm with 1.4 mJ per pulse at room temperature in KBr matrix. The sample was found to be photostable, as no changes in the spectra were visible.

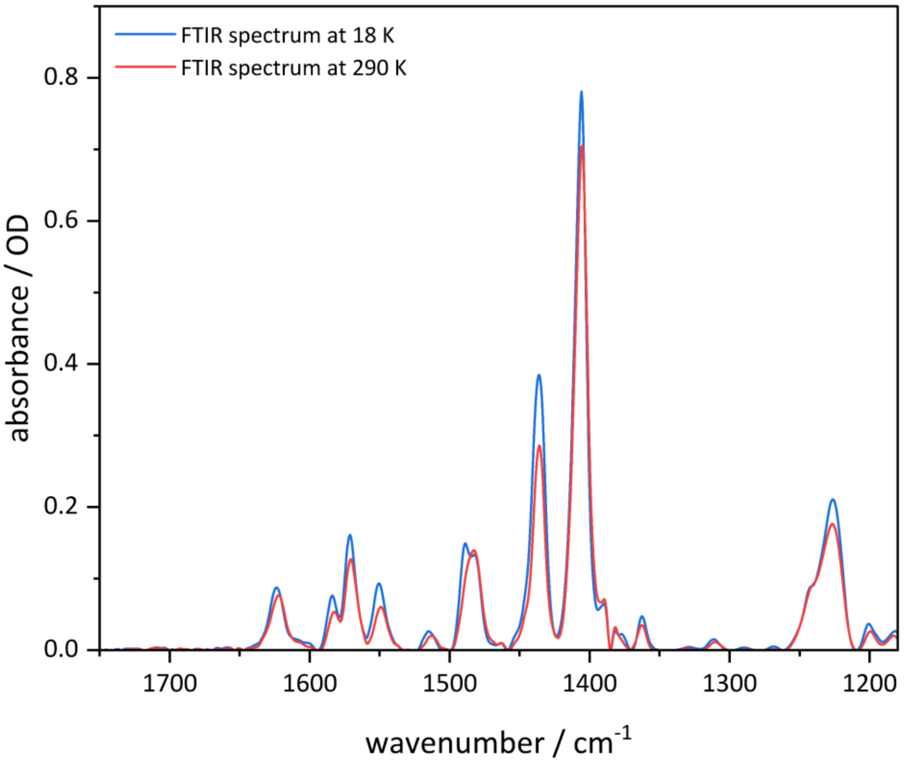

Figure S40. Steady state FTIR absorption spectra of **C4-ref** at 18 K and 290 K in KBr matrix.

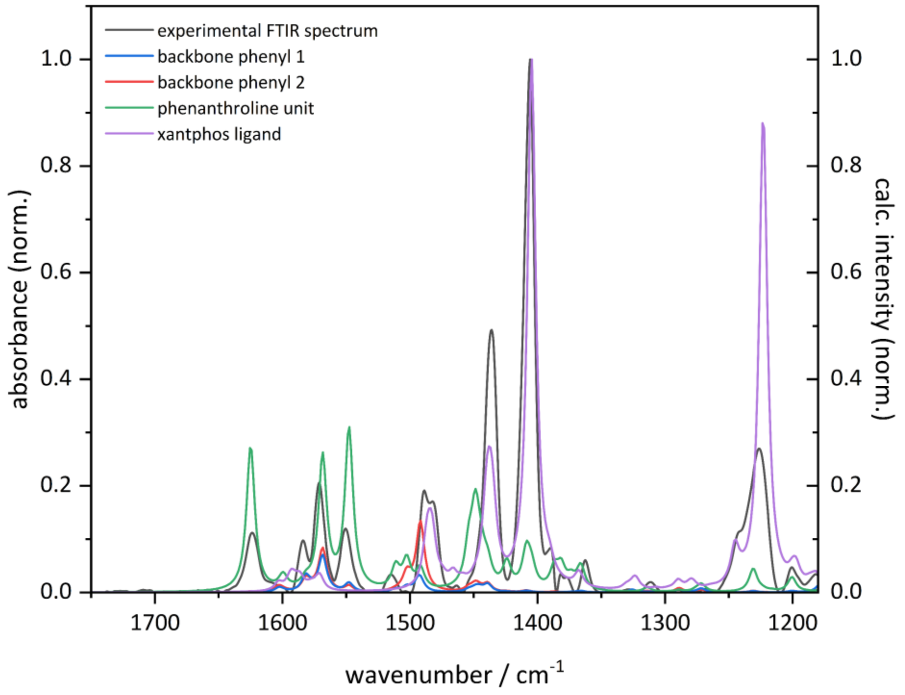

Figure S41. Experimental FTIR spectrum of **C4-ref** (black line, left axis) of the ground state at 28 K and calculated S_0 spectrum as a partial vibrational spectrum composed by the percentage contribution to the respective normal coordinate.

Table S16. Characterization of the experimental and calculated (indicated in brackets) vibrational modes of the ground state FTIR spectrum of **C4-ref** by analysis of the percentage contribution of each complex fragment to the respective normal coordinate.

$\tilde{\nu}$ / cm^{-1} ground state	Backbone phenyl 1 / %	Backbone phenyl 2 / %	Phenanthroline / %	Xantphos ligand / %
1624 (1625)	0.55	0.37	99.07	0.01
1583 (1582)	34.58	43.70	21.57	0.16
1571 (1568)	20.37	16.93	62.60	0.09
1550 (1548)	3.10	5.02	91.81	0.07
1514 (1511)	3.50	10.35	86.14	0.01
1489 (1492)	71.36	7.87	20.43	0.34
1482 (1482)	0.00	0.00	0.03	99.97
1436 (1437)	0.16	0.12	2.83	96.89
1406 (1405)	0.00	0.03	3.38	96.59
1362 (1366)	5.23	6.00	88.29	0.49
1243 (1245)	0.00	0.00	0.03	99.97
1226 (1223)	0.00	0.00	0.28	99.72

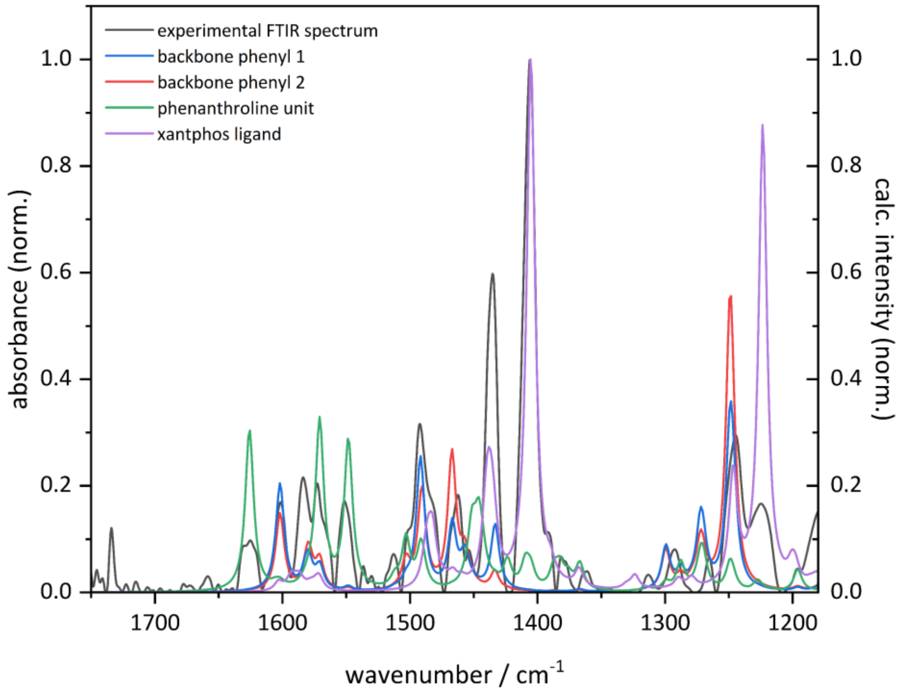

Figure S42. Experimental FTIR spectrum of **C1-o** (black line, left axis) of the ground state at 23 K and calculated S_0 spectrum as a partial vibrational spectrum composed by the percentage contribution to the respective normal coordinate.

Table S17. Characterization of the experimental and calculated (indicated in brackets) vibrational modes of the ground state FTIR spectrum of **C1-o** by analysis of the percentage contribution of each complex fragment to the respective normal coordinate.

$\tilde{\nu}$ / cm^{-1} ground state		Backbone phenyl 1 / %	Backbone phenyl 2 / %	Phenanthroline / %	Xantphos ligand / %
1627	(1627)	0.32	0.18	99.50	0.00
1602	(1602)	40.36	56.86	2.56	0.22
1583	(1580)	54.45	38.10	6.90	0.55
1572	(1570)	13.28	10.44	76.05	0.23
1551	(1548)	1.63	2.73	95.58	0.06
1501	(1503)	32.20	9.68	58.06	0.06
1492	(1492)	0.13	84.23	15.28	0.36
	(1491)	79.21	1.70	18.65	0.45
1482	(1482)	0.00	0.00	0.03	99.97
1462	(1467)	82.83	16.24	0.64	0.29
1435	(1433)	9.87	78.22	5.90	6.02
1406	(1405)	0.01	0.04	3.33	96.62
/	(1272)	32.56	46.11	21.13	0.21
/	(1270)	22.78	40.27	36.86	0.09
1245	(1249)	60.58	32.69	5.94	0.79
1223	(1224)	0.05	0.05	0.28	99.62

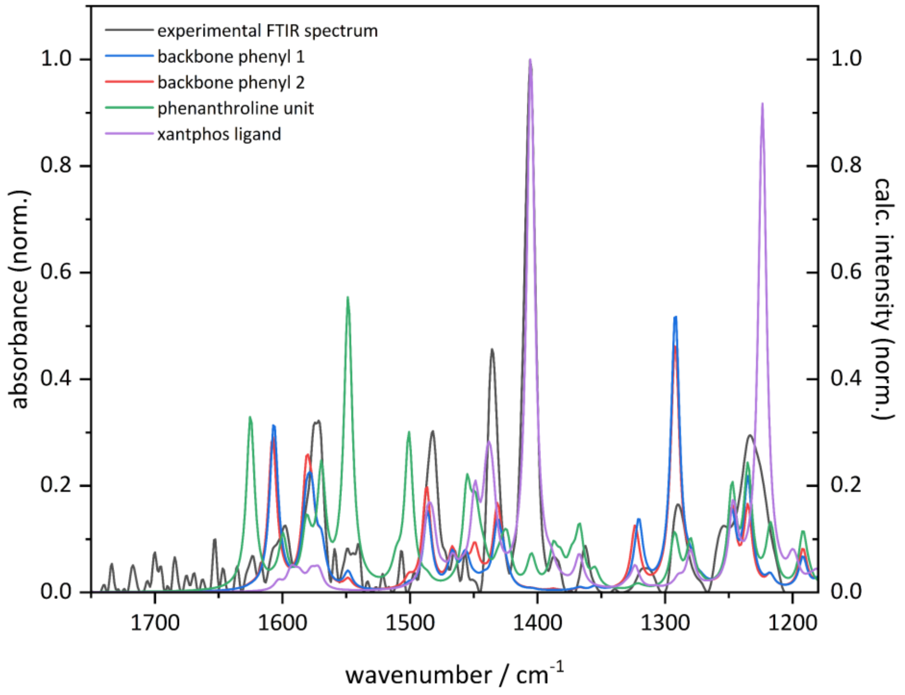

Figure S43. Experimental FTIR spectrum of **C2-m** (black line, left axis) of the ground state at 21 K and calculated S_0 spectrum as a partial vibrational spectrum composed by the percentage contribution to the respective normal coordinate.

Table S18. Characterization of the experimental and calculated (indicated in brackets) vibrational modes of the ground state FTIR spectrum of **C2-m** by analysis of the percentage contribution of each complex fragment to the respective normal coordinate.

$\tilde{\nu}$ / cm^{-1} ground state		Backbone phenyl 1 / %	Backbone phenyl 2 / %	Phenanthroline / %	Xantphos ligand / %
/	(1625)	0.61	0.40	98.99	0.00
/	(1608)	98.27	0.68	1.03	0.02
/	(1607)	0.75	98.43	0.81	0.01
1598	(1600)	3.76	2.02	93.91	0.31
/	(1581)	44.79	30.88	24.20	0.14
1574	(1577)	41.17	50.08	6.21	2.53
	(1569)	20.04	20.13	59.54	0.30
1545	(1549)	2.99	5.38	91.56	0.06
/	(1501)	6.11	2.53	91.16	0.20
1482	(1487)	88.15	1.20	3.03	7.62
	(1486)	0.87	95.19	3.57	0.37
/	(1455)	4.78	13.99	79.83	1.39
/	(1449)	25.35	2.87	36.06	35.72
1436	(1432)	51.31	39.26	9.23	0.20
1406	(1405)	0.02	0.02	3.48	96.48
1362	(1367)	3.74	4.42	85.30	6.55
1289	(1293)	42.52	36.86	20.54	0.09
	(1292)	42.79	50.59	6.58	0.03
1254	(1248)	30.16	27.54	38.08	4.22
1233	(1235)	26.04	35.38	38.53	0.05
/	(1224)	0.00	0.01	0.33	99.66
/	(1192)	31.38	24.89	43.58	0.14

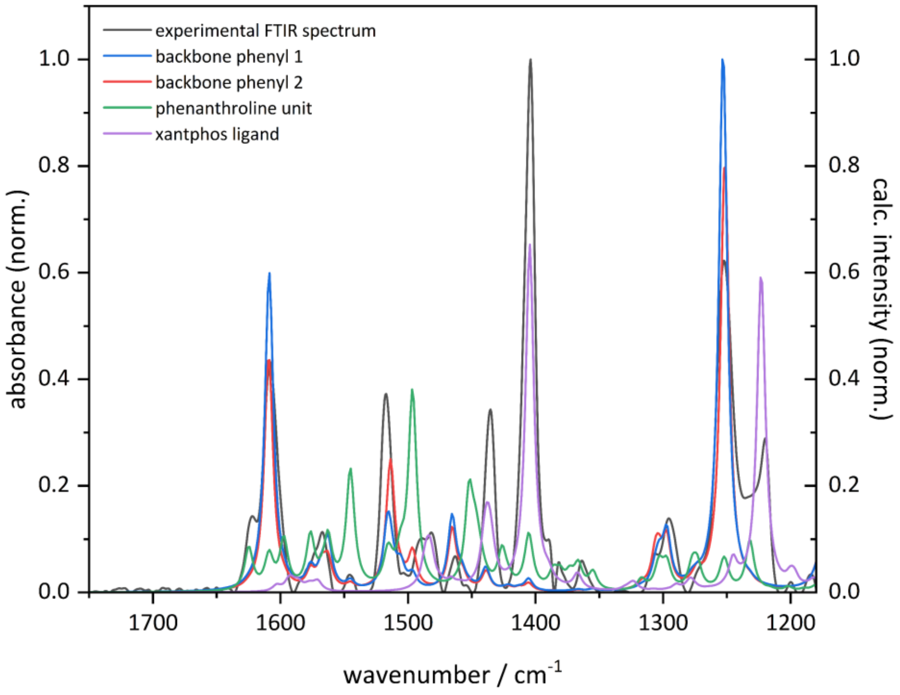

Figure S44. Experimental FTIR spectrum of **C3-p** (black line, left axis) of the ground state at 19 K and calculated S_0 spectrum as a partial vibrational spectrum composed by the percentage contribution to the respective normal coordinate.

Table S19. Characterization of the experimental and calculated (indicated in brackets) vibrational modes of the ground state FTIR spectrum of **C3-p** by analysis of the percentage contribution of each complex fragment to the respective normal coordinate.

$\tilde{\nu}$ / cm^{-1} ground state		Backbone phenyl 1 / %	Backbone phenyl 2 / %	Phenanthroline / %	Xantphos ligand / %
1622	(1625)	3.44	3.37	93.18	0.00
1608	(1609)	32.68	61.45	5.87	0.00
/	(1577)	18.81	21.75	58.31	1.13
1567	(1568)	54.44	34.04	11.47	0.06
/	(1563)	22.52	38.27	39.15	0.06
1545	(1545)	4.09	6.13	89.70	0.07
1518	(1516)	9.81.	61.02	29.17	0.00
	(1514)	78.76	15.28	5.96	0.00
/	(1496)	14.56	5.56	79.60	0.28
1486	(1487)	0.00	0.00	0.09	99.91
	(1482)	0.00	0.00	0.03	99.97
1462	(1465)	6.21	92.92	0.54	0.33
	(1465)	93.94	5.79	0.16	0.11
/	(1452)	0.45	2.69	96.04	0.83
1435	(1437)	0.09	0.06	1.76	98.12
1404	(1405)	0.07	0.02	3.36	96.55
1307	(1306)	81.30	17.56	1.14	0.00
1295	(1297)	36.96	42.63	20.32	0.09
1252	(1253)	11.05	86.24	2.64	0.07
	(1251)	84.89	10.86	4.09	0.05
1220	(1223)	0.00	0.00	0.27	99.73

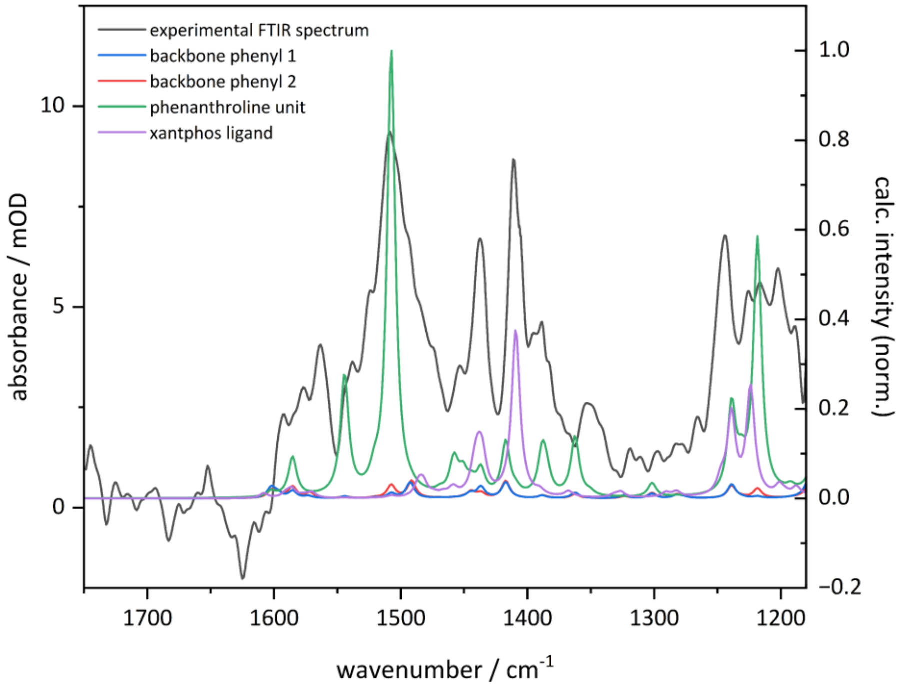

Figure S45. Experimental FTIR spectrum of **C4-ref** (black line, left axis) of the excited state at 28 K and calculated T_1 spectrum as a partial vibrational spectrum composed by the percentage contribution to the respective normal coordinate.

Table S20. Characterization of the experimental and calculated (indicated in brackets) vibrational modes of the excited state FTIR spectrum of **C4-ref** by analysis of the percentage contribution of each complex fragment to the respective normal coordinate.

$\tilde{\nu}$ / cm^{-1} excited state		Backbone phenyl 1 / %	Backbone phenyl 2 / %	Phenanthroline / %	Xantphos ligand / %
1592	(1592)	0.15	0.01	0.08	99.76
	(1585)	19.32	12.00	67.58	1.09
1577	(1578)	0.00	0.03	0.09	99.88
	(1577)	0.00	0.00	0.04	99.96
1564	/				
/	(1544)	1.47	1.58	96.76	0.20
1538	/				
1524	(1521)	4.30	4.71	91.00	0.00
1510	(1508)	2.80	1.03	95.77	0.40
1454	(1451)	3.94	1.42	93.96	0.67
1438	(1437)	9.98	21.63	47.48	20.91
1411	(1409)	0.07	0.11	2.81	97.01
1389	(1388)	5.26	5.89	88.84	0.01
/	(1362)	6.08	5.12	85.12	0.69
1351	(1351)	5.28	4.01	90.07	0.64
1266	(1265)	0.00	0.00	0.00	100.00
1245	(1239)	2.88	3.25	19.68	74.19
	(1239)	8.24	9.15	57.68	24.93
1226	(1224)	0.05	0.00	0.84	99.11
1216	(1218)	3.53	0.61	99.53	0.34
1202	(1201)	0.04	0.00	0.82	99.14

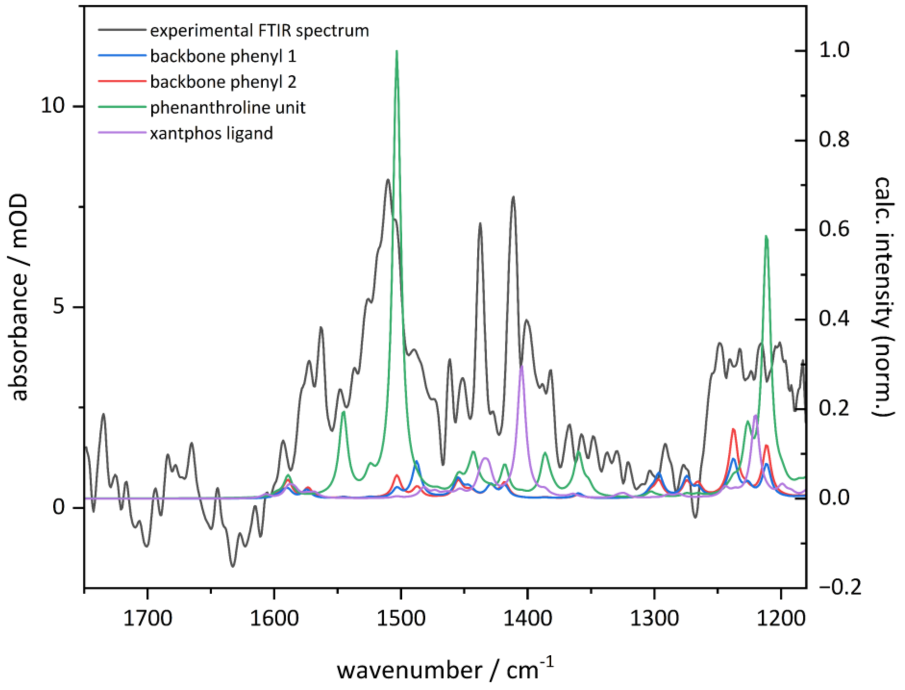

Figure S46. Experimental FTIR spectrum of **C1-o** (black line, left axis) of the excited state at 23 K and calculated T_1 spectrum as a partial vibrational spectrum composed by the percentage contribution to the respective normal coordinate.

Table S21. Characterization of the experimental and calculated (indicated in brackets) vibrational modes of the excited state FTIR spectrum of **C1-o** by analysis of the percentage contribution of each complex fragment to the respective normal coordinate.

$\tilde{\nu}$ / cm^{-1} excited state		Backbone phenyl 1 / %	Backbone phenyl 2 / %	Phenanthroline / %	Xantphos ligand / %
/	(1598)	26.0	27.2	46.8	0.02
1593	1593	38.8	58.3	2.77	0.17
/	(1589)	34.4	15.4	44.6	5.64
1572	(1573)	86.2	10.6	2.25	0.95
1563	/				
1548	(1545)	1.49	1.38	97.0	0.18
1526	(1525)	5.12	5.18	89.7	0.00
1510	(1503)	4.72	1.93	93.01	0.33
1489	(1488)	17.2	78.9	3.68	0.21
1461	(1461)	0.12	0.05	0.47	99.4
1452	(1455)	43.4	55.2	1.08	0.39
	(1453)	28.7	16.9	45.2	9.16
1437	(1437)	0.05	0.33	8.58	91.0
	(1435)	0.02	0.04	2.70	97.2
/	(1418)	26.0	20.4	53.1	0.42
1412	(1405)	0.04	0.07	1.82	98.1
1400	/				
1249	/				
/	(1238)	59.1	31.2	9.21	0.56
1216	(1220)	0.11	0.03	1.23	98.6
/	(1211)	15.0	9.57	75.0	0.47
1202	/				

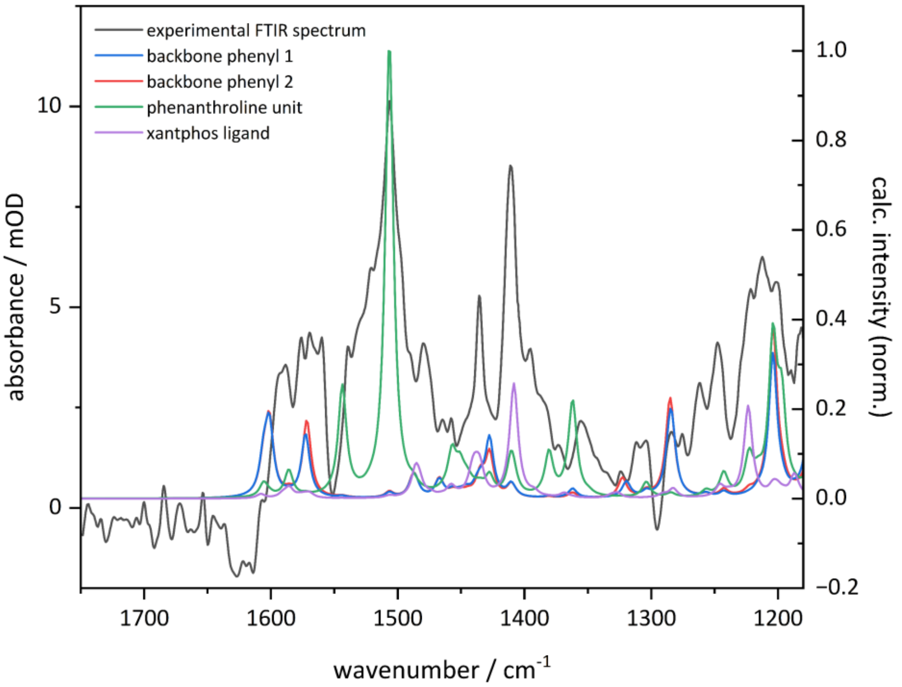

Figure S47. Experimental FTIR spectrum of **C2-m** (black line, left axis) of the excited state at 21 K and calculated T_1 spectrum as a partial vibrational spectrum composed by the percentage contribution to the respective normal coordinate.

Table S22. Characterization of the experimental and calculated (indicated in brackets) vibrational modes of the excited state FTIR spectrum of **C2-m** by analysis of the percentage contribution of each complex fragment to the respective normal coordinate.

$\tilde{\nu}$ / cm^{-1} excited state		Backbone phenyl 1 / %	Backbone phenyl 2 / %	Phenanthroline / %	Xantphos ligand / %
/	(1606)	39.4	43.4	17.1	0.01
/	(1601)	50.3	48.0	1.63	0.00
1591	(1591)	0.00	0.00	0.07	99.9
1570	(1573)	0.02	98.9	1.07	0.02
	(1572)	97.8	0.00	1.12	0.92
1539	(1544)	1.35	1.31	97.1	0.20
1521	(1522)	3.91	3.79	92.3	0.00
1506	(1507)	1.25	1.10	97.2	0.45
1480	(1484)	0.03	0.00	0.04	99.9
	(1483)	0.00	0.02	0.06	99.9
1436	(1435)	51.1	28.9	15.8	4.14
/	(1428)	35.9	48.5	15.5	0.06
1411	(1411)	18.0	17.8	56.0	8.20
	(1408)	1.75	1.56	7.78	88.9
1395	(1393)	0.00	0.00	0.04	100
/	(1362)	4.67	8.31	86.4	0.66
1355	/				
1308	(1304)	21.9	19.4	58.4	0.33
1284	(1285)	64.0	34.1	1.68	0.25
	(1284)	30.7	65.5	2.82	0.90
1262	(1264)	0.00	0.00	0.00	100
1248	(1246)	0.00	0.01	0.01	100
	(1243)	20.3	14.2	65.4	0.02
1222	(1224)	1.78	0.23	11.5	86.5
	(1223)	8.72	1.03	77.9	12.4
1213	/				
/	(1204)	35.7	30.2	32.2	1.92
/	(1197)	11.7	5.70	81.9	0.66

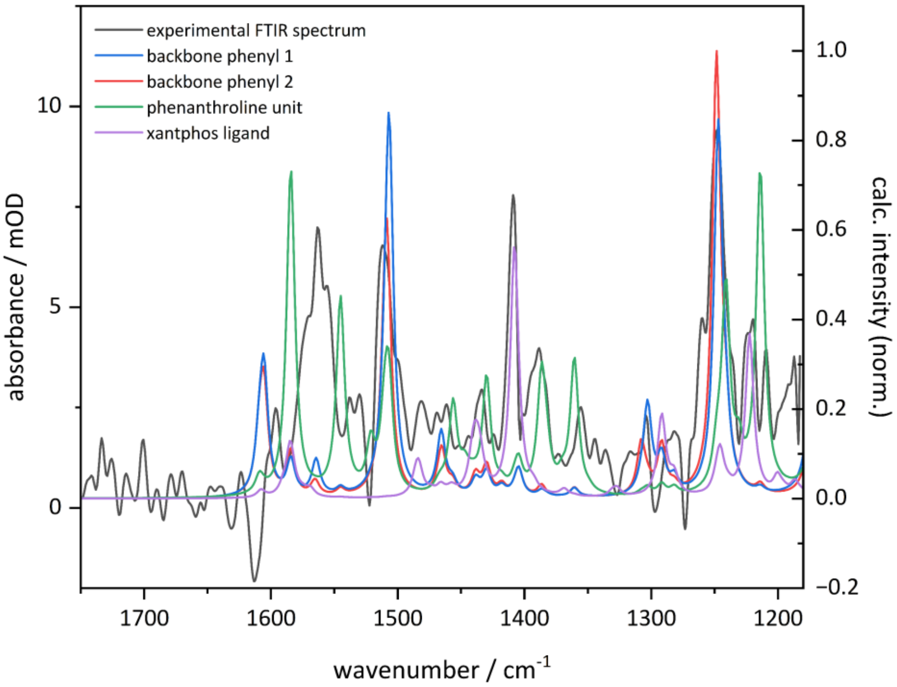

Figure S48. Experimental FTIR spectrum of **C3-p** (black line, left axis) of the excited state at 21 K and calculated T_1 spectrum as a partial vibrational spectrum composed by the percentage contribution to the respective normal coordinate.

Table S23. Characterization of the experimental and calculated (indicated in brackets) vibrational modes of the excited state FTIR spectrum of **C3-p** by analysis of the percentage contribution of each complex fragment to the respective normal coordinate.

$\tilde{\nu}$ / cm^{-1} excited state		Backbone phenyl 1 / %	Backbone phenyl 2 / %	Phenanthroline / %	Xantphos ligand / %
/	(1609)	46.3	38.8	15.0	0.00
/	(1606)	45.3	53.9	0.85	0.00
1596	/				
/	(1584)	10.2	7.97	74.2	7.59
1563	(1564)	12.2	85.7	2.00	0.13
/	(1545)	3.12	3.13	93.5	0.29
1534	/				
1512	(1509)	70.6	15.5	13.8	0.05
	(1507)	3.09	78.5	18.4	0.03
1435	(1430)	17.5	13.8	67.2	1.50
1409	(1408)	0.65	0.93	2.91	95.5
	(1405)	33.4	32.2	33.1	2.33
1388	(1387)	8.90	4.52	86.5	0.08
/	(1361)	6.13	5.81	87.4	0.65
1355	/				
1304	(1308)	84.06	11.35	4.60	0.00
	(1303)	7.04	85.5	7.45	0.03
1282	(1282)	17.2	34.1	17.1	31.6
1260	/				
	(1249)	79.7	14.2	4.53	1.51
1248	(1247)	10.3	84.9	3.97	0.84
	(1241)	17.4	13.3	68.6	0.69
1222	(1222)	0.07	0.01	0.51	99.4
/	(1214)	2.58	1.54	95.5	0.33
1209	/				

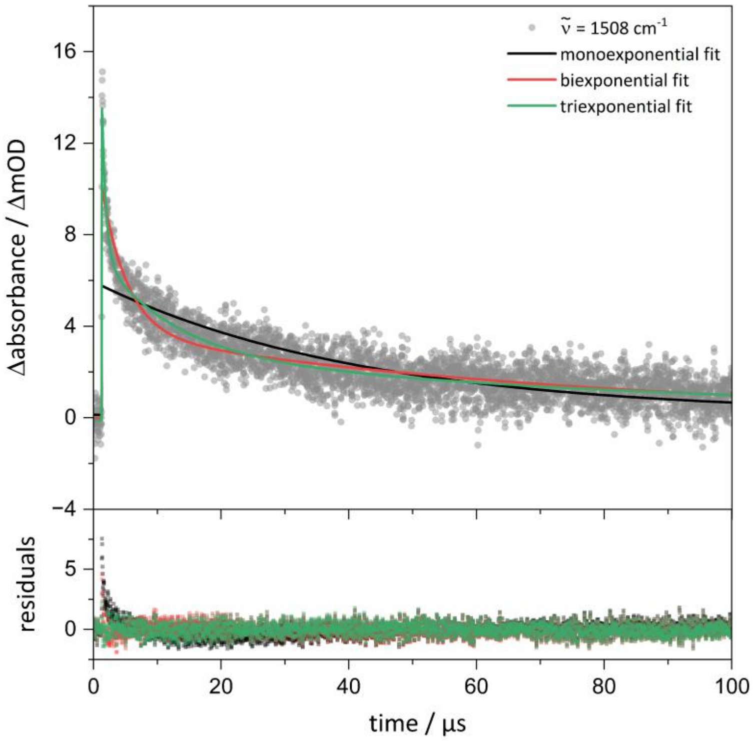

Figure S49. Decay of the excited state feature exemplary at 1508 cm^{-1} of **C4-ref** (black dots upper panel) in the step-scan FTIR spectrum at 28 K fitted mono- (black line), bi- (red line) and triexponentially (green line).

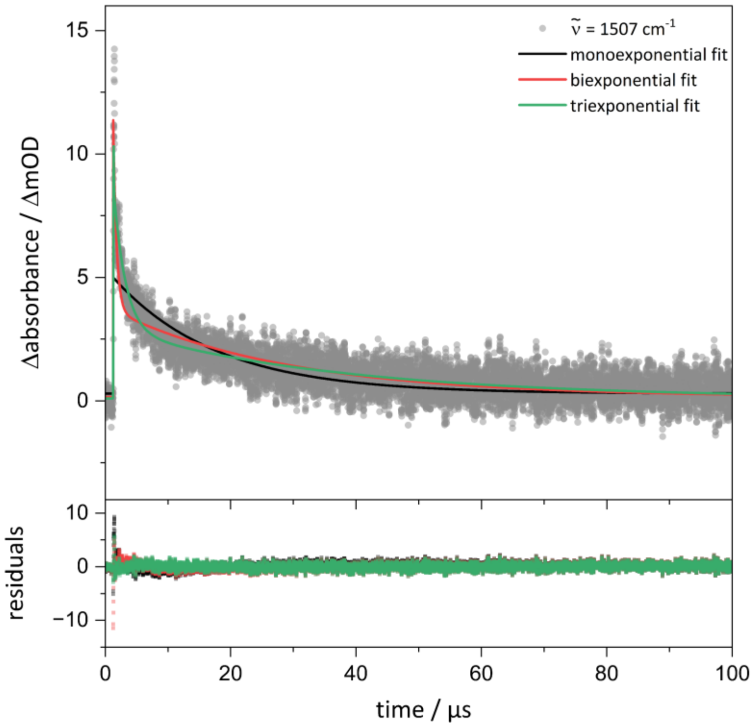

Figure S50. Decay of the excited state feature exemplary at 1507 cm^{-1} of **C1-o** (black dots upper panel) in the step-scan FTIR spectrum at 18 K fitted mono- (black line), bi- (red line) and triexponentially (green line).

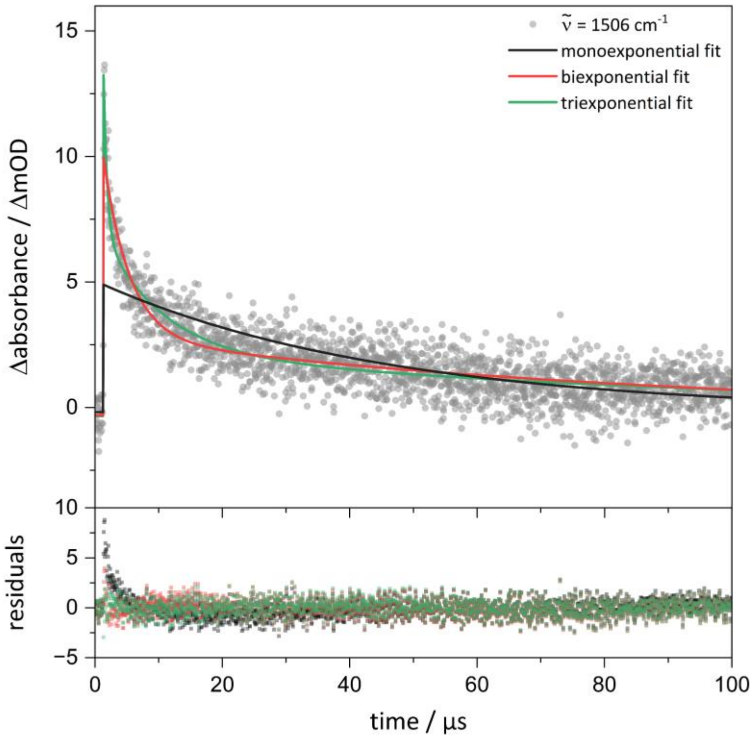

Figure S51. Decay of the excited state feature exemplary at 1506 cm^{-1} of **C2-m** (black dots upper panel) in the step-scan FTIR spectrum at 21 K fitted mono- (black line), bi- (red line) and triexponentially (green line).

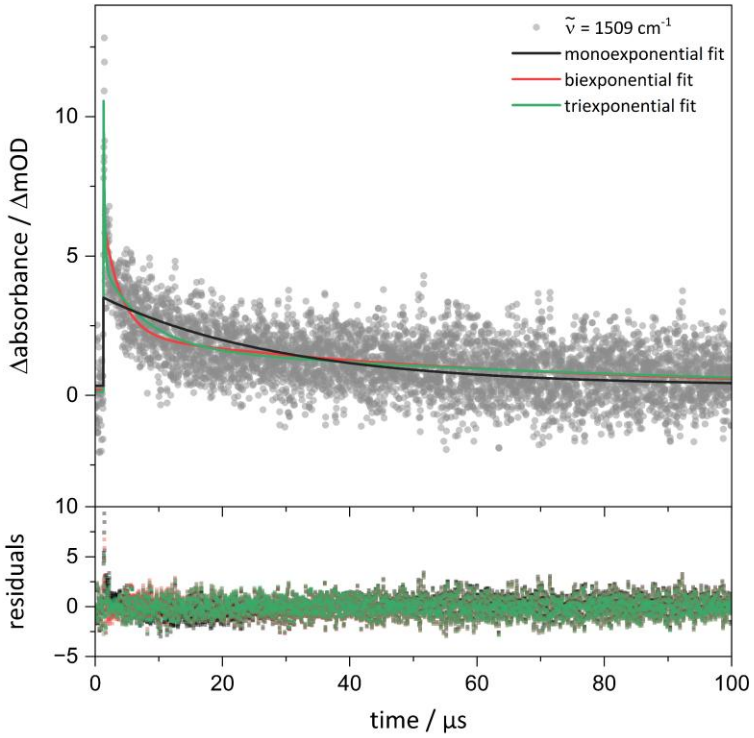

Figure S52. Decay of the excited state feature exemplary at 1509 cm^{-1} of **C3-p** (black dots upper panel) in the step-scan FTIR spectrum at 19 K fitted mono- (black line), bi- (red line) and triexponentially (green line).

Table S24. Global fit parameter[1] of the used modified Gaussian (see eq. 1.3) of the kinetic decays obtained by step-scan FTIR spectroscopy. The amplitudes *A* (%) and the offset y_0 were individual parameters for each decay, and x_c was kept fixed at the excitation time of 1.3 µs. The remaining parameters were fitted globally using the kinetic traces extracted from the excited state absorption and ground state bleach. The complex **C3-p** was fitted after transformation to the µs time scale, causing $\boldsymbol{\omega}$ to contract.

Complex	τ_1 / µs	τ_2 / µs	τ_3 / µs	τ_{av} / µs	ω
**C4-ref**	0.6 (1.4%)	11.5 (13.4%)	103.7 (85.1%)	89.8	18.23
**C1-o**	0.04 (1.1%)	1.8 (8.6%)	36.6 (90.3%)	33.2	25.52
**C2-m**	0.6 (1.3%)	9.4 (12.1%)	110.6 (86.6%)	96.6	15.20
**C3-p**	0.2 (1.4%)	6.4 (11.8%)	84.0 (86.9%)	73.7	$2.8 \cdot 10^{-3}$

[1] Deviations between the observed lifetime from TCSPC and step-scan FTIR are probably related to the strongly differing excitation conditions in both measurements. The high excitation intensities in step-scan FT-IR cause potential local heating and with this a shortening of the observed lifetimes.

11 Electrochemical Data

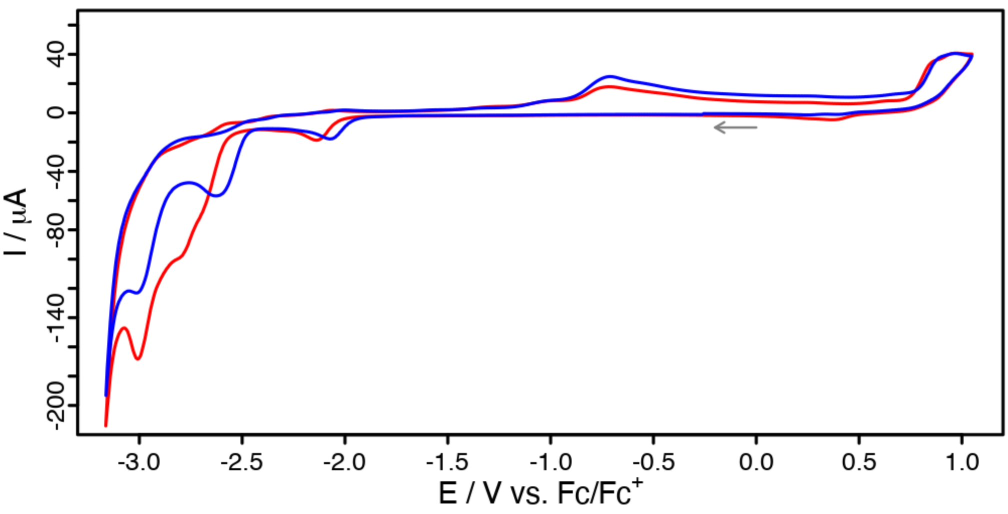

Figure S53. Cyclic voltammograms of **C1-o** (blue) and **C2-m** (red) in deaerated MeCN solution (*c* = 1 mM) with $[Bu_4N][PF_6]$ as supporting electrolyte referenced *vs*. the ferrocene/ferrocenium (Fc/Fc^+) couple. The initial scan direction is marked by the grey arrow.

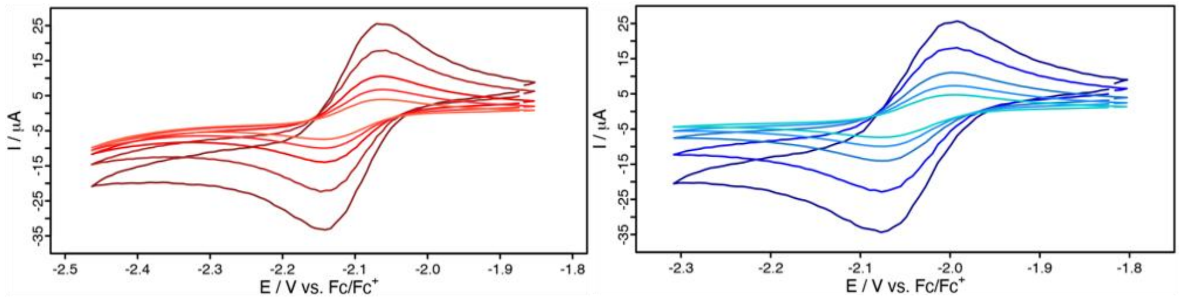

Figure S54. Excerpt from the first reversible reduction event of **C1-o** (left) and **C2-m** (right) with different scan rates: 0.5 V/s, 0.25 V/s, 0.1 V/s, 0.05 V/s, 0.025 V/s (from dark to bright).

12 Excited State Reduction Potentials

The excited state potential was determined via the simplified REHM–WELLER[19,20] equation:

$$E^{*}_{1/2}{}^{red} = E_{1/2}{}^{red} + E_{0,0} \qquad \text{eq. 1.9}$$

For determination of $E_{0,0}$ the intercept wavelength was estimated via the emission spectrum (Fig. S34) and converted into eV.

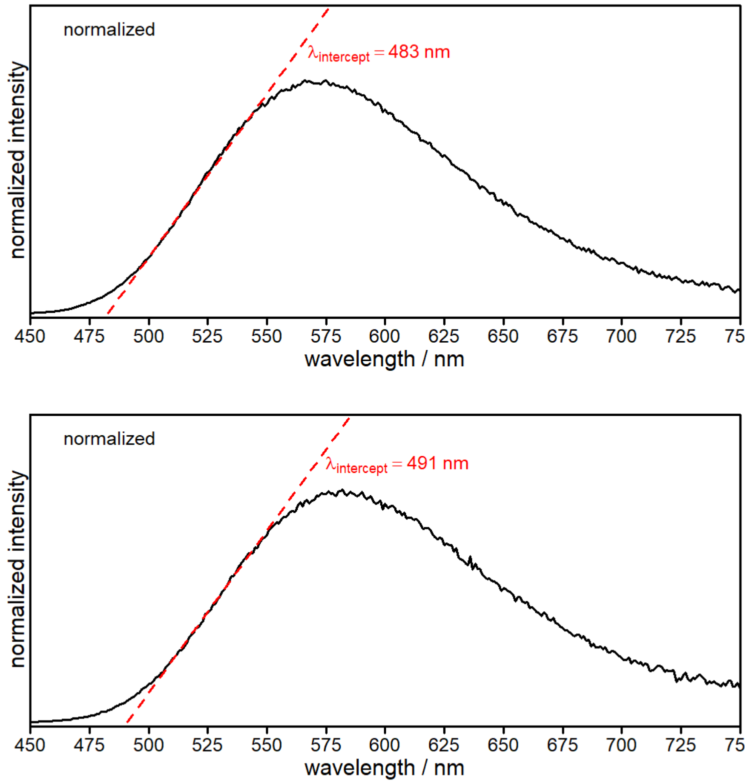

Figure S55. Determination of the intercept wavelength of **C1-o** (top) and **C2-m** (bottom).

Table S25. Determined and calculated values for $E^{*}_{1/2}{}^{red}$.

**Complex**	$E_{1/2}$red [V]	$E_{0,0}$ [nm]	$E_{0,0}$ [eV]	$E^{*}_{1/2}{}^{red}$ [V *vs*. Fc/Fc^{+}]
**C1-o**	-2.11	483	2.57	0.46
**C2-m**	-2.03	491	2.53	0.50
**C3-p**	-2.08	484	2.56	0.48
**C4-ref**	-2.04	489	2.53	0.49

[a)] Values for **C4-ref** and **C3-p** from literature.[21]

13 Photostability

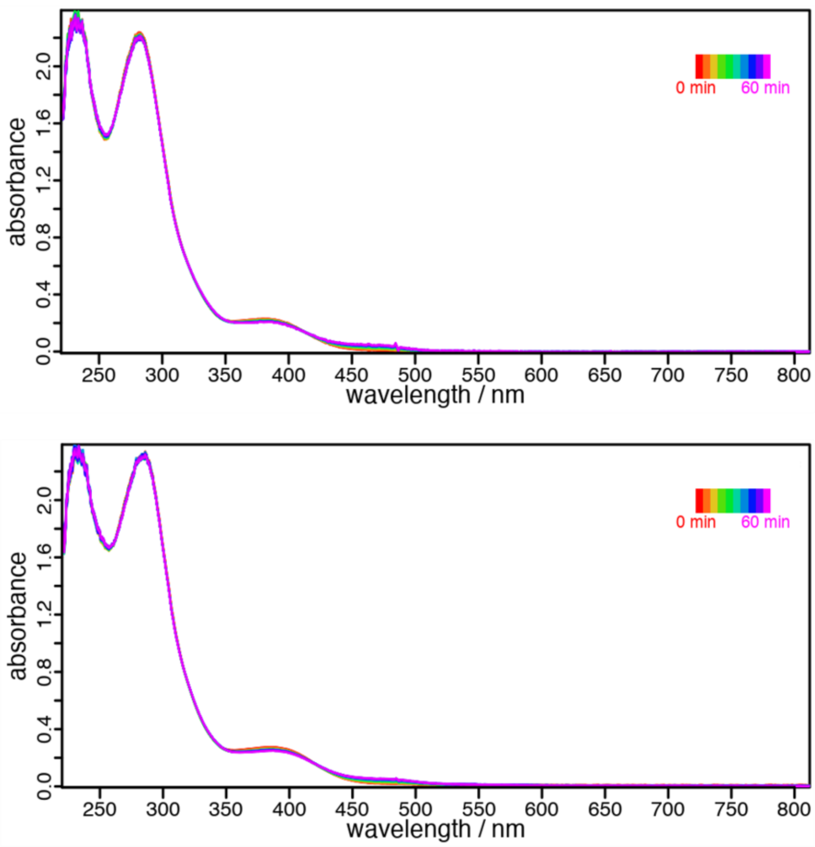

Figure S56. UV/vis absorption spectra of **C1-o** and **C2-m** (c = 0.02 mM) in ambient acetonitrile at room temperature over a time course of 1 h. The changes in absorbance around 485 nm are inherent to the lamp of the spectrometer used in the experiment.

14 Photocatalysis

Singlet oxygen formation

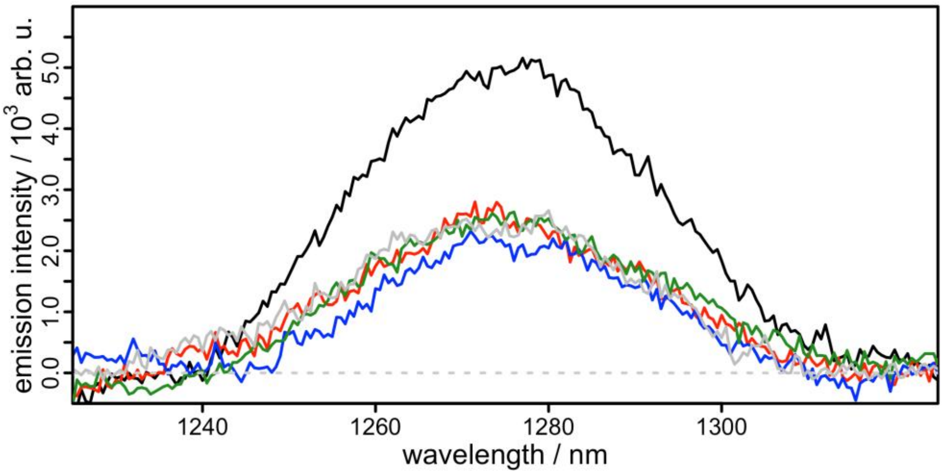

Figure S57. The emission spectra of the emission induced by the decay of catalytically generated $^1O_2$ at 1275 nm of **C1-o** (red), **C2-m** (blue), **C3-p** (green), **C4-ref** (grey) and the reference phenalenone (black). The spectra are baseline corrected at 1325 nm. The integration of the area under the emission curves was conducted within the wavelength range of 1240 to 1320 nm.

Catalytic oxygenation of DPF to DBE

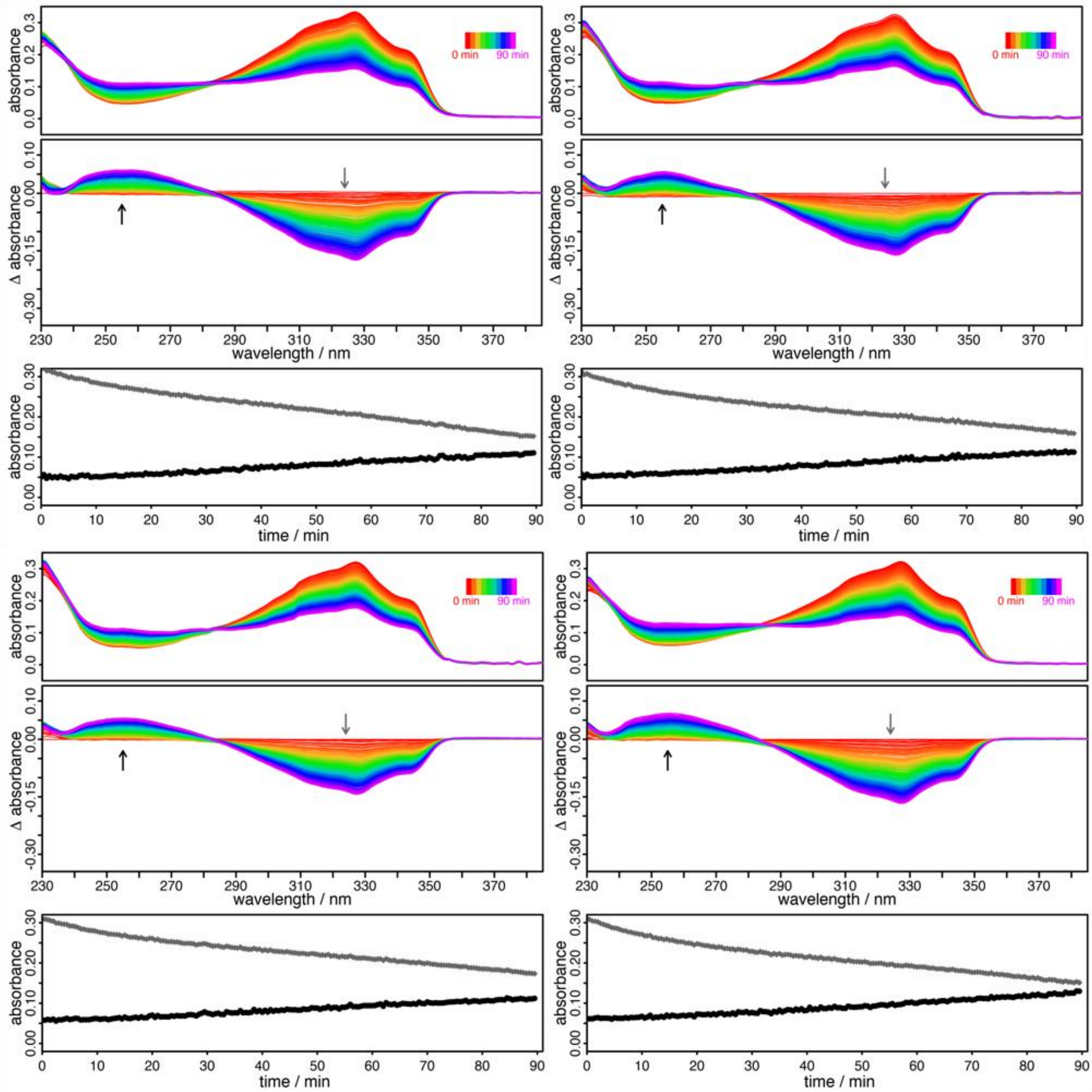

Figure S58. Summary of UV/vis absorption spectral changes of photocatalytic oxygenation of 2,5-diphenylfuran (DPF, c = $1.9 \cdot 10^{-5}$ M) in aerated dichloromethane under irradiation with a starlight lamp (0.095 W at cuvette, distance: 2 cm) over a time of 90 min. *In situ* UV/vis absorption spectra (top), the differential plots (middle) and the kinetic plots (bottom) of DPF (324 nm, grey dots) and DBE (255 nm, black dots) are displayed for each complex (c = $0.95 \cdot 10^{-6}$ M, **C1-o**, top left; **C2-m**, top right; **C3-p**, bottom left; **C4-ref**, bottom right).

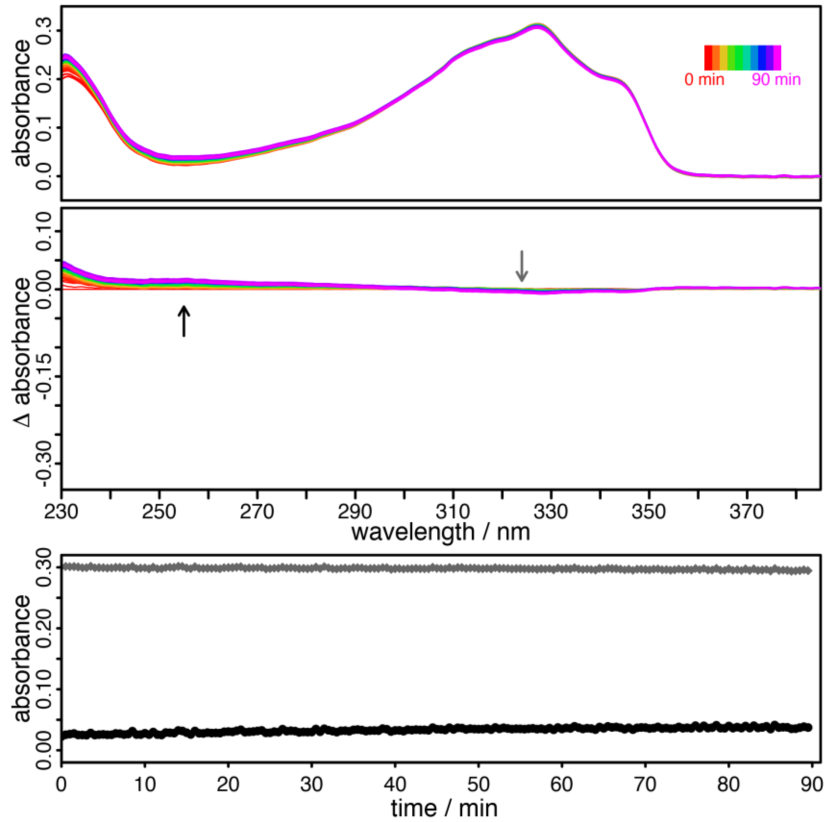

Figure S59. Self-photooxidation experiment of 2,5-diphenylfuran (DPF, c = $1.9 \cdot 10^{-5}$ M) in aerated dichloromethane under irradiation with a starlight lamp (0.095 W at cuvette, distance: 2 cm) over a time of 90 min without $^1O_2$ sensitizer. *In situ* UV/vis absorption spectra (top), the differential plots (middle) and the kinetic plots (bottom) of DPF (324 nm, grey dots) and DBE (255 nm, black dots).

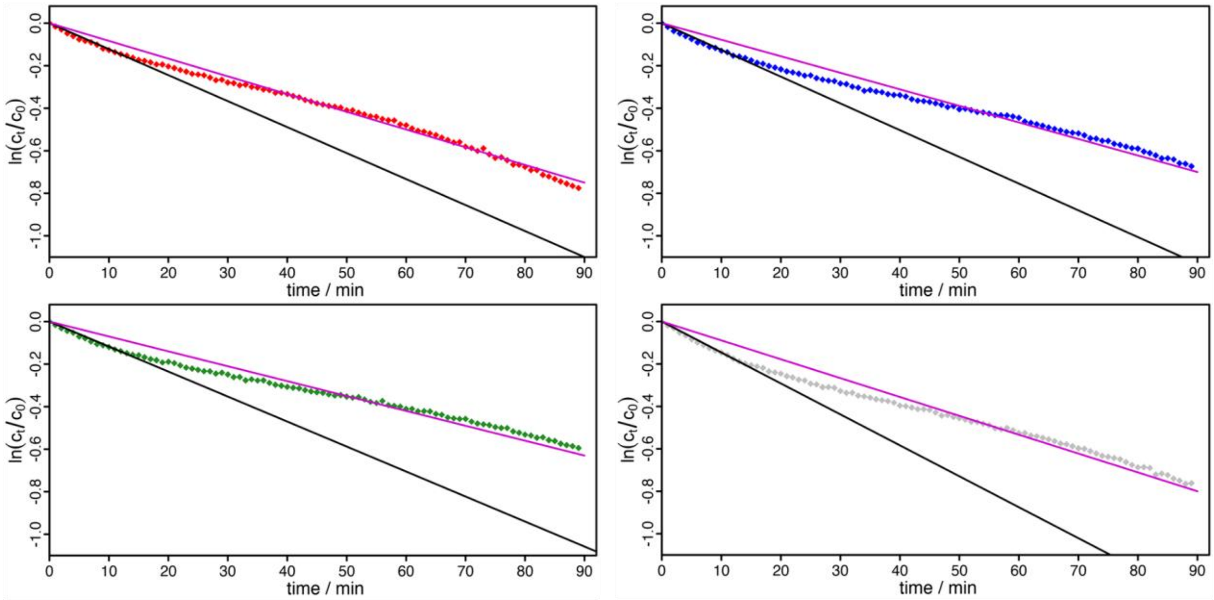

Figure S60. DPF conversion rates of **C1-o** (top left), **C2-m** (top right), **C3-p** (bottom left) and **C4-ref** (bottom right) with a first order fit ($\ln(\frac{c_t}{c_o})$) for the first 15 min (black line) and for the total 90 min (magenta line). The slope for the corresponding conversion rates ($k_{c1,2}$) are given in Table S26.

Table S26. Determined conversion rates ($k_{c1,2}$) of DPF after 15 min and 90 min.

Complex	k_{c1} $[10^{-4} s^{-1}]$ 15 min	k_{c2} $[10^{-4} s^{-1}]$ 90 min
**C1-o**	2.04	1.41
**C2-m**	2.10	1.30
**C3-p**	1.96	1.15
**C4-ref**	2.43	1.48

Stern-Volmer-Quenching

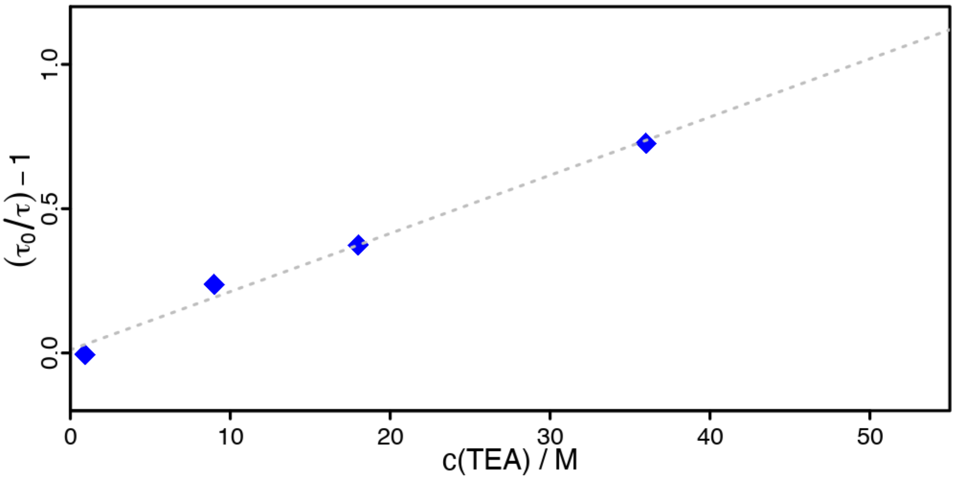

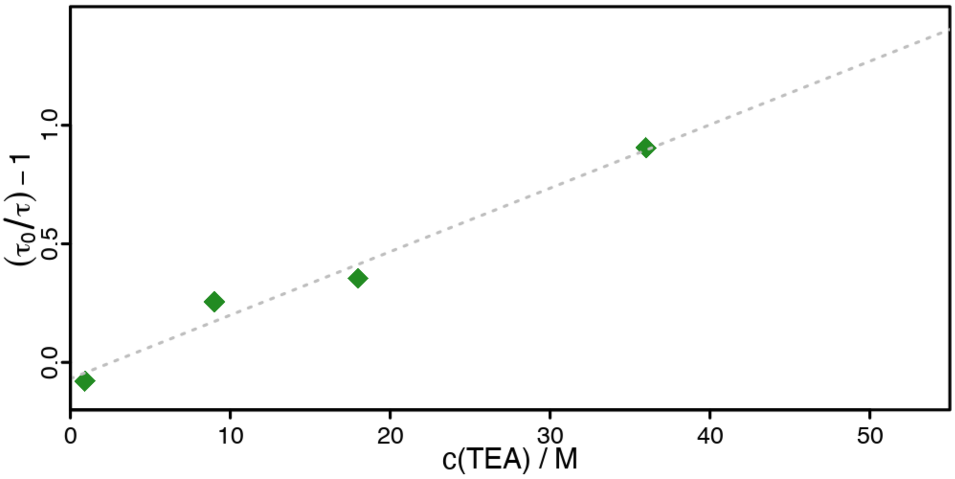

Figure S61. Stern-Volmer experiment for **C2-m** (top) and **C3-p** (bottom) in inert THF at room temperature. Plot show $\left(\frac{\tau_0}{\tau}\right) - 1$ (where τ_0 and τ are the respective lifetimes in presence and in absence of TEA) *vs.* [TEA] (blue/green diamonds). The kinetic constant for the reductive quenching (k_q) for **C2-m** ($k_q = 2.80 \cdot 10^6\ M^{-1} \cdot s^{-1}$) and **C3-p** ($k_q = 1.83 \cdot 10^6\ M^{-1} \cdot s^{-1}$) are determined from the slope of the Stern-Volmer plots.

Hydrogen evolution reaction

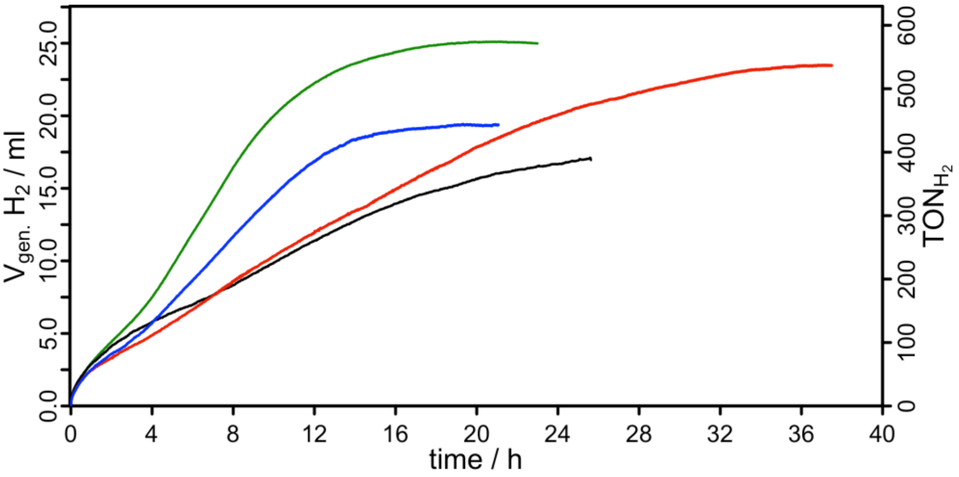

Figure S62. Full photocatalytic hydrogen evolution curves for the complexes **C1-o** (red), **C2-m** (blue), **C3-p** (green) and **C4-ref** (black) in the presence of $Fe_3(CO)_{12}$ as WRC and TEA as SD.

Hydrogen yields and turnover numbers (TONs) are only comparable to a limited extent across studies employing different catalytic conditions (*e.g*. light sources, irradiance, spectral distribution, reactor geometry, or concentrations). In particular, variations in the light source (photon energy and number of photons) can strongly affect performance. Reim *et al*.[22] showed that even minor adjustments to the illumination setup can markedly change the observed activity. Consequently, the absolute TONs reported for the reference complex **C4-ref** may differ from literature values or earlier work.[23] Within this study, however, all experimental parameters were kept constant, enabling a direct comparison of catalytic activities and TONs.

Photocatalytic reductive dehalogenation

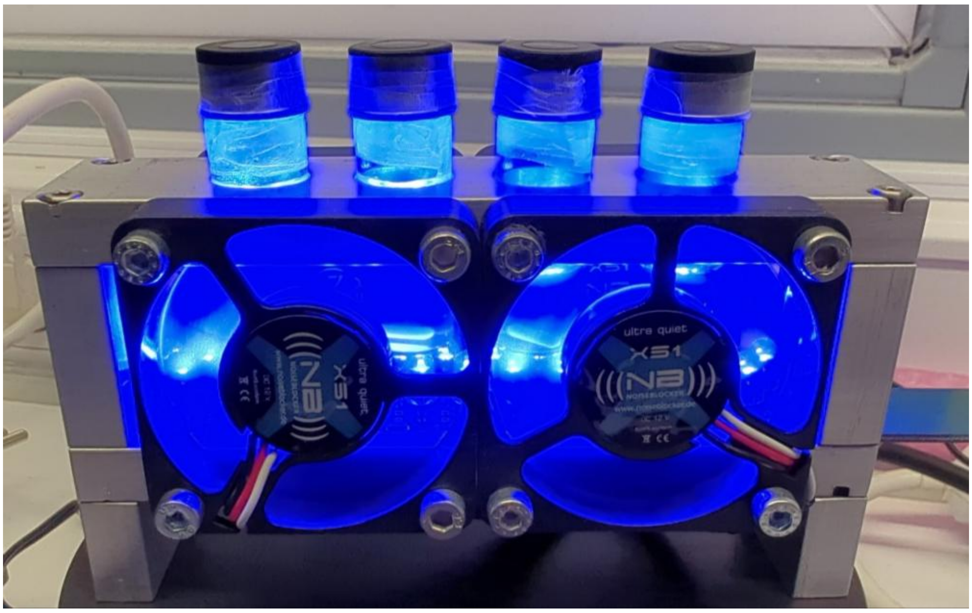

Figure S63. Picture of the photoreactor setup.

The used photoreactor (Fig. S63) accommodates four vials with a diameter of 1.5 cm, positioned 0.7 cm apart. To dissipate the generated heat, the reactor is cooled by four fans (two on each side), ensuring that temperature fluctuations remain within ±1 °C over a period of 5 h. The vials are placed directly on the LED (λ = 460 nm) used for irradiation. The emission spectrum of the LED is shown in Figure S64.

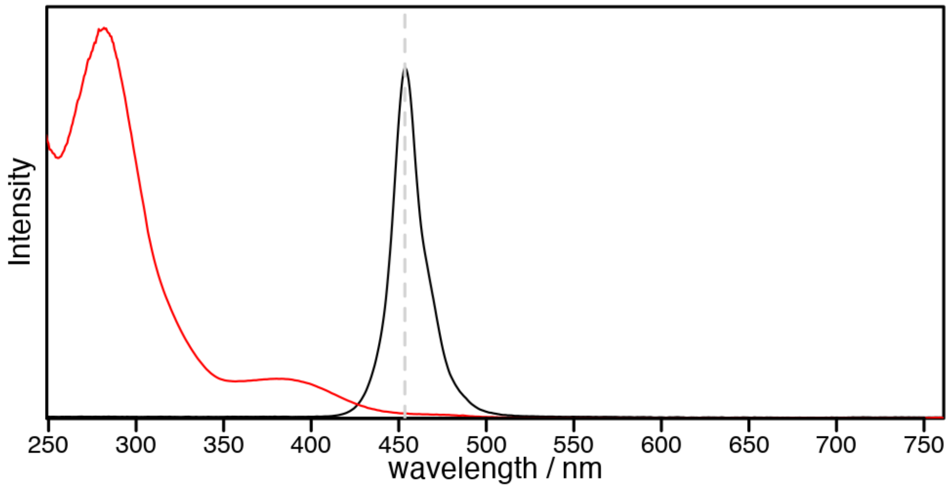

Figure S64. Overlap between UV/vis absorption spectra of **C1-o** (red solid line) and emission spectra of the used blue LED light (black solid line).

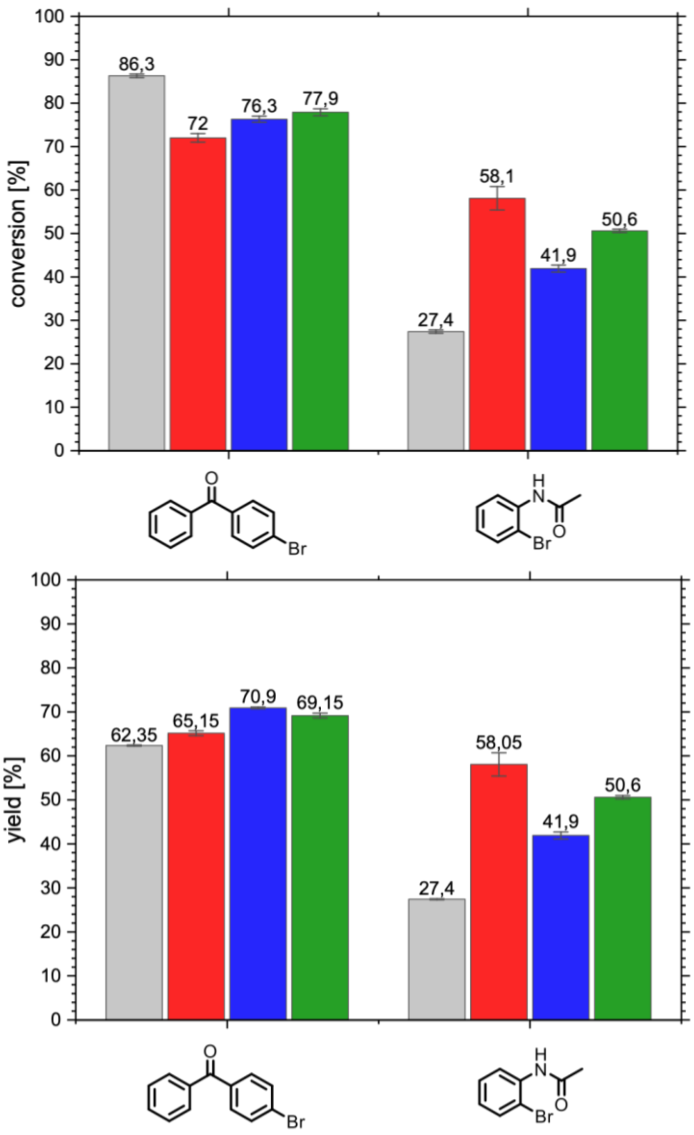

Figure S65. Conversion and yields in % with error bars of dehalogenated products after 15 minutes (**E1**, 4-bromobenzo-phenone) or 5 hours (**E2**, 2-bromoacetanilide) of **C1-o** (red), **C2-m** (blue), **C3-p** (green) and **C4-ref** (grey).

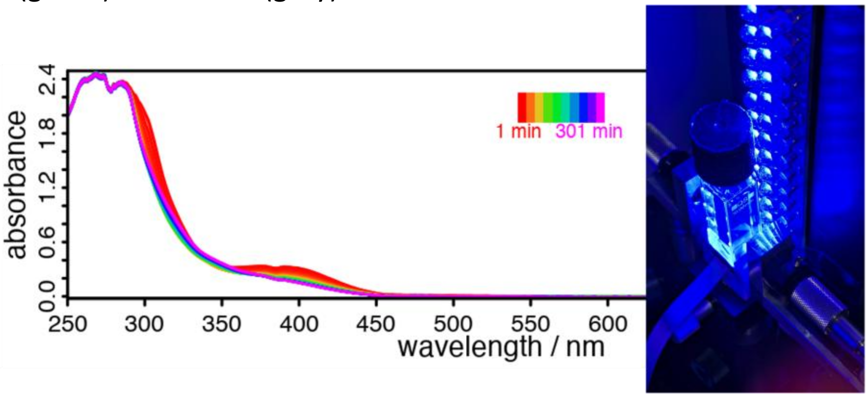

Figure S66. Photostability test of **C3-p** in catalytic condition under blue light irradiation at 460 nm, monitored by time-dependent UV/vis spectroscopy over 5 h.

15 References

1) Stalke, D.; Cryo crystal structure determination and application to intermediates, *Chem. Soc. Rev.* **1998**, *27*, 171–178.
2) CrysAlisPro, Rigaku Oxford Diffraction, Version 1.171.43.123a, **2024**.
3) Sheldrick, G. M.; SHELXT - Integrated space-group and crystal-structure determination. *Acta Cryst.* **2015**, *A71*, 3–8.
4) Sheldrick, G. M.; Crystal structure refinement with SHELXL. *Acta Cryst.* **2015**, *C71*, 3–8.
5) Sheldrick, G. M.; A short history of SHELX. *Acta Cryst.* **2008**, *A64*, 112–122.
6) Dolomanov, O. V.; Bourhis, L. J.; Gildea, R. J.; Howard, J. A. K.; Puschmann, H.; OLEX2: a complete structure solution, refinement and analysis program. *J. Appl. Cryst.* **2009**, *42*, 339–341.
7) Spek, A. L.; Structure validation in chemical crystallography, *Acta Cryst.* **2009**, *D65*, 148–155.
8) Macrae, C. F.; Sovago, I.; Cottrell, S. J.; Galek, P. T. A.; McCabe, P.; Pidcock, E.; Platings, M.; Shields, G. P.; Stevens, J. S.; Towler, M.; Wood, P. A.; Mercury 4.0: from visualization to analysis, design and prediction. *J. Appl. Cryst.* **2020**, *53*, 226-235.
9) Diamond – Crystal and Molecular Structure Visualization, Crystal Impact - H. Putz and K. Brandenburg GbR, Bonn, Germany, **2024.**
10) Gaussian 16, Revision A.03, Frisch, M. J.; Trucks, G. W.; Schlegel, H. B.; Scuseria, G. E.; Robb, M. A.; Cheeseman, J. R.; Scalmani, G.; Barone, V.; Petersson, G. A.; Nakatsuji, H.; Li, X.; Caricato, M.; Marenich, A. V.; Bloino, J.; Janesko, B. G.; Gomperts, R.; Mennucci, B.; Hratchian, H. P.; Ortiz, J. V.; Izmaylov, A. F.; Sonnenberg, J. L.; Williams-Young, D.; Ding, F.; Lipparini, F.; Egidi, F.; Goings, J.; Peng, B.; Petrone, A.; Henderson, T.; Ranasinghe, D.; Zakrzewski, V. G.; Gao, J.; Rega, N.; Zheng, G.; Liang, W.; Hada, M.; Ehara, M.; Toyota, K.; Fukuda, R.; Hasegawa, J.; Ishida, M.; Nakajima, T.; Honda, Y.; Kitao, O.; Nakai, H.; Vreven, T.; Throssell, K.; Montgomery, J. A., Jr.; Peralta, J. E.; Ogliaro, F.; Bearpark, M. J.; Heyd, J. J.; Brothers, E. N.; Kudin, K. N.; Staroverov, V. N.; Keith, T. A.; Kobayashi, R.; Normand, J.; Raghavachari, K.; Rendell, A. P.; Burant, J. C.; Iyengar, S. S.; Tomasi, J.; Cossi, M.; Millam, J. M.; Klene, M.; Adamo, C.; Cammi, R.; Ochterski, J. W.; Martin, R. L.; Morokuma, K.; Farkas, O.; Foresman, J. B.; Fox, D. J. Gaussian, Inc., Wallingford CT, **2016**.
11) TURBOMOLE V7.8 2024, a development of University of Karlsruhe and Forschungszentrum Karlsruhe GmbH, **1989-2007**, TURBOMOLE GmbH, since 2007; available from https://www.turbomole.org.
12) Lu, T.; Chen, F.; Multiwfn: A multifunctional wavefunction analyzer, *J. Comp. Chem*. **2012**, *33*, 580-592.
13) Humphrey, W.; Dalke, A.; Schulten, K. VMD - Visual Molecular Dynamics, *J. Mol. Graph.* **1996**, *14*, 33-38.
14) DecayFit - Fluorescence Decay Analysis Software 1.3, FluorTools, www.fluortools.com
15) Leitl, M. J.; Küchle, F.-R.; Mayer, H. A.; Wesemann, L.; Yersin, H. Brightly Blue and Green Emitting Cu(I) Dimers for Singlet Harvesting in OLEDs. *J. Phys. Chem. A* **2013**, *117*, 11823-11836.
16) Godard, J.; Brégier, F.; Arnoux, P.; Myrzakhmetov, B.; Champavier, Y.; Frochot, C.; Sol, V. New Phenalenone Derivatives: Synthesis and Evaluation of Their Singlet Oxygen Quantum Yield. *ACS Omega* **2020**, *5* (43), 28264–28272.
17) Doettinger, F.; Yang, Y.; Schmid, M.-A.; Frey, W.; Karnahl, M.; Tschierlei, S. Cross-Coupled Phenyl- and Alkynyl-Based Phenanthrolines and Their Effect on the Photophysical and Electrochemical Properties of Heteroleptic Cu(I) Photosensitizers. *Inorg. Chem.* **2021**, *60* (7), 5391–5401.
18) Chen, N.; Xia, L.; Lennox, A. J. J.; Sun, Y.; Chen, H.; Jin, H.; Junge, H.; Wu, Q.; Jia, J.; Beller, M.; Luo, S. Structure-Activated Copper Photosensitisers for Photocatalytic Water Reduction. *Chem. Eur. J.* **2017**, *23* (15), 3631–3636.
19) Lakowicz, J. R.; Principles of Fluorescence Spectroscopy. 3rd edition, Springer New York, NY, **2006**.
20) Rehm, D.; Weller, A. Kinetics of Fluorescence Quenching by Electron and H-Atom Transfer. *Isr. J. Chem.* Complexes. *Catal. Sci. Technol.* **2023**, *13* (14), 4092–4106.

20) Reim, I.; Wriedt, B.; Tastan, Ü.; Ziegenbalg, D.; Karnahl, M. *ChemistrySelect* **2018**, *3*, 2905–2911.
20) Mejía, E.; Luo, S.; Karnahl, M.; Friedrich, A.; Tschierlei, S.; Surkus, A.; Junge, H.; Gladiali, S.; Lochbrunner, S.; Beller, M. A Noble-Metal-Free System for Photocatalytic Hydrogen Production from Water. *Chem. Eur. J.* **2013**, *19* (47), 15972–15978.**1970**, *8* (2), 259–271.
21) Doettinger, F.; Kleeberg, C.; Queffélec, C.; Tschierlei, S.; Pellegrin, Y.; Karnahl, M. Rich or Poor: The Impact of Electron Donation and Withdrawal on the Photophysical and Photocatalytic Properties of Copper(I) Complexes. *Catal. Sci. Technol.* **2023**, *13* (14), 4092–4106.
22) Reim, I.; Wriedt, B.; Tastan, Ü.; Ziegenbalg, D.; Karnahl, M. *ChemistrySelect* **2018**, *3*, 2905–2911.
23) Mejía, E.; Luo, S.; Karnahl, M.; Friedrich, A.; Tschierlei, S.; Surkus, A.; Junge, H.; Gladiali, S.; Lochbrunner, S.; Beller, M. A Noble-Metal-Free System for Photocatalytic Hydrogen Production from Water. *Chem. Eur. J.* **2013**, *19* (47), 15972–15978.
